# A Multi-Omics Study of Neurodamage Induced by Growth-Stage Real-Time Air Pollution Exposure in Mice via the Microbiome–Gut–Brain Axis

**DOI:** 10.3390/toxics13040260

**Published:** 2025-03-29

**Authors:** Zijun Yang, Yi Zhang, Shanshan Ran, Jingyi Zhang, Fei Tian, Hui Shi, Shengtao Wei, Xiuxiu Li, Xinyue Li, Yonggui Gao, Guang Jia, Hualiang Lin, Zhangjian Chen, Zilong Zhang

**Affiliations:** 1 Department of Epidemiology, School of Public Health, Sun Yat-sen University, Guangzhou 510080, China; yangzj39@mail2.sysu.edu.cn (Z.Y.); ranshsh@mail2.sysu.edu.cn (S.R.); zhangjy563@mail2.sysu.edu.cn (J.Z.); tianf8@mail2.sysu.edu.cn (F.T.); shih39@mail2.sysu.edu.cn (H.S.); weisht3@mail2.sysu.edu.cn (S.W.); lixy856@mail2.sysu.edu.cn (X.L.); gaoyg5@mail2.sysu.edu.cn (Y.G.); linhualiang@mail.sysu.edu.cn (H.L.); 2 Department of Occupational and Environmental Health Sciences, School of Public Health, Peking University, Beijing 100191, China; 2411110207@pku.edu.cn (Y.Z.); jiaguangjia@bjmu.edu.cn (G.J.); 3 Department of Science and Education, Nanshan Maternity & Child Healthcare Hospital of Shenzhen, Shenzhen 518067, China; lixiuxiu1103@163.com

**Keywords:** air pollution, growth-stage, multi-omics, microbiome–gut–brain axis, neuronal damage

## Abstract

Air pollution has been widely recognized as a risk factor for neurological disorders, and the gut microbiome may play a mediating role. However, current evidence remains limited. In this study, a mouse model was employed with continuous exposure to real-time air pollution from conception to late adolescence. Effects of growth-stage air pollution exposure on the gut microbiome, host metabolites, and brain tissue were assessed. Pathological damage in the hippocampus and cortex was observed. Fecal metagenomic sequencing revealed alterations in both compositions and functions of the gut microbiome. Metabolic disturbances in unsaturated fatty acids and glycerophospholipids were identified in the intestine, serum, and brain tissues, with significant changes in metabolites (e.g., gamma-linolenic acid, alpha-linolenic acid, docosahexaenoic acid (DHA), phosphatidylethanolamine (PE), phosphatidylcholine (PC) and phosphatidylserine (PS). Serum levels of the pro-inflammatory mediator leukotriene C4 were also elevated. Correlation analysis identified a group of different gut microbiome species that were associated with host metabolites. Furthermore, mediation analysis showed that intestinal and serum metabolites mediated the associations between the key gut microbiome and brain microbiome. These findings indicate that the metabolic crosstalk in the gut–brain axis mediates the neuronal damage in mice induced by growth-stage air pollution exposure, potentially through pathways involving lipid metabolism and inflammation.

## 1. Introduction

Air pollution, especially fine particulate matter (PM_2.5_), has been widely recognized as a major threat to public health [1]. In recent years, the potential effects of air pollution on the nervous system have gained increasing attention [2,3]. A growing number of epidemiological studies have shown that exposure to air pollution during early life (e.g., pregnancy and infancy) is associated with increased risks of autism spectrum disorder (ASD), attention deficit hyperactivity disorder (ADHD), and other neurodevelopmental disorders (NDDs) [4,5,6], a group of diseases that not only seriously affect children’s cognition, behavior, and social abilities, but also have profound impacts on lifelong mental health and socioeconomics. However, a remaining question is that the specific mechanisms by which early-life air pollution exposure causes neurodevelopmental harm are largely unclear. A better understanding of these mechanisms is imperative for the development of effective interventions against NDDs.

In recent years, the description of the microbiome–gut–brain axis (MGBA) has provided a new theoretical perspective for understanding how environmental exposure, including air pollution, affects the nervous system [7]. The MGBA is a complex signaling network through which the gut microbiome interacts with the central nervous system via neural, immune, endocrine, and metabolic pathways. It is hypothesized that the gut microbiome can influence brain development and function as well as behaviors of the host by modulating immune responses, producing metabolites (e.g., fatty acids, neurotransmitter precursors), activating vagal nerve signals, and affecting neuroendocrine pathways [7,8]. Both the gut microbiome and the nervous system undergo critical development in growth stages, especially the early-life stage, and the MGBA plays an important role in maintaining neurodevelopmental homeostasis [9,10]. Studies have shown that air pollution, particularly PM_2.5_, significantly alters the diversity and function of the gut microbiome [11,12]. Furthermore, alterations in the microbiome induced by air pollution may further disrupt the nervous system [13,14,15]. However, the exact mechanisms by which the gut microbiome affects brain development following exposure to air pollution are largely unknown.

Metabolites, as both intermediates and endpoints of biological processes, can directly reflect biological disturbances within the body. Changes in brain metabolism are closely related to the pathogenesis of neurological diseases, including NDDs [16]. Previous studies have shown that air pollution could impact brain metabolism and gene expression, contributing to disease pathogenesis [17,18]. Moreover, brain metabolites are also influenced by the cross-talk of the MGBA [19]. Therefore, metabolites may be a key mechanism linking air pollution and the nervous system via the MGBA. However, such a hypothesis has not been examined yet.

In recent years, advances in integrated multi-omics techniques including metagenomics, metabolomics, and transcriptomics, provide a more comprehensive approach to study air pollution’s impact on the gut microbiome and various organ systems. In the present study, a mouse model was employed to systematically investigate the health impact of growth-stage air pollution exposure on brain development and the potential mediating effect of the gut microbiome, with a multi-omics approach incorporating gut metagenomics, metabolomics, and transcriptomics. The main aim was to reveal the role of the MGBA in the associations between air pollution exposure, neural damage in offspring, and the underlying mechanisms, which can inform the development of intervention strategies for NDDs.

## 2. Materials and Methods

### 2.1. Animals and Study Design

**Figure 1:**
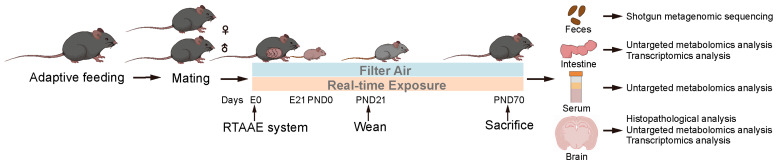
Schematic diagram depicting growth-stage real-time air pollution exposure of mice, as well as sample collection and testing conducted on the offspring mice. E: Embryonic day; PND: Postnatal day; RTAAE: real-time ambient air pollution exposure.

The present study used 6-week-old male and female C57BL/6 mice (average weight: 20 g for males and 18 g for females) purchased from the Peking University Health Science Center Laboratory Animal Center (Beijing, China). A total of 24 female mice and 12 male mice were randomly assigned to either the filtered air group (FA, as the control) or the real-world air pollution exposure group (Exp). After a one-week adaptation, males were housed individually, while females were housed according to their group. A three-day mating period was set, and two females were placed in the same cage with one male each night. Once the females became pregnant, they were assigned to either the exposure or control group for the subsequent exposure experiment. The start of embryonic development (E0.5) was determined when a vaginal plug was observed. After birth, the offspring mice were weaned on postnatal day 21 (PND 21), and continued to be housed in cages exposed to air pollution or filtered air until late adolescence. To avoid litter effects, one mouse per litter was selected in subsequent experiments. The mice were euthanized after reaching adulthood (10 weeks), and samples of feces, intestine, serum, and brain tissues were collected. Fecal samples were used for metagenomic sequencing, intestine samples were used for untargeted metabolomics and transcriptomics analysis, serum samples were used for untargeted metabolomics analysis, and brain tissue samples were used for untargeted metabolomics and transcriptomics analysis. The detailed experimental procedure is shown in Figure 1. In this study, the mice were housed in an environment with a 12 h light/dark cycle, at a temperature of 20–25 °C, with relative humidity maintained at 40–70%, and provided with standard chow and clean drinking water. The study protocol was approved by the Institutional Animal Care and Use Committee of Peking University Health Science Center (protocol code BCJD0130, approval date: 29 April 2023).

### 2.2. Real-Time Ambient Air Pollution Exposure (RTAAE) System

To simulate real-time environmental air pollution exposure, a whole-body RTAAE system was established. Detailed information about the RTAAE system, including the monitoring system, has been provided in our previous studies [20,21,22]. Briefly, the individual ventilated cages (IVCs) for the FA group were fitted with high-efficiency particulate air (HEPA) filters to supply clean air, while the IVCs for the exposed group had the filters removed and replaced with retention devices, allowing particles smaller than 2.5 μm to enter. The RTAAE system was set up 10 m above ground, and approximately 200 m from the North Fourth Ring Road in Beijing, an area with high traffic volume. According to our previous study [21], PM_2.5_ and O_3_ were the primary pollutants in the exposure cages. Composition analysis showed that PM_2.5_ mainly contained metal elements such as Fe, Na, and Ca, and the main water-soluble substances were NH_4_^+^ and Ca^2+^. In this study, the concentrations of air pollutants in the system were continuously monitored throughout the study. In particular, PM_2.5_ was collected using a BUCK LP-5 air sampling pump (AP Buck Inc., Orlando, FL, USA) with 37 mm quartz fiber filters at a flow rate of 1.70 L/min, and the concentration was determined by gravimetric analysis. O_3_ concentration ([O_3_]_8 h,max_) was measured using a Two Technology 220 Model real-time ozone concentration detector (2B Technologies, Boulder, CO, USA). Meanwhile, background air pollution (i.e., ambient air pollution) concentrations were obtained from the Olympic Sports Center Monitoring Station of the Beijing Environmental Protection Monitoring Center (approximately 4.5 km away from the study site) (http://www.bjmemc.com.cn/, URL accessed from 1 June to 30 September 2023).

### 2.3. Histopathological Analysis

Mice were euthanized after reaching adulthood, and brain tissues were immediately collected. The tissues were first immersed in 4% paraformaldehyde solution for fixation for at least 24 h, followed by paraffin embedding. Sections with a thickness of 4 μm from the prefrontal cortex and hippocampus were prepared and mounted on glass slides. The sections were then stained with hematoxylin and eosin (H&E). Finally, the slides were observed and analyzed using a Pannoramic MIDI digital slide scanner (3DHISTECH, Hungary) and digital slide viewing software (CaseViewer 2.4).

### 2.4. Gut Microbiome Assessment by Shotgun Metagenomic Sequencing

Fresh fecal samples (0.5 g, approximately four pellets) were collected from mice at early adulthood, and then rapidly frozen in liquid nitrogen and stored at −80 °C until metagenomic sequencing. First, genomic DNA was extracted from fecal samples using the E.Z.N.A.^®^ Stool DNA Kit D4015 (Omega, Norcross, GA, USA) following the manufacturer’ s instructions. The extracted DNA was assessed for purity and integrity using agarose gel electrophoresis (AGE) and quantified using a Qubit fluorometer (Thermo Fisher Scientific, Waltham, MA, USA). High-quality DNA was fragmented to ~350 bp, followed by end repair, A-tailing, adapter ligation, purification, and PCR amplification to construct sequencing libraries. Libraries passing quality control were sequenced on the Illumina PE150 platform (Illumina Inc., San Diego, CA, USA). Preprocessed sequencing data were assembled into contigs using MEGAHIT software (version 1.0.3). Assembly quality was assessed using SOAP2.21 to ensure data reliability. Gene prediction was performed using Prokka software (version 1.14.5) to identify functional genes in each metagenomic dataset. CD-HIT software (version 4.8.1) was used to remove redundant genes, yielding a set of non-redundant Uniq Genes. For gene abundance analysis, Salmon software (version 1.10.1) was used to calculate the transcripts per million (TPM) values of each gene and normalize them to obtain relative abundance. Functional annotation of non-redundant Uniq Genes was performed by comparing protein sequences to multiple databases, including the NCBI non-redundant protein database (NR, version 20170730). Taxonomic classification of Uniq Genes was determined by mapping NR-annotated genes to specific taxonomic ranks (kingdom, phylum, class, order, family, genus, species). The last common ancestor (LCA) method was applied to gene abundance data to estimate taxonomic abundance at various levels. Species abundance was calculated as the sum of all gene abundances assigned to that species. Finally, DEGseq R package (version 1.56.1) was used for differential analysis, with screening criteria of fold change ≥2 and FDR Q-value < 0.01 to identify differentially expressed genes under different conditions. *α*-Diversity indices (Shannon, Chao 1, Observed species, Simpson, ACE) were calculated using the Qiime (version 1.8.0) to evaluate gut microbiome richness and diversity. *β*-diversity was analyzed using Bray–Curtis distance, and differences in microbiome composition were evaluated through non-metric multidimensional scaling (NMDS) in the “vegan” R package (version 2.6-3). The linear discriminant analysis effect size (LEfSe) method was applied to identify significantly different microbes between groups from the phylum to species level. Microbes were classified and ranked based on their linear discriminant analysis (LDA) scores. Benjamini–Hochberg (BH) correction was performed to adjust for multiple testing and control the false discovery rate.

### 2.5. Untargeted Metabolomics Analysis

Liquid chromatography-mass spectrometry (LC-MS) was used for untargeted metabolomic analysis. After euthanasia, intestine, brain tissue, and serum samples were rapidly collected, flash-frozen in liquid nitrogen, and stored at −80 °C for subsequent metabolite extraction. Internal standard A0 was prepared by dissolving 10 mg of reserpine and 10 mg of chloramphenicol in 10 mL of acetonitrile at a concentration of 1 mg/mL. Then, 1 mL of internal standard A0 was diluted to 0.1 mg/mL to prepare internal standard A. Subsequently, 500 μL of internal standard A was added to 50 mL of acetonitrile to prepare solvent B. For serum samples, 50 μL of serum was mixed with 200 μL of solvent B. For intestine and brain tissue samples, 50 mg of tissue was homogenized with 200 μL of solvent B using liquid nitrogen grinding. Samples were centrifuged at 12,000 rpm for 10 min at 4 °C. The acetonitrile supernatant was collected, and 100 μL was used for polar metabolite analysis. Additionally, individual samples were pooled at a 1:1:1 ratio, and 10 μL was used to prepare a quality control (QC) sample. Next, samples were analyzed using the UPLC-Synapt XS system (Waters Corp., Milford, CT, USA). Ultra-performance liquid chromatography (UPLC) separation was performed using an HSS T3 column. Mobile phase A consisted of 0.1% formic acid in water, while mobile phase B contained 0.1% formic acid in acetonitrile. The flow rate was 0.4 mL/min, with a gradient elution from 98% A/2% B to 2% A/98% B. Separated samples were analyzed using the Synapt XS high-resolution mass spectrometer in positive and negative ion modes. The mass range was set to 50–1200 Da, with data acquired in MSE/DDA mode. The spray voltage was 2.0 kV, with a source temperature of 120 °C and a desolvation temperature of 450 °C. Raw data were converted to mzML format using MSConvertGUI software (version 3.0.24212-8361512) and imported into MS-DIAL software (version 4.24) for peak extraction, matching, and identification. Metabolites were identified by comparing with cationic and anionic standard databases in MS-Dial-based mass spectral libraries. Metabolite names were further confirmed using INCHIKEY and the Human Metabolome Database (HMDB, https://hmdb.ca, URL accessed on 20 October 2024). An unpaired Student’s *t*-test was used to analyze differences between groups. An orthogonal partial least squares discriminant analysis (OPLS-DA) model was constructed, and metabolites with VIP > 1 and *p* < 0.05 were selected as key differential metabolites. Enrichment and pathway analyses were performed using the MetaboAnalyst 6.0 platform (https://www.metaboanalyst.ca/, URL accessed on 20 October 2024), with pathway analysis based on the Kyoto Encyclopedia of Genes and Genomes (KEGG) database.

### 2.6. Transcriptomics Analysis

Intestinal and brain tissues were used for transcriptomics analysis. Total RNA was extracted using TRIzol^®^ reagent (Thermo Fisher Scientific, Carlsbad, CA, USA). RNA quality was assessed with ND-2000 (NanoDrop Technologies, Wilmington, DE, USA). and a 5300 Bioanalyzer (Agilent Technologies, Santa Clara, CA, USA, and only samples with OD260/280 = 1.8–2.2 and RQN ≥ 6.5 were selected for sequencing. RNA purification, reverse transcription, library construction, and sequencing were performed according to standard protocols. First, mRNA was enriched using Oligo(dT) magnetic beads, fragmented, and then used for double-stranded cDNA synthesis, end repair, phosphorylation, and adapter ligation to construct sequencing libraries. After library quantification, paired-end sequencing (PE150) was performed on the NovaSeq X Plus platform (Illumina, San Diego, CA, USA). Sequencing data were processed using fastp for quality control, removing low-quality reads. The cleaned reads were aligned to the mouse reference genome (GRCm38/mm10) using HISAT2, followed by transcriptome assembly and gene expression quantification with StringTie. Differential expression analysis was performed using DESeq2 with |log_2_FC| ≥ 1 and FDR Q-value < 0.05 as the criteria for defining differentially expressed genes (DEGs). KEGG pathway enrichment analysis of DEGs was conducted using Python’s scipy package (version 1.13.1).

### 2.7. Statistical Analysis

Data were presented as mean ± standard deviation (x ± SD). Group comparisons were performed using Student’s *t*-test or ANOVA for normally distributed data and the Wilcoxon rank-sum test or Kruskal–Wallis test for non-normally distributed data. Spearman correlation analysis was used to assess the relationships among the microbiome, intestinal metabolites, serum metabolites and brain metabolites. Mediation analysis was performed to assess whether intestinal and serum metabolites mediated the relationship between gut microbiome and brain metabolic changes. The proportion mediated was calculated as the ratio of indirect effects to total effects, quantifying the contribution of intestinal and serum metabolites. Mediation analyses were conducted using the “mediation” package (version 4.5.0) in R (version 4.4.2). Statistical analyses and data visualization were conducted using GraphPad Prism (version 9) and R (version 4.4.2). Statistical significance was set at *p* < 0.05. False discovery rate (FDR) correction for multiple comparisons was performed using the Benjamini–Hochberg (BH) method. Q-values were used for FDR adjustment in metagenomic and transcriptomic analyses.

## 3. Results

### 3.1. Level of Air Pollutants in the Exposure System

Figure S1 shows the distribution of the concentrations of PM_2.5_ and [O_3_]_8 h,max_ over the study period. The mean weekly concentration of PM_2.5_ was 12.56 μg/m^3^ inside the system and 24.41 μg/m^3^ in the ambient environment. The corresponding concentrations of [O3]_8 h,max_ were 105.49 μg/m^3^ and 135.76 μg/m^3^, respectively. In the filtered air group, the concentrations of PM_2.5_ and O_3_ were below the limits of detection (LODs) of 0.5 μg/m^3^ and 0.1 ppb, respectively.

### 3.2. Effects of Air Pollution Exposure on the Hippocampus and Cortex

**Figure 2:**
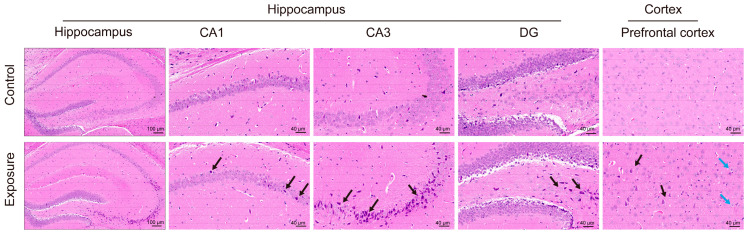
Pathological changes in the hippocampus and cortex following growth-stage air pollution exposure. Images of H&E staining in the hippocampus (scale bar = 100 μm), as well as pathological sections of the CA1, CA3, and DG regions of the hippocampus (scale bar = 40 μm), and cortical sections (scale bar = 40 μm). Black arrows indicate shrunken cytoplasm, reduced cell volume, and irregular shape; blue arrows highlight edema in pyramidal cells, characterized by cell swelling and pale cytoplasm.

Histopathological analysis revealed that growth-stage exposure to air pollution caused disorganized cell arrangement, neuronal shrinkage, necrosis, and edema in the hippocampal CA1 and CA3 regions of offspring mice. Additionally, the cortical tissue exhibited neuronal nuclear loss and edema (Figure 2).

**Figure 3:**
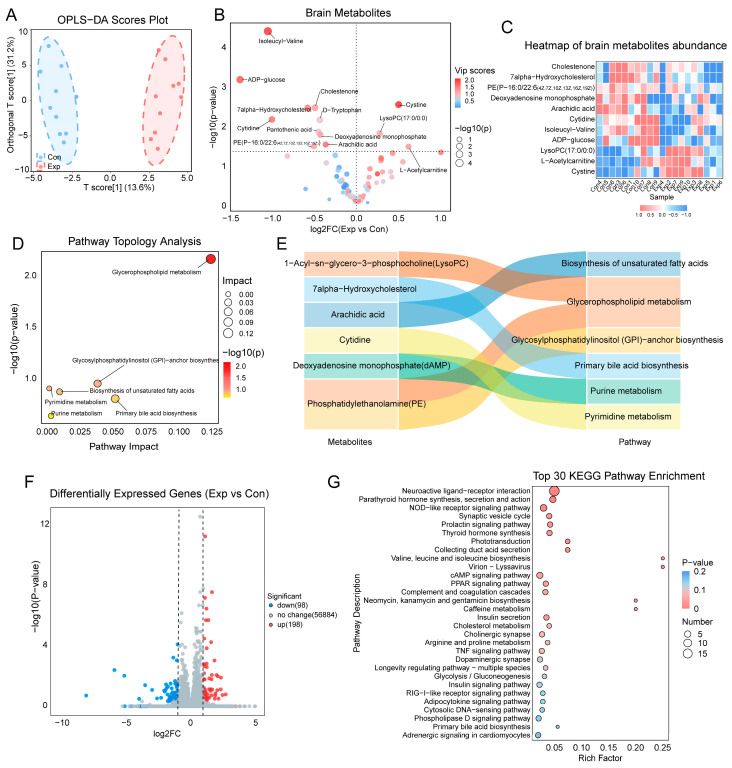
Alterations in metabolomics and transcriptomics in the hippocampus and cortex following growth-stage air pollution exposure. (**A**) OPLS-DA scores plot. (**B**) Volcano plot of brain metabolites, with the names of the top 13 metabolites based on VIP scores labeled. (**C**) Heatmap of brain metabolites abundance. (**D**) Topological analysis of differential metabolites in the KEGG pathway. (**E**) The correlations between differential metabolites and pathways. (**F**) Differentially expressed genes (DEGs): blue dots represent downregulated genes, red dots represent upregulated genes. (**G**) KEGG enrichment of DEGs: the y-axis represents enriched pathways, the x-axis shows the enrichment factor, circle size reflects gene count, and color indicates pathway *p*-values. Metabolomics: *n* = 10 per group. Transcriptomics: *n* = 6 per group.

To further examine metabolic alterations in the hippocampus and cortex, untargeted metabolomics analysis was conducted. OPLS-DA analysis (Figure 3A) was performed, and VIP scores were calculated for each metabolite based on their contribution to group separation (Figure S2). Using a VIP threshold > 1 and *p* < 0.05, 11 significantly altered metabolites were identified in the hippocampus and cortex of exposed mice (Figure 3B). A heatmap of key differential metabolites is presented in Figure 3C. KEGG pathway topological analysis (Figure 3D) identified significant disruptions in glycerophospholipid metabolism, glycosylphosphatidylinositol (GPI)-anchor biosynthesis, and unsaturated fatty acid biosynthesis in the exposed group. Key metabolites included PE(P-16:0/22:6(4Z,7Z,10Z,13Z,16Z,19Z)), lysophosphatidylcholine (LysoPC) (17:0/0:0), and arachidic acid (Figure 3E). To investigate potential sex differences, separate analyses were performed for male and female mice. Significant metabolic alterations were still observed in both sexes following exposure. Differentially altered metabolites were primarily associated with pathways such as purine metabolism, pyrimidine metabolism, glycerophospholipid metabolism, linoleic acid metabolism, and unsaturated fatty acid biosynthesis (Figure S3).

To assess molecular alterations in the hippocampus and cortex, RNA sequencing was performed. Under the criteria of |log_2_(FC)| > 1 and Q-value < 0.05, a total of 198 significantly upregulated genes and 98 downregulated genes were identified (Figure 3F). KEGG enrichment analysis (Figure 3G) revealed significant enrichment in neural pathways (e.g., neuroactive ligand–receptor interaction, synaptic vesicle cycle), lipid metabolism pathways (e.g., PPAR signaling, phospholipase D signaling), and inflammatory pathways (e.g., NOD-like receptor signaling). A total of 34 key differentially expressed genes were identified in these pathways, including Gabrq, Brs3, Gzma, C3, Ltb4r1, Pck1, Acox2, Angptl4, Avpr1a, Dgkk, and Agt. Collectively, metabolomics and transcriptomics results indicate that air pollution exposure during growth stages significantly alters lipid metabolism pathways, including glycerophospholipid metabolism and unsaturated fatty acid biosynthesis, as well as pathways related to neuro-communication and inflammation in the brain.

### 3.3. Alterations in the Gut Microbiome Induced by Air Pollution Exposure

**Figure 4:**
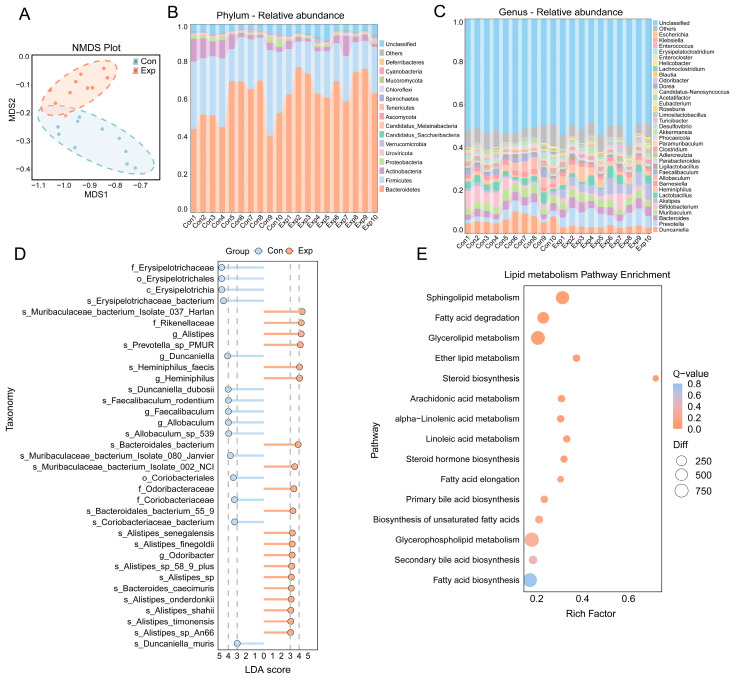
Effect of growth-stage air pollution exposure on gut microbiome, revealed by metagenomic sequencing. (**A**) NMDS plot of gut microbiome. (**B**,**C**) Relative abundance of microbiome at the phylum and genus levels for the samples. (**D**) Differential bacterial taxa identified by LEfSe analysis (Kruskal–Wallis test, *P_FDR_* < 0.05 with Benjamini–Hochberg correction) with LDA scores ≥ 3. (**E**) KEGG enrichment of DEGs. The y-axis represents enriched pathways, the x-axis shows the enrichment factor, circle size reflects gene count, and color indicates pathway Q-values. *n* = 10 per group.

NMDS analysis revealed some differences in the microbiome community structure between the exposed and control groups following exposure to air pollution (Figure 4A). Compositions of the microbiome at the phylum and genus levels are depicted in Figure 4B,C. No significant differences were found in the *α*-diversity indices between the two groups (*p* > 0.05) (Table 1). LEfSe analysis revealed 35 taxa with an LDA score greater than 3, among which 15 taxa were more highly expressed in the control group, while 20 taxa were more highly expressed in the exposed group (*P_FDR_* < 0.05) (Figure 4D). The relative abundance heatmap of the microbiome in the samples is shown in Figure S4. KEGG pathway enrichment analysis of differentially expressed genes from metagenomic sequencing showed significant enrichment in lipid metabolism pathways in the exposed mice (Figure 4E). Both male and female mice exhibited changes in the microbiome structure and lipid-related functional alterations following exposure (Figure S5).

### 3.4. Metabolomic and Transcriptomic Alterations in the Intestine

**Figure 5:**
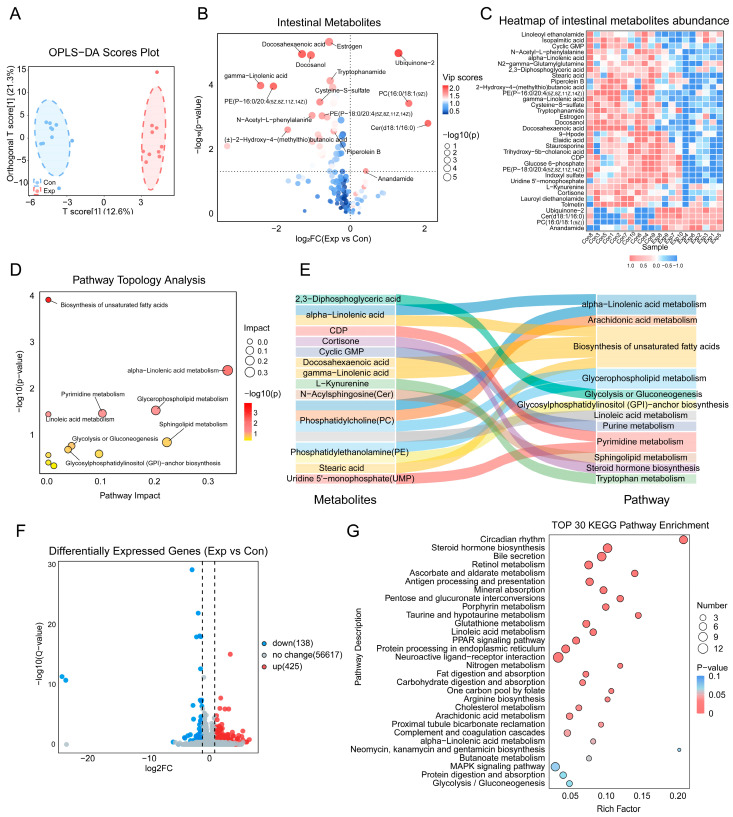
Metabolomic and transcriptomic alterations in the intestine. (**A**) OPLS-DA scores plot. (**B**) Volcano plot of intestinal metabolites, with the names of the top 15 metabolites based on VIP scores labeled. (**C**) Heatmap of intestinal metabolites abundance. (**D**) Topological analysis of differential metabolites in the KEGG pathway. (**E**) Correlations between differential metabolites and pathways. (**F**) Differentially expressed genes (DEGs): blue dots represent downregulated genes, red dots represent upregulated genes. (**G**) KEGG enrichment of DEGs: the y-axis represents enriched pathways, the x-axis shows the enrichment factor, circle size reflects gene count, and color indicates pathway *p*-values. Metabolomics: *n* = 10 per group. Transcriptomics: *n* = 6 per group.

OPLS-DA results from untargeted metabolomics analysis on intestine tissues are shown in Figure 5A. Based on the differential contributions, VIP scores for each metabolite were obtained (Figure S6). Under the screening thresholds of VIP > 1 and *p* < 0.05, 34 metabolites that were significantly different in the intestines of the two groups were identified (Figure 5B). The sample expression heatmap of key differential metabolites is shown in Figure 5C. KEGG pathway topological analysis (Figure 5D) showed that in the exposed group, significant changes occurred in pathways related to unsaturated fatty acid biosynthesis, alpha-linolenic acid metabolism, glycerophospholipid metabolism, pyrimidine metabolism, linoleic acid metabolism, and sphingolipid metabolism. In the separate sex analyses, similar results were also observed (Figure S7). Key differential metabolites included alpha-linolenic acid, DHA, gamma-linolenic acid, PC (16:0/18:1(9Z)), PE (P-18:0/20:4(5Z,8Z,11Z,14Z)), stearic acid and N-Acylsphingosine (Figure 5E).

In transcriptome analysis, a total of 425 significantly upregulated genes and 138 downregulated genes were identified (Figure 5F) using the criteria of |log_2_(FC)| > 1 and Q-value < 0.05. KEGG enrichment analysis indicated significant enrichment in pathways related to unsaturated fatty acid biosynthesis, including linoleic acid metabolism, arachidonic acid metabolism, alpha-linolenic acid metabolism, and the PPAR signaling pathway (Figure 5G). Key differential genes included Cyp3a13, Cyp2e1, Cyp3a25, Pla2g12b, Ggt1, Aloxe3, Acaa1b, Plin5, Me3, Hmgcs2, and Plin1. Taken together, metabolomics and transcriptomics results indicate that growth-stage air pollution exposure significantly alters unsaturated fatty acid biosynthesis, alpha-linolenic acid metabolism, and glycerophospholipid metabolism in the mouse intestine.

### 3.5. Serum Metabolomic Alterations

**Figure 6:**
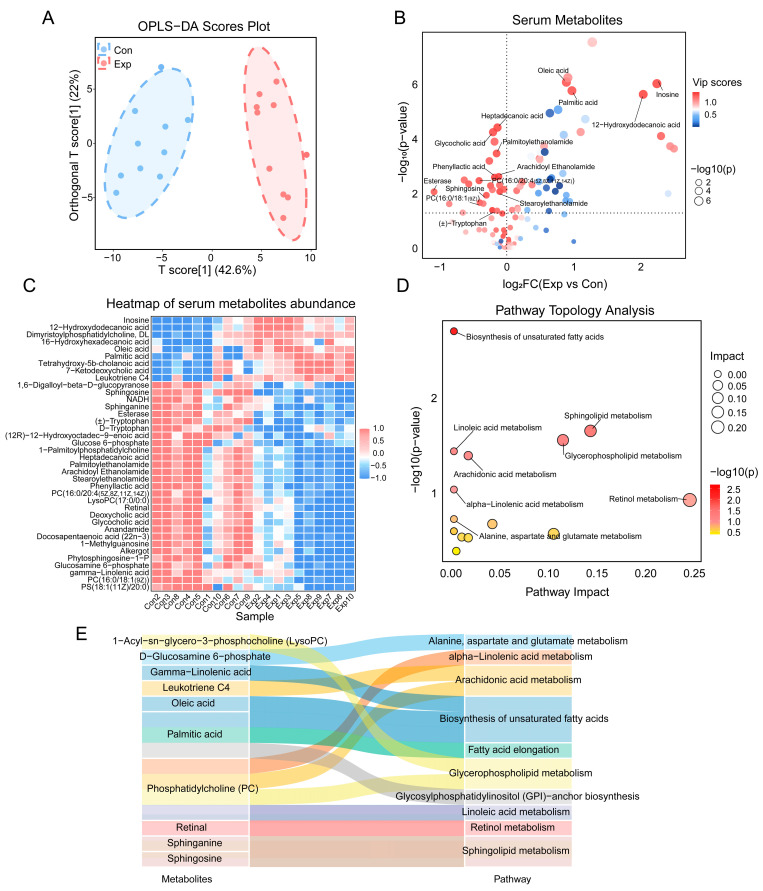
Metabolomic alterations in serum. (**A**) OPLS-DA scores plot. (**B**) Volcano plot of serum metabolites, with the names of the top 15 metabolites based on VIP scores labeled. (**C**) Heatmap of serum metabolites abundance. (**D**) Topological analysis of differential metabolites in the KEGG pathway. (**E**) Correlations between differential metabolites and pathways.

The OPLS-DA results from the untargeted metabolomics analysis of serum, including the VIP scores for metabolites, are shown in Figure 6A and Figure S8. Under the screening criteria of VIP > 1 and *p* < 0.05, 38 significantly different metabolites were identified in the serum of exposed and control mice (Figure 6B). The sample expression heatmap of key differential metabolites is shown in Figure 6C. KEGG pathway topological analysis (Figure 6D) showed significant changes in sphingolipid metabolism, unsaturated fatty acid biosynthesis, glycerophospholipid metabolism, arachidonic acid metabolism, linolenic acid metabolism and arachidonic acid metabolism in the exposed group. Key differential metabolites included sphinganine, sphingosine, gamma-linolenic acid, oleic acid, palmitic acid, PC (16:0/20:4(5Z,8Z,11Z,14Z)), PC (16:0/18:1(9Z)), LysoPC (18:1(9Z)/0:0), PS (18:1(11Z)/20:0), and leukotriene C4 (Figure 6E). Further analysis revealed a significant increase in saturated fatty acids (oleic acid, palmitic acid) and the pro-inflammatory mediator leukotriene C4 in the exposed group, whereas sphingolipid metabolites (sphinganine and sphingosine), unsaturated fatty acid (gamma-linolenic acid), and lipid metabolites (PC(16:0/20:4(5Z,8Z,11Z,14Z)), PC(16:0/18:1(9Z)), LysoPC(18:1(9Z)/0:0), PS(18:1(11Z)/20:0)) were significantly reduced. As leukotriene C4, a metabolite of the arachidonic acid pathway, is a potent pro-inflammatory mediator, its upregulation may indicate systemic inflammation [23]. In the separate sex analyses, similar results were also observed (Figure S9). The findings about serum metabolic changes suggest that air pollution exposure during growth stages may disrupt lipid-related metabolic pathways (e.g., unsaturated fatty acid, phospholipid, and sphingolipid metabolism), trigger systemic inflammation, and impair physiological homeostasis.

### 3.6. Correlation Between the Gut Microbiome and Host Metabolome

**Figure 7:**
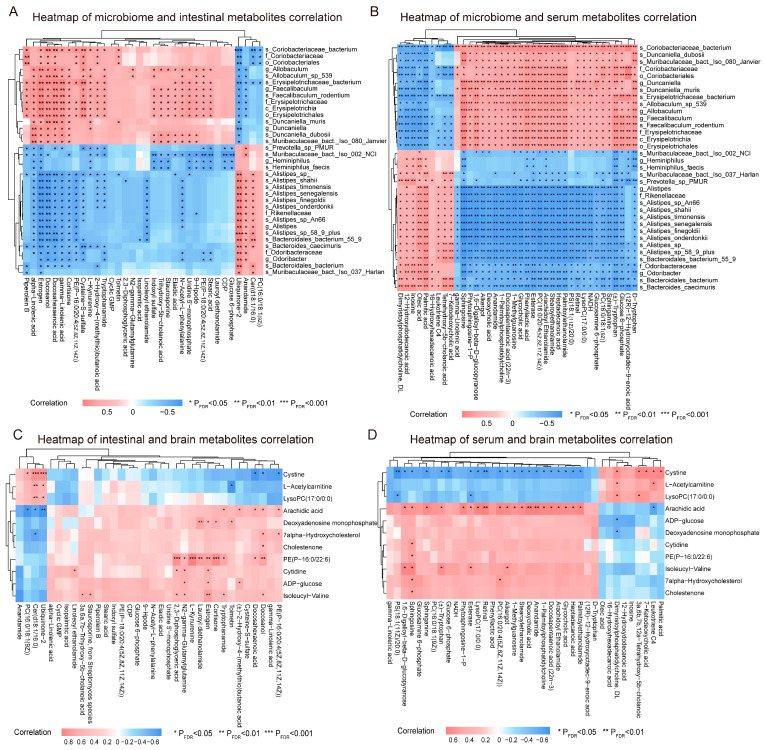
Correlation between the gut microbiome and host metabolome. (**A**) Heatmap of Spearman correlation analysis between differential taxa identified by LEfSe analysis (LDA ≥ 3) and differential intestinal metabolites. (**B**) Heatmap of Spearman correlation analysis between differentially identified taxa (LDA ≥ 3) from LEfSe analysis and differential serum metabolites. (**C**) Heatmap of Spearman correlation analysis between different intestinal metabolites and different brain metabolites. (**D**) Heatmap of Spearman correlation analysis between different serum metabolites and different brain metabolites. *n* = 10 per group. *P_FDR_* indicates the *p*-value adjusted using the Benjamini–Hochberg method. * *P_FDR_* < 0.05, ** *P_FDR_* < 0.01, *** *P_FDR_* < 0.001.

Spearman correlation analysis revealed significant associations between key intestinal metabolites and differences in the microbiome (*P_FDR_* < 0.05) (Figure 7A). For example, some microbes (e.g., *Alistipes*.*sp*, g_*Faecalibaculum*, *s_Coriobacteriaceae_bacterium* and *f_Erysipelotrichaceae*) were significantly associated with lipid-related metabolites (e.g., PE(P−16:0/20:4(5Z,8Z,11Z,14Z)), PC(16:0/18:1(9Z)), PS(18:1(11Z)/20:0), ceramide (Cer) (d18:1/16:0), DHA and gamma−Linolenic acid) (*P_FDR_* < 0.05). Significant associations between gut microbiome and serum metabolites were also observed (*P_FDR_* < 0.05) (Figure 7B). Notably, *Alistipes.sp* and *s_Bacteroidales_caecimuris* were positively correlated with the pro-inflammatory metabolite leukotriene C4 in serum (*P_FDR_* < 0.05), but negatively correlated with sphinganine, sphingosine, gamma-linolenic acid, PS, PC and LysoPC (*P_FDR_* < 0.05). Conversely, *s_Coriobacteriaceae_bacterium* and *o_Coriobacteriales* exhibited a negative correlation with leukotriene C4 (*P_FDR_* < 0.05) and a positive correlation with these metabolites (*P_FDR_* < 0.05).

Additionally, several intestinal and serum metabolites were significantly correlated with metabolic changes in the brain, including PE (P−16:0/22:6(4Z,7Z,10Z,13Z,16Z,19Z)), Arachidic acid, LysoPC (17:0/0:0), and Cystine (*P_FDR_* < 0.05) (Figure 7C,D).

### 3.7. Mediating Role of Intestinal and Serum Metabolites

Mediation analysis was conducted to explore the mediating effects of intestinal and serum metabolites in the relationship between differentially abundant microbiome (with LDA scores greater than 3) and brain metabolic changes. The results revealed that intestinal and serum metabolites mediated the effects of microbial taxa on different brain metabolites (proportion mediated: 9.5–99.6%, *p* < 0.05) (Table S1). Specifically, 13 metabolites (intestinal Cer(d18:1/16:0) and Ubiquinone-2, serum metabolites such as PS(18:1(11Z)/20:0), Sphingosine, gamma-Linolenic acid, Leukotriene C4, esterase, dimyristoylphosphatidylcholine, 16-Hydroxyhexadecanoic acid, Phenyllactic acid, arachidoyl ethanolamide, palmitoylethanolamide, phenyllactic acid, and heptadecanoic acid) mediated the influence of 22 key microbial taxa (*f_Coriobacteriaceae*, *f_Rikenellaceae*, *o_Coriobacteriales*, *g_Duncaniella*, *g_Alistipes, g_Heminiphilus*, *g_Allobaculum, s_Muribaculaceae_bacterium_Isolate_037_Harlan*, *s_Bacteroidales_bacterium, s_Bacteroidales_bacterium_55_9*, *s_Coriobacteriaceae_bacterium*, *s_Alistipes_sp_58_9_plus*, *s_Alistipes_sp*, *s_Bacteroides_caecimuris*, *s_Duncaniella_dubosii*, *s_Alistipes_senegalensis*, *s_Alistipes_onderdonkii*, *s_Allobaculum_sp_539*, *s_Alistipes_finegoldii*, *s_Alistipes_shahii*, *s_Alistipes_timonensis*, *s_Alistipes_sp_An66*) on brain LysoPC(17:0/0:0) and brain_PE(P-16:0/22:6(4Z,7Z,10Z,13Z,16Z,19Z)) metabolic changes (proportion mediated: 32.7–98.7%, *p* < 0.05) (Table 2).

## 4. Discussion

To our knowledge, this is among the first studies to integrate gut metagenomics, metabolomics, and transcriptomics techniques to comprehensively examine the neurological impact of growth-stage air pollution exposure and the mediating effects of the gut microbiome. This study demonstrated that exposure to real-time ambient air pollution during growth stages induced damage in the hippocampus and cortex of offspring mice, along with alterations in brain glycerophospholipid metabolism. Additionally, changes in the gut microbiome and lipid metabolism, unsaturated fatty acid metabolism, and inflammatory signaling pathways across multiple organs were observed. Such changes partially mediated the detrimental effects of air pollution on the nervous system.

Growth stage is a crucial period for neurodevelopment, and epidemiological studies have shown that exposure to air pollution during pregnancy and the postnatal period can impact the neurodevelopment of offspring [4,6,24,25,26]. Using a whole-body real-time exposure system to simulate real-world environmental conditions, the present study revealed that exposure to ambient air pollution during growth stages induced pathological changes in the cortical and hippocampal regions of mouse brain tissue, including disordered cell arrangement, neuronal damage, and edema. This is generally consistent with previous studies [17,27].

Although previous studies have clearly revealed the direct toxic effects (e.g., cognitive dysfunction and pathological changes) of air pollution on the central nervous system (CNS) [28,29,30], the exact mechanisms by which air pollution leads to neural injury remain largely unclear. The MGBA axis interaction may be a key mechanism involved. Previous studies have demonstrated that air pollution, including PM_2.5_, NO_2_, and O_3_, significantly alters the composition and function of the gut microbiome [31,32,33]. In this study, growth-stage exposure to air pollution significantly altered gut microbiome composition in mice. In particular, metagenomic analysis revealed that in the exposed group, the relative abundance of *s_Muribaculaceae_bacterium_Isolate_037_Harlan_*, *f_Rikenellaceae*, *g_Alistipes, s_Prevotella_sp_PMUR* increased, while *f_Erysipelotrichaceae*, *s_Erysipelotrichaceae_bacterium*, *g_Duncaniell* and *s_Duncaniella_dubosii* decreased. Previous studies have shown that these microbial taxa are closely linked to the health and disease status of the host and may have impacts on host metabolism and immune homeostasis. For example, studies have indicated that *Alistipes* is closely associated with colitis, and it is also linked to stress and depression [34,35]. These microbial changes may have profound effects on host metabolism and immune homeostasis, which could in turn impact brain development and function.

Evidence from both humans and animals has shown that air pollution can lead to metabolic disruptions [18,36], and changes in brain metabolism play a key role in the pathogenesis of neurological diseases [16]. At the same time, metabolites are key mediators for the interactions between the microbiome and host in the framework of MGBA. This study indicates that the changes in the gut microbiome induced by air pollution exposure are closely related to the host’s metabolites. In addition to pathological damage in brain tissue, metabolomic analysis further revealed significant alterations in glycerophospholipid metabolism pathways. Such alterations were also observed in the intestine and serum. Meanwhile, functional analysis of the gut microbiome also revealed significant alterations in lipid-related metabolic pathways in different taxa, suggesting that lipid metabolism may play an important role. Furthermore, our mediation analysis suggests that intestinal and serum metabolites may mediate the impact of the microbiome on brain lipid metabolites (e.g., PE and PC), linked to glycerophospholipid metabolism. Therefore, these findings suggest that glycerophospholipid metabolism may represent an important altered cross-talk pathway in the microbiome–gut–brain axis, contributing to neurodamage caused by air pollution exposure during the growth stage.

Previous evidence from human and animal studies has also shown that air pollution, particularly PM_2.5_, leads to disruptions in glycerophospholipid and sphingolipid metabolism [18,36]. Glycerophospholipids are major components of eukaryotic cell membranes, with PC and PE being the most abundant [37]. PC constitutes approximately 95% of total choline in most tissues, while PE is the second most abundant glycerophospholipid in cell membranes [37]. Other minor phospholipids, such as phosphatidylinositol (PI) and PS, are also involved in the structure of biological membranes. Glycerophospholipid metabolism is particularly important in the central nervous system, as it is a major component of neuronal membranes and myelin sheaths, maintaining neuronal stability and membrane fluidity [38]. Previous research has indicated that glycerophospholipid metabolism is disrupted in Alzheimer’s disease (AD) patients and AD mouse models [19,39]. The gastrointestinal tract is the primary source of glycerophospholipids for brain neural membranes [40]. Recent studies have shown that disruptions in glycerophospholipid metabolism are closely associated with the microbiome–gut–brain axis in depression patients [41,42]. Therefore, disruptions in glycerophospholipid metabolism may be a key mechanism underlying neural dysfunction and could serve as an important metabolite in the gut–brain axis. In the present study, transcriptomic analysis of brain tissue further revealed that air pollution exposure activated several neuro-related pathways, particularly neuroactive ligand–receptor interactions, synaptic vesicle cycling, and phospholipase D signaling pathways, which may be related to changes in membrane fluidity and neuronal signal transmission. Notably, changes in the expression of Avpr1a (vasopressin receptor 1A), Dgkk (diacylglycerol kinase K), and Agt (angiotensinogen) were closely linked to glycerophospholipid metabolism. These genes may influence the stability of brain cell membranes and neuronal signal transmission. Additionally, changes in the expression of Gabrq (GABA receptor q subunit) and Chrna6 (nicotinic acetylcholine receptor α6 subunit) could further affect neurotransmitter function and alter neuronal signal transmission. In summary, our findings suggest that air pollution may disrupt brain homeostasis and impair neural function by altering glycerophospholipid metabolism and related signaling pathways, with the gut–brain axis playing a key role in this process.

Notably, significant alterations in the unsaturated fatty acid biosynthesis pathway were also observed in the intestine, serum, and brain tissue in the present study. Mediation analysis suggests that these changes in intestine and serum partially mediated the alterations in brain lipids. Specifically, significant alterations in several fatty acids were observed in the intestine, serum, and brain, including alpha-linolenic acid, gamma-linolenic acid, DHA, oleic acid, palmitic acid, and arachidonic acid. These metabolites play crucial roles in immune regulation, lipid balance, and neural health. For instance, DHA, alpha-linolenic acid, and gamma-linolenic acid are involved in regulating inflammation and oxidative stress [43,44,45,46]. Fatty acids are essential components of lipids, and changes in fatty acid levels are closely linked to alterations in brain lipid metabolism. Also, transcriptomic analysis further revealed changes in PPAR signaling pathways, linoleic acid metabolism, and arachidonic acid metabolism in the intestine. Key differentially expressed genes, such as Acaa1b (acetyl-CoA carboxylase 1B), which is critical for fatty acid synthesis, may affect lipid metabolism and function in both the body and brain [47]. Additionally, Pla2g12b, a member of the phospholipase A2 family involved in lipid metabolism, particularly in the hydrolysis of phospholipids, may influence brain lipid metabolism by regulating lipid distribution and transport [48]. Therefore, the biosynthesis of unsaturated fatty acids may be an important pathway in the microbiome–gut–brain metabolic cross-talk.

The role of inflammation in neurological diseases has gained increasing attention, with inflammation being a crucial component of the MGBA. Studies have shown that dysregulation of the inflammatory response is closely associated with various neurological diseases, such as ASD, AD, and Parkinson’s disease (PD) [49]. This study found that growth-stage exposure to air pollution significantly increased Leukotriene C4 levels in serum, a potent pro-inflammatory mediator, suggesting that air pollution may trigger a systemic inflammatory response. Transcriptomic analysis of brain tissue revealed that exposure activated several inflammation-related signaling pathways, including NOD-like receptor signaling. Metabolomic analysis revealed significant changes in inflammation-related metabolites in the intestine and serum of the exposed group, particularly DHA, alpha-linolenic acid, and gamma-linolenic acid. The decrease in these anti-inflammatory omega-3 fatty acids may weaken immune defense and contribute to the elevated systemic inflammation triggered by air pollution [44,50]. Furthermore, mediation analysis suggests that Leukotriene C4 in the serum mediates the effect of the key differential bacterium *s_Bacteroides_caecimuris* on brain LysoPC levels. Therefore, the disruption of unsaturated fatty acid levels and systemic inflammation induced by air pollution are closely related, potentially leading to brain lipid dysregulation and neuroinflammation, further exacerbating neuronal damage.

This study reveals that exposure to air pollution during the growth stage in mice leads to dysregulation of fatty acid metabolism, abnormal lipid metabolites, and inflammation in multiple organs (such as the intestine and brain), suggesting that environmental factors may affect neurodevelopment by disrupting the MGBA. While fecal microbiome transplantation shows potential for improving neurodevelopmental disorders like autism, precise intervention strategies targeting specific strains or metabolic molecules are still needed. Through multi-omics integration, the study identifies mechanisms by which the gut microbiome, the intestine, and the brain interact through lipid metabolism to regulate neurodevelopment, highlighting their potential as therapeutic targets.

This study has several strengths. First, a whole-body exposure system was used to simulate real-time exposure conditions, offering a more realistic approximation of air pollution exposure. Second, a multi-omics approach was employed to investigate the effects of air pollution exposure during the growth stage on multiple organs and omics in offspring, allowing for a more comprehensive analysis. However, several limitations should be noted. First, as our exposure model simulates real-world conditions with air pollution as a complex mixture, it is challenging to differentiate the effects of individual pollutants, particularly PM_2.5_ and ozone. Second, this study only assessed changes in the microbiome and host metabolites, along with their mediating relationships. Future studies should explore more on the specific microbial strains affecting neurodamage through metabolic pathways. Third, metabolite selection based solely on OPLS-DA VIP scores (VIP > 1) and *p*-values (*p* < 0.05) may increase false-positive risks. Future studies should consider refining the analytical approach to improve the reliability and robustness of results. Fourth, further validation was not conducted using in vitro experiments with cell lines. Future research is encouraged to validate our findings and to shed light on the relevant molecular mechanisms.

## 5. Conclusions

In conclusion, our study demonstrates that growth-stage exposure to air pollution induces neurodamage in mice, potentially via the crosstalk mechanism of the MGBA, and dysregulation of multi-organ lipid metabolism, unsaturated fatty acid metabolism, and systemic inflammation are the potential pathways involved. These findings provide novel insights into the mechanisms underlying growth-stage air pollution-related neuronal damage.

## Figures and Tables

**Table 1:** Alpha diversity indices of gut microbiome.

Characteristics	Control (Mean ± SD)	Exposure (Mean ± SD)	*t*	*p*
Shannon index	6.50 ± 0.42	6.62 ± 0.27	−0.73	0.479
Chao1	33,650.24 ± 18,032.92	34,495.96 ± 16,589.99	−0.11	0.914
Observed species	8378.50 ± 696.35	8805.20 ± 579.11	−1.49	0.154
Simpson index	0.97 ± 0.01	0.96 ± 0.01	0.32	0.754
ACE index	9255.11 ± 890.40	9704.75 ± 569.13	−1.35	0.198

**Table 2:** Mediation analysis of the microbiome and brain lipid-related metabolites.

Outcome Indicator	Exposure	Mediators	Indirect Effect (95% CI)	Proportion Mediated (%)
brain_LysoPC(17:0/0:0)	s__Muribaculaceae_bacterium_Isolate_037_Harlan_	intestinal_Cer(d18:1/16:0)	0.493 (0.106, 1.136)	75.4
	s__Muribaculaceae_bacterium_Isolate_037_Harlan_	serum_Esterase	0.377 (0.083, 0.792)	57.8
	s__Muribaculaceae_bacterium_Isolate_037_Harlan_	serum_PS(18:1(11Z)/20:0)	0.332 (0.015, 0.882)	50.8
	s__Bacteroidales_bacterium	intestinal_Cer(d18:1/16:0)	0.485 (0.073, 1.11)	77.3
	s__Bacteroidales_bacterium	serum_Esterase	0.368 (0.076, 0.763)	58.7
	s__Bacteroidales_bacterium	serum_PS(18:1(11Z)/20:0)	0.343 (0.068, 0.833)	54.6
	s_Bacteroidales_bacterium	serum_gamma-Linolenic acid	0.283 (0.011, 0.736)	45.2
	o__Coriobacteriales	serum_PS(18:1(11Z)/20:0)	−0.102 (−0.23, −0.021)	46.9
	f__Coriobacteriaceae	serum_PS(18:1(11Z)/20:0)	−0.104 (−0.242, −0.022)	46.6
	s__Bacteroidales_bacterium_55_9	serum_Esterase	0.112 (0.004, 0.275)	90.1
	s__Bacteroidales_bacterium_55_9	serum_16-Hydroxyhexadecanoic acid	0.041 (0.003, 0.172)	32.7
	s__Coriobacteriaceae_bacterium	serum_PS(18:1(11Z)/20:0)	−0.064 (−0.189, −0.011)	72.7
	s__Alistipes_sp_58_9_plus	serum_Dimyristoylphosphatidylcholine	0.074 (0.004, 0.148)	66.2
	s__Alistipes_sp_	serum_Leukotriene C4	0.097 (0.007, 0.213)	78.8
	s__Bacteroides_caecimuris	intestinal_Cer(d18:1/16:0)	0.271 (0.042, 0.554)	98.4
	s__Bacteroides_caecimuris	serum_Esterase	0.228 (0.045, 0.576)	83
	s__Bacteroides_caecimuris	intestinal_Ubiquinone-2	0.164 (0.017, 0.395)	59.7
	s__Bacteroides_caecimuris	serum_PS(18:1(11Z)/20:0)	0.216 (0.064, 0.477)	78.4
	s__Bacteroides_caecimuris	serum_Leukotriene C4	0.132 (0.01, 0.326)	48
	s__Bacteroides_caecimuris	serum_gamma-Linolenic acid	0.134 (0.006, 0.415)	48.9
brain_PE(P−16:0/22:6(4Z,7Z,10Z,13Z,16Z,19Z))	f__Rikenellaceae	serum_Arachidoyl Ethanolamide	−0.138 (−0.322, −0.014)	87.6
	f__Rikenellaceae	serum_Heptadecanoic acid	−0.142 (−0.365, −0.018)	89.8
	f__Rikenellaceae	serum_Palmitoylethanolamide	−0.133 (−0.309, −0.028)	84.1
	g_Alistipes	serum_Arachidoyl Ethanolamide	−0.128 (−0.288, −0.003)	94.3
	g_Alistipes	serum_Palmitoylethanolamide	−0.122 (−0.288, −0.01)	90.2
	g__Duncaniella	serum_Phenyllactic acid	0.207 (0.032, 0.48)	51.6
	g__Duncaniella	serum_Sphingosine	0.397 (0.002, 1.091)	98.7
	g_Heminiphilus	serum_Phenyllactic acid	−0.11 (−0.237, −0.01)	52.3
	s__Duncaniella_dubosii	serum_Phenyllactic acid	0.114 (0.008, 0.248)	37.1
	g_Allobaculum	serum_Phenyllactic acid	0.032 (0.005, 0.061)	55.4
	s_Allobaculum_sp_539	serum_Phenyllactic acid	0.032 (0.01, 0.059)	74.1
	s__Alistipes_senegalensis	serum_Arachidoyl Ethanolamide	−0.113 (−0.256, −0.007)	72.9
	s_Alistipes_finegoldii	serum_Arachidoyl Ethanolamide	−0.099 (−0.231, −0.003)	69.5
	s_Alistipes_finegoldii	serum_Heptadecanoic acid	−0.101 (−0.226, −0.002)	70.9
	s__Alistipes_onderdonkii	serum_Arachidoyl Ethanolamide	−0.1 (−0.257, −0.02)	74
	s__Alistipes_onderdonkii	serum_Heptadecanoic acid	−0.103 (−0.238, −0.001)	75.8
	s__Alistipes_shahii	serum_Heptadecanoic acid	−0.147 (−0.329, −0.022)	86.4
	s__Alistipes_timonensis	serum_Palmitoylethanolamide	−0.096 (−0.223, −0.007)	63.7
	s__Alistipes_sp_An66	serum_Arachidoyl Ethanolamide	−0.1 (−0.24, −0.003)	78.1

## Data Availability

The datasets used and/or analyzed during the current study are available from the corresponding author on reasonable request.

## Supplementary Materials

The following supporting information can be downloaded at: https://www.mdpi.com/article/10.3390/toxics13040260/s1. Figure S1: Mass concentration in the ambient air and exposure chambers of PM_2.5_ and ozone; Figure S2: Top 30 VIP scores of brain metabolites; Figure S3: Sex-specific metabolic alterations in the hippocampus and cortex following growth-stage air pollution exposure; Figure S4: Heatmap of gut microbiome abundance; Figure S5: Sex-specific alterations in microbiome structure and function; Figure S6: Top 30 VIP scores of intestinal metabolites; Figure S7: Sex-specific metabolomic and transcriptomic alterations in the intestine; Figure S8: Top 30 VIP scores of serum metabolites; Figure S9: Sex-specific metabolomic alterations in serum; Table S1: The mediation analysis for microbiome and brain metabolites.

## Abbreviations

Acox2: Acyl-CoA Oxidase 2; ADHD, Attention-Deficit/Hyperactivity Disorder; Agt, Angiotensinogen; Aloxe3, Arachidonate Lipoxygenase 3; Angptl4, Angiopoietin-like 4; ASD, Autism Spectrum Disorder; Avpr1a, Arginine Vasopressin Receptor 1A; Brs3, Bombesin-like Receptor 3; C3, Complement Component 3; CA, Cornu Ammonis; Ca, Calcium; Ca^2+^, Calcium Ion; Cer, Ceramide; Chrna6, Cholinergic Receptor Nicotinic Alpha 6 Subunit; CNS, Central Nervous System; Cyp2e1, Cytochrome P450 Family 2 Subfamily E Member 1; Cyp3a13, Cytochrome P450 Family 3 Subfamily A Member 13; Cyp3a25, Cytochrome P450 Family 3 Subfamily A Member 25; DEGs, Differentially Expressed Genes; DHA, Docosahexaenoic Acid; DG, Dentate Gyrus; E, Embryonic Day; Exp, Exposure; FA, Filtered Air; Fe, Iron; FDR, False Discovery Rate; f, Family; Gabrq, Gamma-Aminobutyric Acid Type A Receptor Subunit Theta; g, Genus; Ggt1, Gamma-Glutamyltransferase 1; GPI, Glycosylphosphatidylinositol; Gzma, Granzyme A; HEPA, High-Efficiency Particulate Air; HMDB, Human Metabolome Database; Hmgcs2, 3-Hydroxy-3-Methylglutaryl-CoA Synthase 2; IVCs, Individually Ventilated Cages; KEGG, Kyoto Encyclopedia of Genes and Genomes; LC-MS, Liquid Chromatography-Mass Spectrometry; LDA, Linear Discriminant Analysis; LEfSe, Linear Discriminant Analysis Effect Size; Ltb4r1, Leukotriene B4 Receptor 1; LysoPC, Lysophosphatidylcholine; Me3, Methylation Level 3; MGBA, Microbiome–Gut–Brain Axis; Na, Sodium; NH^4+^, Ammonium Ion; NDDs, Neurodevelopmental Disorders; NMDS, Non-metric Multidimensional Scaling; NO_2_, Nitrogen Dioxide; NOD-like, Nucleotide-binding Oligomerization Domain-like; o, Order; O_3_, Ozone; OPLS-DA, Orthogonal Partial Least Squares Discriminant Analysis; p, Phylum; Pck1, Phosphoenolpyruvate Carboxykinase 1; PC, Phosphatidylcholine; PE, Phosphatidylethanolamine; Pla2g12b, Phospholipase A2 Group XII B; Plin1, Perilipin 1; Plin5, Perilipin 5; PM_2.5_, Fine Particulate Matter; PND, Postnatal Day; PPAR, Peroxisome Proliferator-Activated Receptor; PS, Phosphatidylserine; QC, Quality Control; Q-value, Adjusted *p*-value Controlling for False Discovery Rate; RTAAE, Real-Time Ambient Air Exposure; s, Species; UPLC, Ultra-Performance Liquid Chromatography; VIP score, Variable Importance in Projection Score.
