# Supplementary material for: A Multi-Omics Study of Neurodamage Induced by Growth-Stage Real-Time Air Pollution Exposure in Mice via the Microbiome–Gut–Brain Axis
Source: Toxics. 2025 Mar 29;13(4):260. doi: 10.3390/toxics13040260 (PMC12031392; doi:10.3390/toxics13040260)
Supplement: Supplementary file 1 [file toxics-13-00260-s001.zip › toxics-3516947-supplementary.pdf]

Supplementary Material

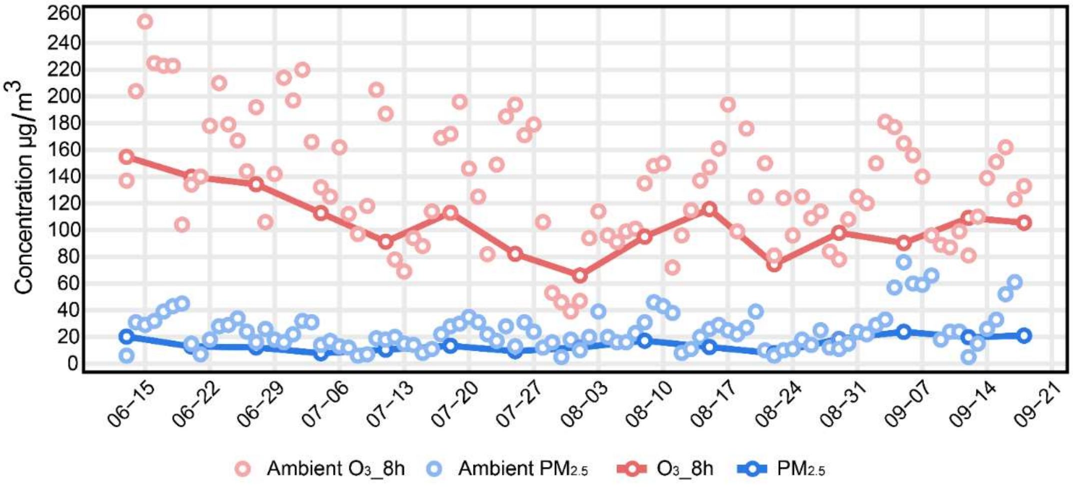

Figure S1. Mass concentration in the ambient air and exposure chambers of $PM_{2.5}$ and ozone.

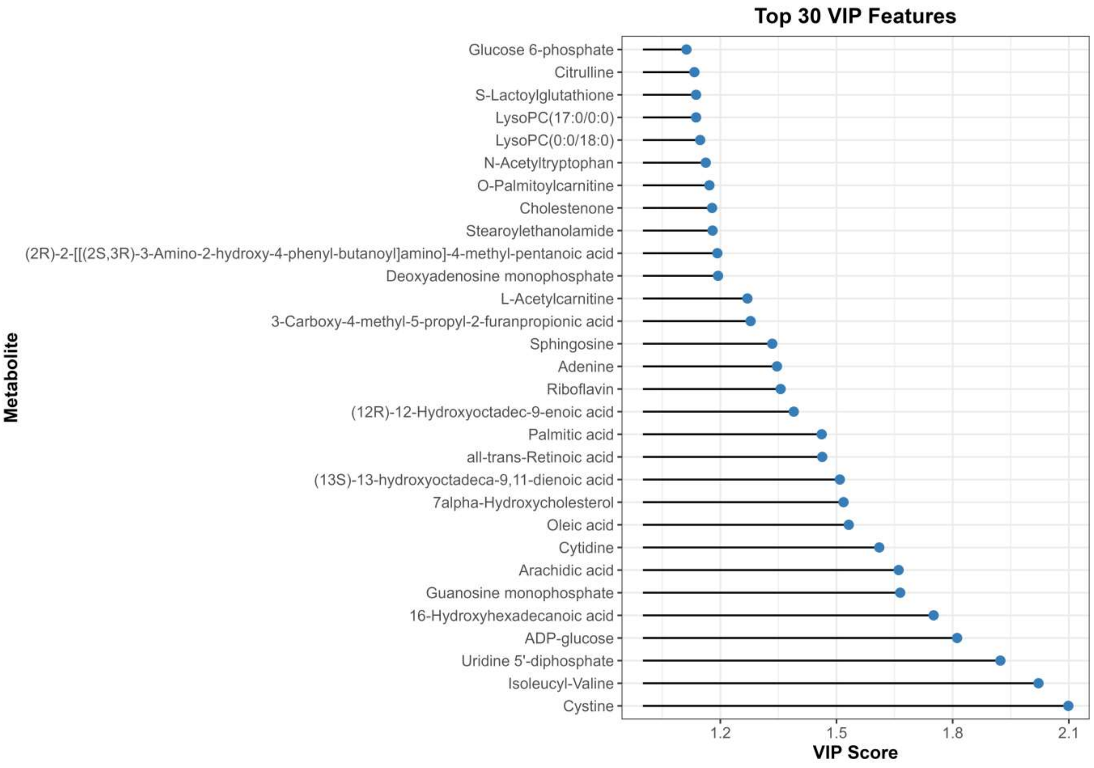

Figure S2. Top 30 VIP scores of brain metabolites

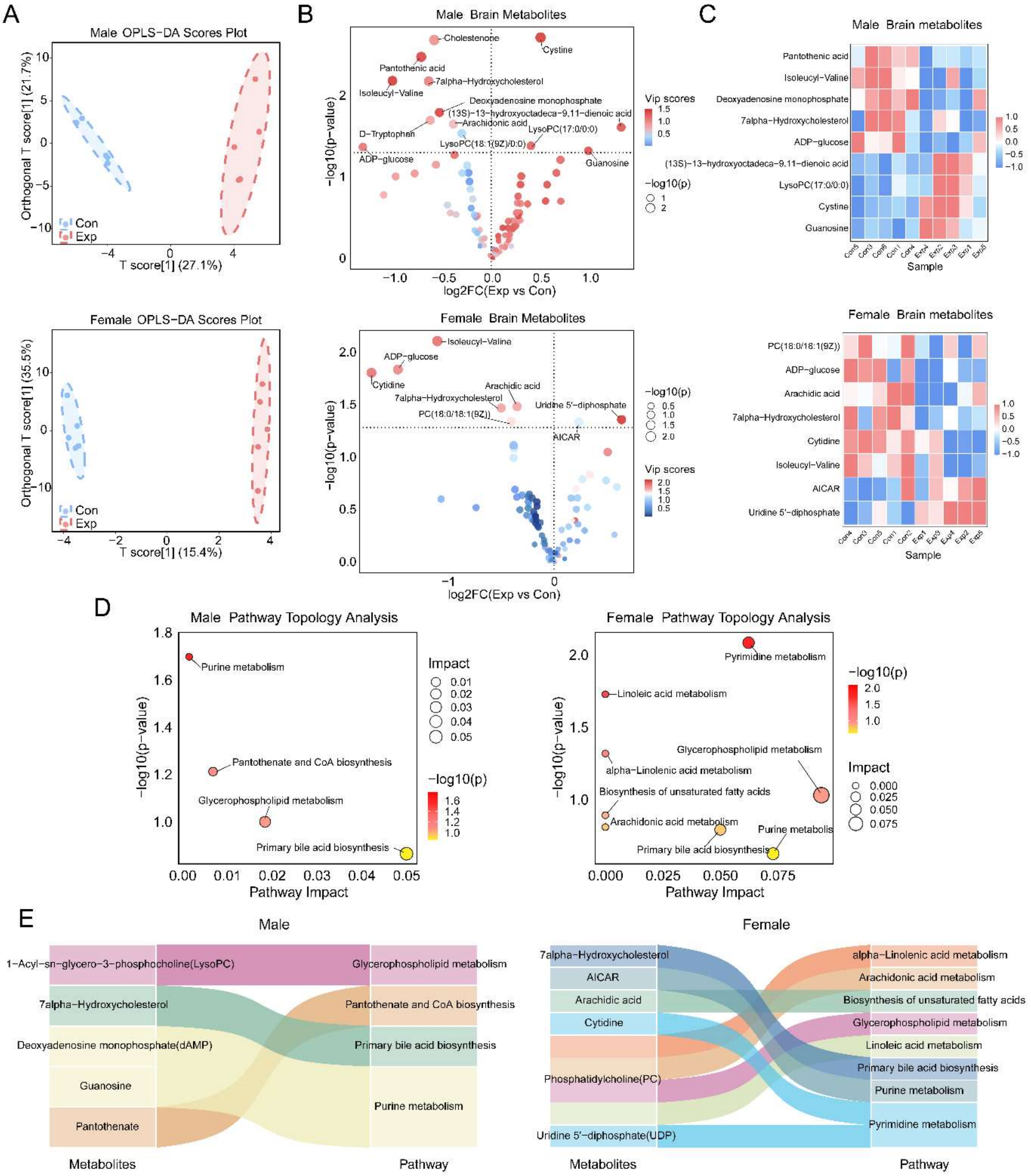

Figure S3. Sex-specific metabolic alterations in the hippocampus and cortex following growth-stage air pollution exposure. (A) OPLS-DA scores plot. (B) Volcano plot of brain metabolite. (C) Heatmap of brain metabolites abundance. (D) Topo-logical analysis of differential metabolites in KEGG pathway. (E) The correlations between differential metabolites and pathways. n = 10 per group (male: 5, female: 5)

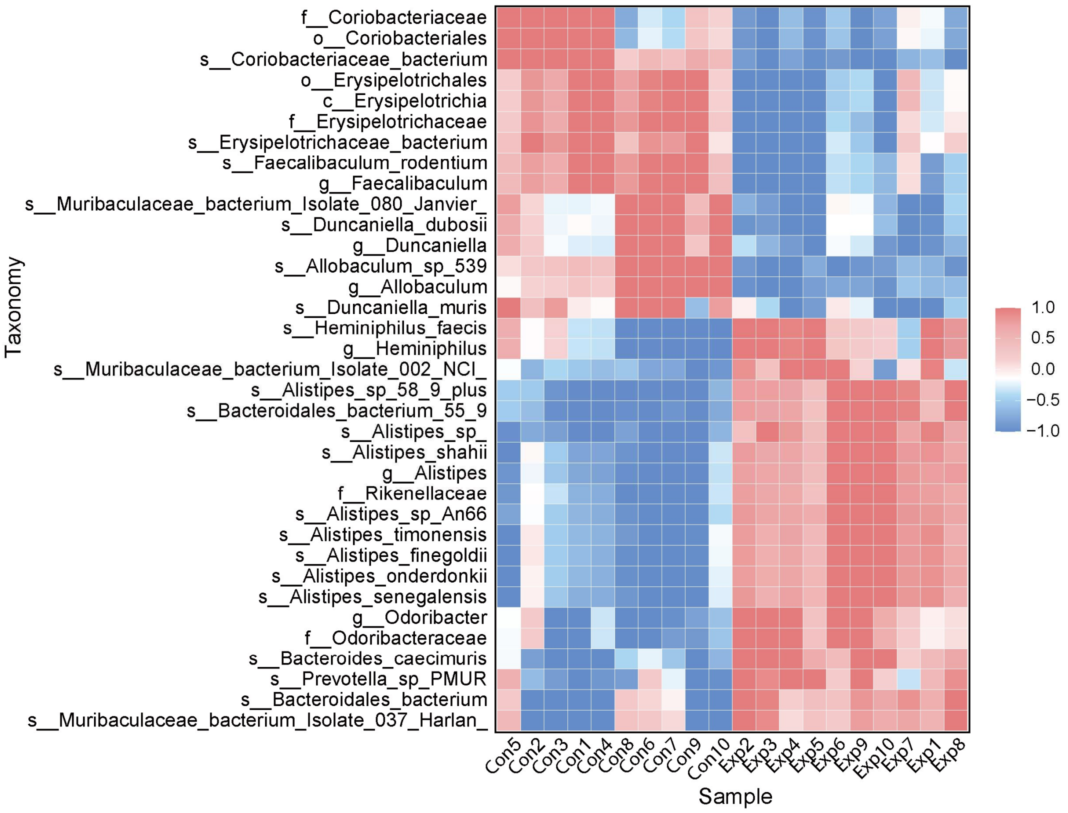

Figure S4. Heatmap of gut microbiome abundance.

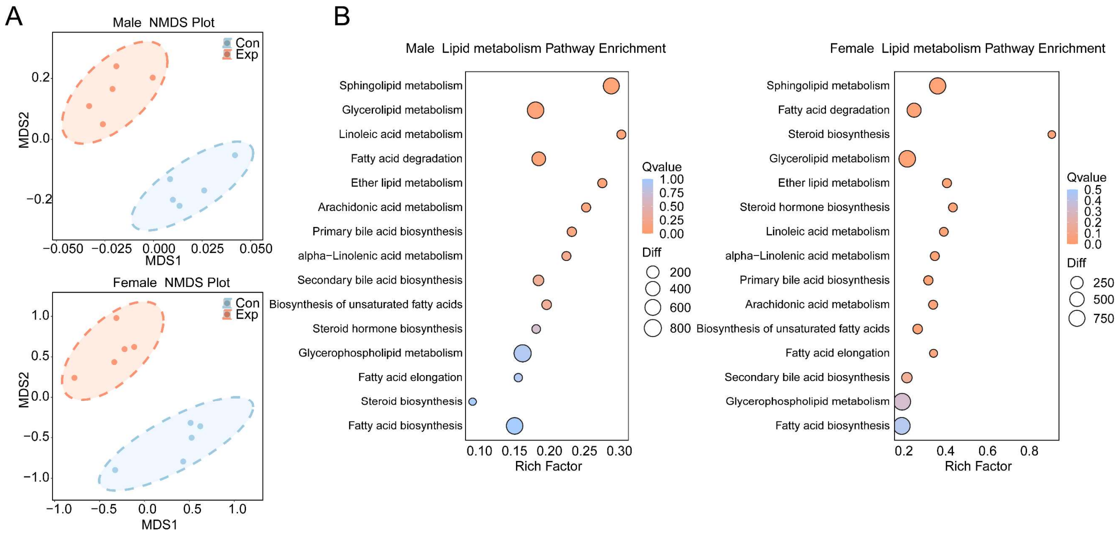

Figure S5. Sex-specific alterations in microbiome structure and function. (A) NMDS plot of gut microbiome. (B) KEGG enrichment of DEGs. the y-axis represents enriched pathways, the x-axis shows the enrichment factor, circle size reflects gene count, and color indicates pathway Q-values. n = 10 per group (male: 5, female: 5)

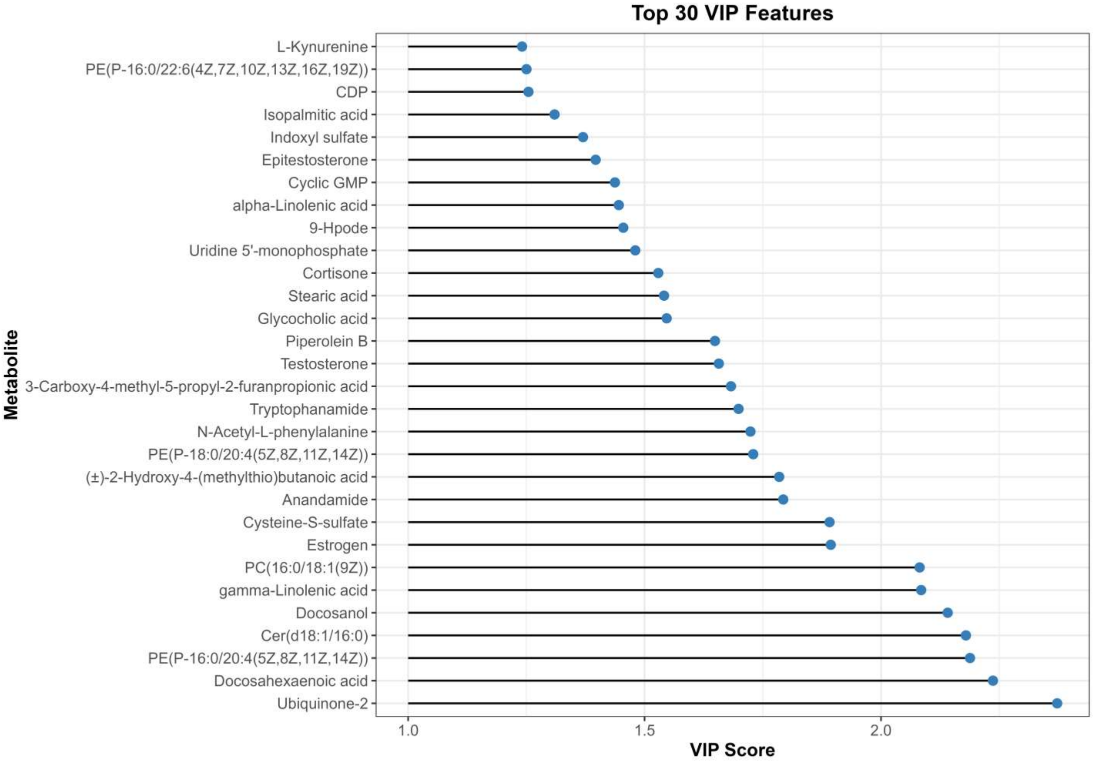

Figure S6. Top 30 VIP scores of intestinal metabolites.

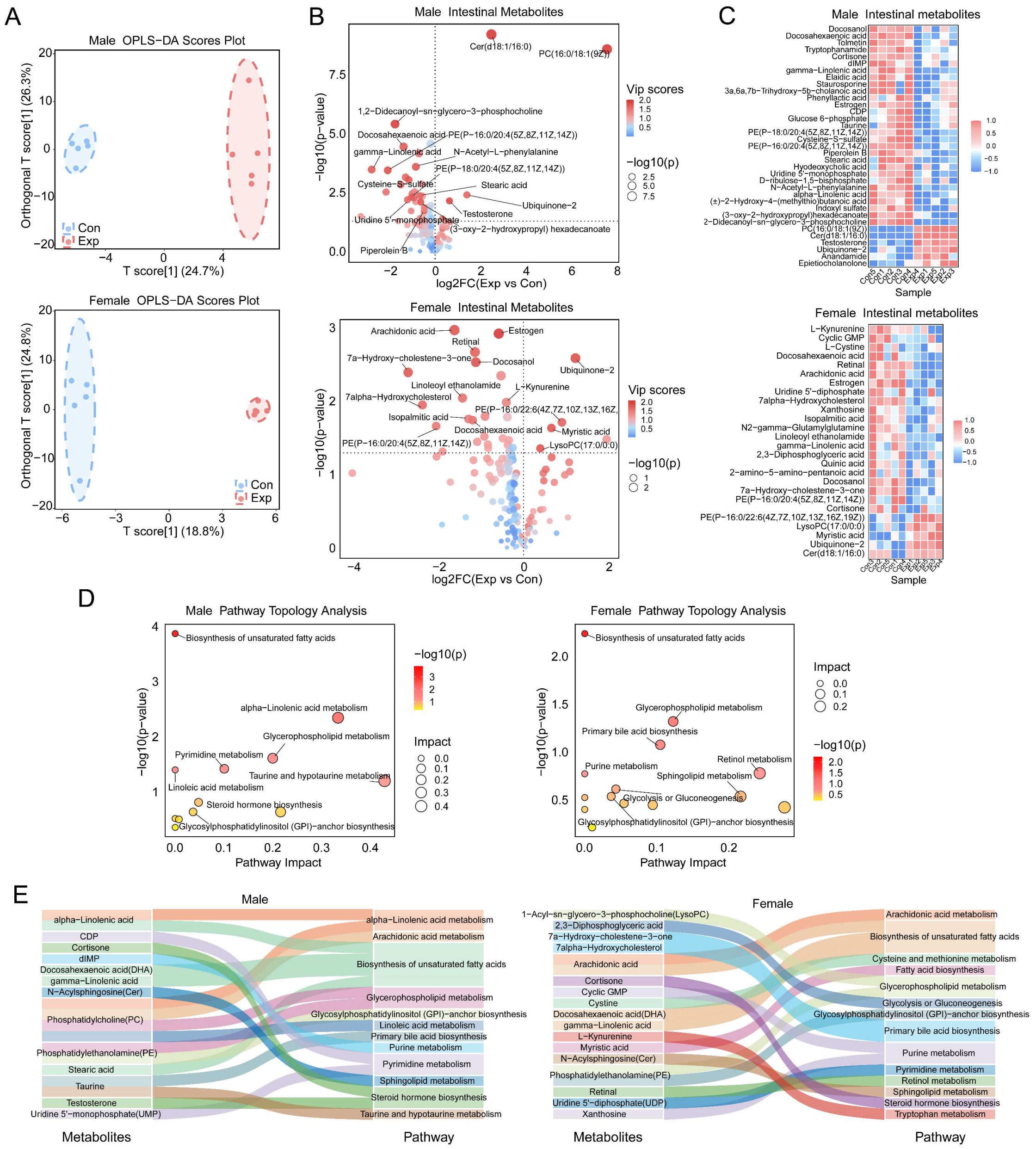

Figure S7. Sex-specific metabolomic and transcriptomic alterations in the Intestine. (A) OPLS-DA scores plot. (B) Volcano plot of intestinal metabolites. (C) Heatmap of intestinal metabolites abundance. (D) Topological analysis of differential metabolites in KEGG pathway. (E) Correlations between differential metabolites and pathways. n = 10 per group (male: 5, female: 5).

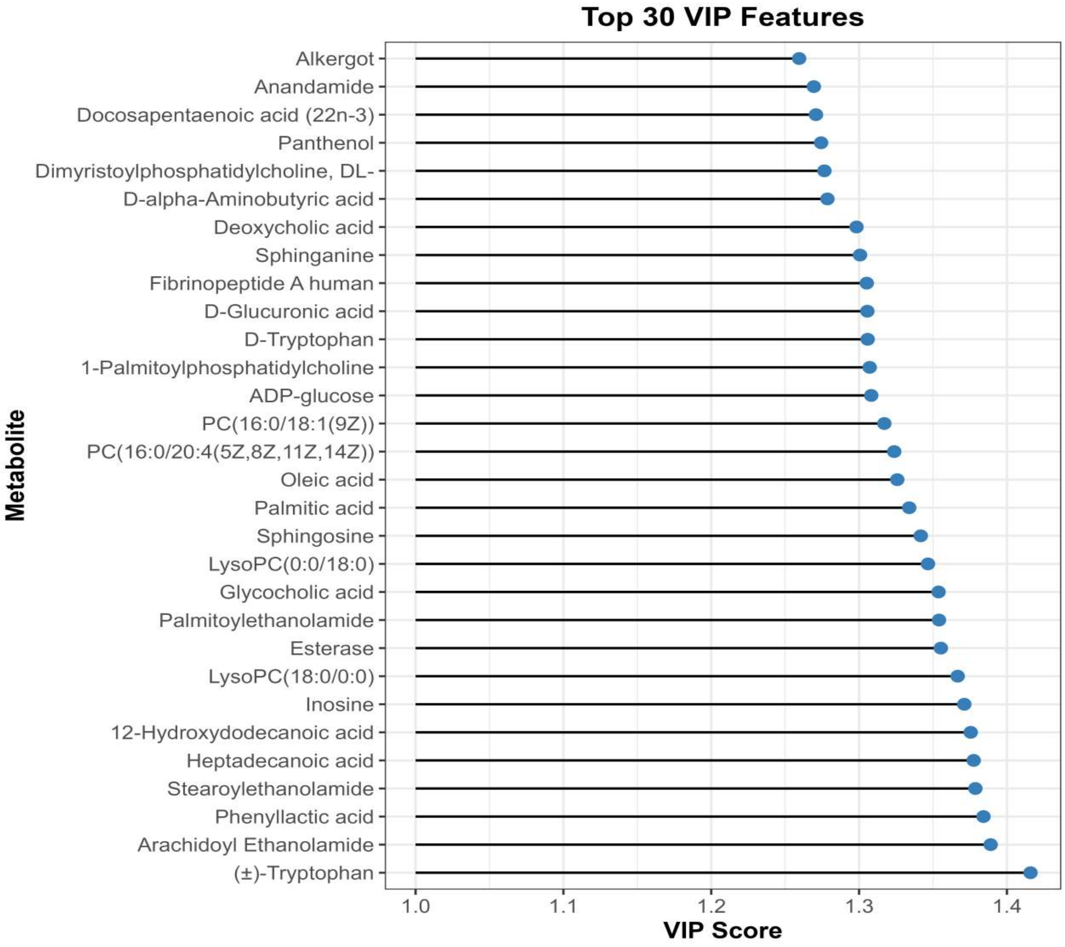

Figure S8. Top 30 VIP scores of serum metabolites.

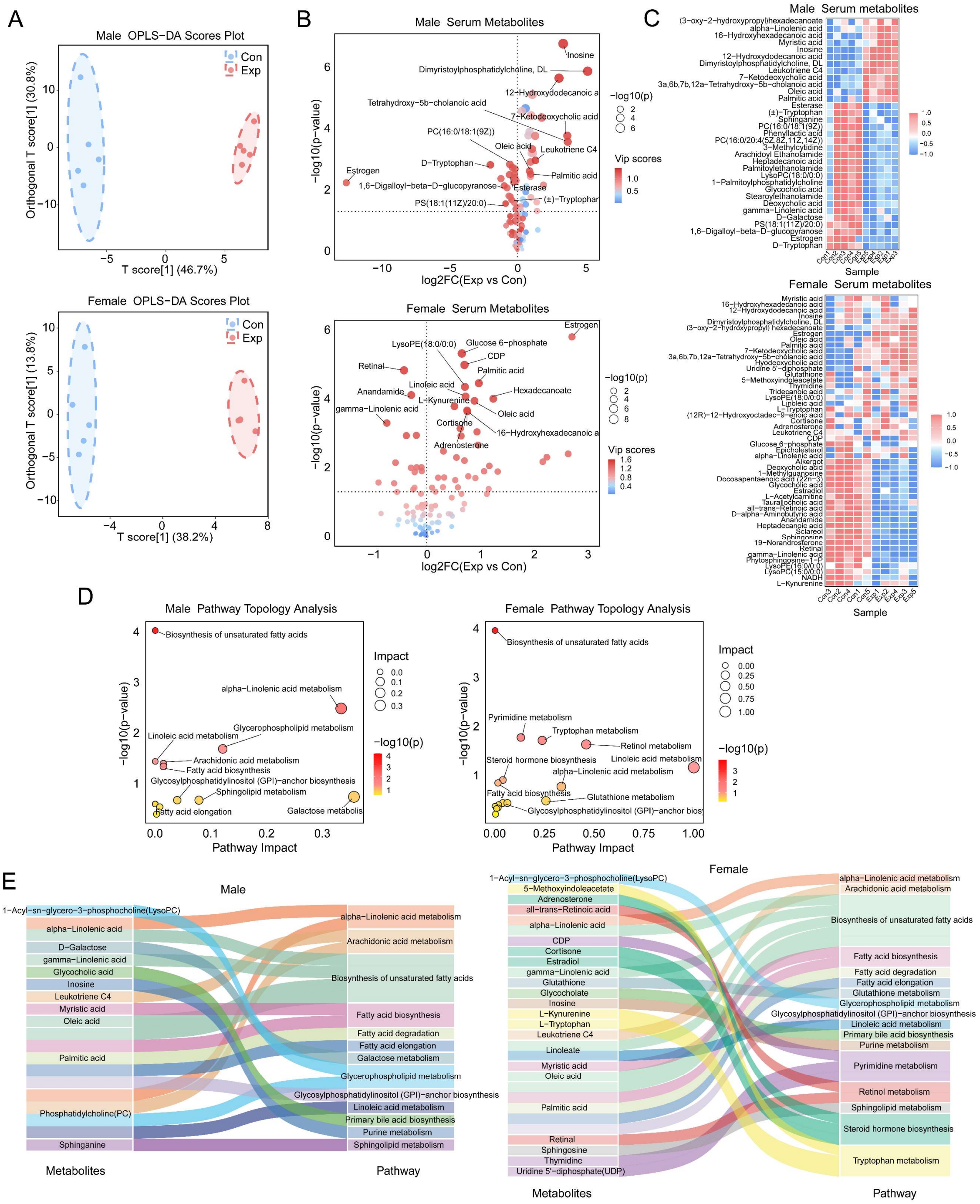

Figure S9. Sex-specific metabolomic alterations in serum. (A) OPLS-DA scores plot. (B) Volcano plot of serum metabolites. (C) Heatmap of serum metabolites abundance. (D) Topological analysis of differential metabolites in KEGG pathway. (E) Correlations between differential metabolites and pathways. n = 10 per group (male: 5, female: 5).

Table S1. The mediation analysis for microbiome and brain metabolites.

Outcome indicator	Exposure	Mediators	Total effects			Indirect effects			Proportion mediated (%)
			Estimate (95% CI)	*P*	P_{FDR}	Estimate (95% CI)	*P*	P_{FDR}	
brain_LysoPC(17:0/0:0)	s_Muribaculaceae_bacterium_Isolate_037_Harlan	intestinal_Cer(d18:1/16:0)	0.653 (0.299, 1.147)	<0.001	<0.001	0.493 (0.106, 1.136)	0.012	0.576	75.4
	s_Muribaculaceae_bacterium_Isolate_037_Harlan	serum_Esterase	0.653 (0.282, 1.142)	0.004	0.008	0.377 (0.083, 0.792)	0.016	0.576	57.8
	s_Muribaculaceae_bacterium_Isolate_037_Harlan	serum_PS(18:1(11Z)/20:0)	0.653 (0.281, 1.103)	<0.001	<0.001	0.332 (0.015, 0.882)	0.044	0.634	50.8
	s_Bacteroidales_bacterium	intestinal_Cer(d18:1/16:0)	0.627 (0.283, 1.026)	<0.001	<0.001	0.485 (0.073, 1.11)	0.028	0.504	77.3
	s_Bacteroidales_bacterium	serum_Esterase	0.627 (0.308, 1.063)	<0.001	<0.001	0.368 (0.076, 0.763)	0.012	0.504	58.7
	s_Bacteroidales_bacterium	serum_PS(18:1(11Z)/20:0)	0.627 (0.301, 1.043)	<0.001	<0.001	0.343 (0.068, 0.833)	0.016	0.504	54.6
	s_Bacteroidales_bacterium	serum_gamma-Linolenic acid	0.627 (0.289, 1.017)	<0.001	<0.001	0.283 (0.011, 0.736)	0.036	0.518	45.2
	o_Coriobacteriales	serum_PS(18:1(11Z)/20:0)	-0.216 (-0.313, -0.105)	<0.001	<0.001	-0.102 (-0.23, -0.021)	0.02	0.480	46.9
	f_Coriobacteriaceae	serum_PS(18:1(11Z)/20:0)	-0.223 (-0.33, -0.129)	<0.001	<0.001	-0.104 (-0.242, -0.022)	0.028	0.672	46.6
	s_Bacteroidales_bacterium_55_9	serum_Esterase	0.124 (0.028, 0.204)	0.024	0.029	0.112 (0.004, 0.275)	0.04	0.535	90.1
	s_Bacteroidales_bacterium_55_9	serum_16-Hydroxyhexadecanoic acid	0.124 (0.024, 0.209)	0.020	0.029	0.041 (0.003, 0.172)	0.028	0.535	32.7
	s_Coriobacteriaceae_bacterium	serum_PS(18:1(11Z)/20:0)	-0.087 (-0.131, -0.035)	0.012	0.012	-0.064 (-0.189, -0.011)	0.008	0.288	72.7
	s_Alistipes_sp_58_9_plus	serum_Dimyristoylphosphatidylcholine, DL	0.112 (0.005, 0.194)	0.032	0.051	0.074 (0.004, 0.148)	0.048	0.494	66.2
	s_Alistipes_sp_	serum_Leukotriene C4	0.123 (0, 0.252)	0.048	0.083	0.097 (0.007, 0.213)	0.032	0.209	78.8
	s_Bacteroides_caecimuris	intestinal_Cer(d18:1/16:0)	0.275 (0.049, 0.468)	0.016	0.025	0.271 (0.042, 0.554)	0.032	0.288	98.4
	s_Bacteroides_caecimuris	intestinal_Ubiquinone-2	0.275 (0.105, 0.455)	<0.001	<0.001	0.164 (0.017, 0.395)	0.016	0.230	59.7
	s_Bacteroides_caecimuris	serum_Esterase	0.275 (0.055, 0.478)	0.028	0.029	0.228 (0.045, 0.576)	0.012	0.216	83

	s_Bacteroides_caecimuris	serum_PS(18:1(11Z)/20:0)	0.275 (0.069, 0.452)	0.016	0.025	0.216 (0.064, 0.477)	<0.001	<0.001	78.4
	s_Bacteroides_caecimuris	serum_Leukotriene C4	0.275 (0.079, 0.432)	0.012	0.025	0.132 (0.01, 0.326)	0.04	0.288	48
	s_Bacteroides_caecimuris	serum_gamma-Linolenic acid	0.275 (0.052, 0.458)	0.020	0.026	0.134 (0.006, 0.415)	0.036	0.288	48.9
brain_PE(P-16:0/22:6(4Z,7Z,10Z,13Z,16Z,19Z))	f_Rikenellaceae	serum_Arachidoyl Ethanolamide	-0.158 (-0.36, -0.016)	0.032	0.070	-0.138 (-0.322, -0.014)	0.036	0.648	87.6
	f_Rikenellaceae	serum_Heptadecanoic acid	-0.158 (-0.409, -0.002)	0.048	0.070	-0.142 (-0.365, -0.018)	0.028	0.648	89.8
	f_Rikenellaceae	serum_Palmitoylethanolamide	-0.158 (-0.414, -0.008)	0.040	0.070	-0.133 (-0.309, -0.028)	0.012	0.432	84.1
	g_Alistipes	serum_Arachidoyl Ethanolamide	-0.136 (-0.357, -0.011)	0.032	0.066	-0.128 (-0.288, -0.003)	0.048	0.672	94.3
	g_Alistipes	serum_Palmitoylethanolamide	-0.136 (-0.366, -0.002)	0.048	0.066	-0.122 (-0.288, -0.01)	0.028	0.672	90.2
	g_Duncaniella	serum_Phenyllactic acid	0.402 (0.04, 0.942)	0.024	0.028	0.207 (0.032, 0.48)	0.012	0.770	51.6
	g_Duncaniella	serum_Sphingosine	0.402 (0.053, 0.984)	0.024	0.028	0.397 (0.002, 1.091)	0.048	0.770	98.7
	g_Heminiphilus	serum_Phenyllactic acid	-0.211 (-0.557, -0.003)	0.048	0.072	-0.11 (-0.237, -0.01)	0.028	0.608	52.3
	s_Duncaniella_dubosii	serum_Phenyllactic acid	0.307 (0.061, 0.729)	0.008	0.024	0.114 (0.008, 0.248)	0.044	0.846	37.1
	g_Allobaculum	serum_Phenyllactic acid	0.058 (0.006, 0.144)	0.024	0.057	0.032 (0.005, 0.061)	0.036	0.846	55.4
	s_Allobaculum_sp_539	serum_Phenyllactic acid	0.043 (0, 0.11)	0.048	0.074	0.032 (0.01, 0.059)	0.016	0.576	74.1
	s_Alistipes_senegalensis	serum_Arachidoyl Ethanolamide	-0.156 (-0.397, -0.01)	0.028	0.067	-0.113 (-0.256, -0.007)	0.04	0.774	72.9
	s_Alistipes_finegoldii	serum_Arachidoyl Ethanolamide	-0.143 (-0.35, -0.007)	0.028	0.050	-0.099 (-0.231, -0.003)	0.036	0.788	69.5
	s_Alistipes_finegoldii	serum_Heptadecanoic acid	-0.143 (-0.369, -0.02)	0.020	0.050	-0.101 (-0.226, -0.002)	0.048	0.788	70.9
	s_Alistipes_onderdonkii	serum_Arachidoyl Ethanolamide	-0.136 (-0.34, -0.009)	0.040	0.053	-0.1 (-0.257, -0.02)	0.02	0.720	74
	s_Alistipes_onderdonkii	serum_Heptadecanoic acid	-0.136 (-0.352, -0.009)	0.036	0.053	-0.103 (-0.238, -0.001)	0.048	0.762	75.8
	s_Alistipes_shahii	serum_Heptadecanoic acid	-0.17 (-0.447, -0.005)	0.048	0.072	-0.147 (-0.329, -0.022)	0.024	0.720	86.4
	s_Alistipes_timonensis	serum_Palmitoylethanolamide	-0.15 (-0.396, -0.003)	0.044	0.055	-0.096 (-0.223, -0.007)	0.048	0.782	63.7
	s_Alistipes_sp_An66	serum_Arachidoyl Ethanolamide	-0.127 (-0.325, -0.011)	0.028	0.059	-0.1 (-0.24, -0.003)	0.044	0.749	78.1

brain_Arachidic acid	f_Erysipelotrichaceae	intestinal_Docosanol	0.043 (0.006, 0.088)	0.008	0.020	0.038 (0.006, 0.095)	0.024	0.060	88.6
	f_Erysipelotrichaceae	intestinal_PC(16:0/18:1(9Z))	0.043 (0.008, 0.083)	0.012	0.020	0.035 (0.009, 0.075)	0.004	0.021	81.2
	f_Erysipelotrichaceae	intestinal_Tryptophanamide	0.043 (0.01, 0.095)	<0.001	<0.001	0.033 (0.002, 0.072)	0.036	0.081	76.9
	f_Erysipelotrichaceae	intestinal_Ubiquinone-2	0.043 (0.008, 0.095)	0.020	0.022	0.04 (0.017, 0.096)	0.004	0.021	93
	f_Erysipelotrichaceae	serum_(±)-Tryptophan	0.043 (0.007, 0.086)	0.012	0.020	0.042 (0.013, 0.11)	<0.001	<0.001	98.5
	f_Erysipelotrichaceae	serum_Esterase	0.043 (0.009, 0.093)	0.016	0.021	0.029 (0.005, 0.067)	0.024	0.060	67.6
	f_Erysipelotrichaceae	serum_Glycocholic acid	0.043 (0.011, 0.089)	0.008	0.020	0.043 (0.014, 0.087)	0.004	0.021	99.6
	f_Erysipelotrichaceae	serum_PC(16:0/20:4(5Z,8Z,11Z,14Z))	0.043 (0.009, 0.093)	0.016	0.021	0.042 (0.018, 0.087)	<0.001	<0.001	97.9
	f_Erysipelotrichaceae	serum_PC(16:0/18:1(9Z))	0.043 (0.009, 0.097)	0.008	0.020	0.03 (0.006, 0.067)	0.02	0.053	70.4
	f_Erysipelotrichaceae	serum_1-Palmitoylphosphatidylcholine	0.043 (0.009, 0.089)	0.020	0.022	0.04 (0.015, 0.088)	0.008	0.026	93.5
	f_Erysipelotrichaceae	serum_Sphinganine	0.043 (0.011, 0.095)	0.008	0.020	0.034 (0.013, 0.084)	<0.001	<0.001	79.3
	f_Erysipelotrichaceae	serum_Deoxycholic acid	0.043 (0.012, 0.095)	<0.001	<0.001	0.042 (0.017, 0.09)	<0.001	<0.001	97.1
	f_Erysipelotrichaceae	serum_Docosapentaenoic acid (22n-3)	0.043 (0.008, 0.089)	0.016	0.021	0.041 (0.012, 0.096)	0.008	0.026	96.1
	f_Erysipelotrichaceae	serum_Alkergot	0.043 (0.009, 0.085)	0.012	0.020	0.036 (0.006, 0.077)	0.008	0.026	83.2
	f_Erysipelotrichaceae	serum_1,6-Digalloyl-beta-D-glucopyranose	0.043 (0.011, 0.086)	0.012	0.020	0.04 (0.014, 0.07)	<0.001	<0.001	92.1
	f_Erysipelotrichaceae	serum_1-Methylguanosine	0.043 (0.006, 0.087)	0.020	0.022	0.036 (0.008, 0.078)	0.012	0.038	83.1
	f_Erysipelotrichaceae	serum_Retinal	0.043 (0.009, 0.088)	0.020	0.022	0.035 (0.011, 0.085)	0.008	0.026	81.9
	f_Erysipelotrichaceae	serum_Phytosphingosine-1-P	0.043 (0.012, 0.087)	0.016	0.021	0.037 (0.011, 0.091)	0.02	0.053	86.1
	f_Erysipelotrichaceae	serum_PS(18:1(11Z)/20:0)	0.043 (0.007, 0.08)	0.012	0.020	0.026 (0.003, 0.064)	0.008	0.026	60.1
	f_Erysipelotrichaceae	serum_LysoPC(17:0/0:0)	0.043 (0.007, 0.088)	0.016	0.021	0.032 (0.01, 0.07)	<0.001	<0.001	73.5
	f_Erysipelotrichaceae	serum_Leukotriene C4	0.043 (0.006, 0.09)	0.012	0.020	0.031 (0.008, 0.069)	0.004	0.021	70.9
	f_Erysipelotrichaceae	serum_gamma-Linolenic acid	0.043 (0.009, 0.092)	0.008	0.020	0.019 (0.001, 0.052)	0.032	0.077	43.8
	c_Erysipelotrichia	intestinal_Cer(d18:1/16:0)	0.045 (0.01, 0.109)	0.016	0.029	0.031 (0, 0.084)	0.044	0.096	69.6

c_Erysipelotrichia	intestinal_Cortisone	0.045 (0.007, 0.11)	0.024	0.029	0.028 (0.009, 0.071)	0.012	0.035	63.6
c_Erysipelotrichia	intestinal_Docosanol	0.045 (0.005, 0.098)	0.024	0.029	0.041 (0.007, 0.101)	0.012	0.035	91.1
c_Erysipelotrichia	intestinal_PC(16:0/18:1(9Z))	0.045 (0.006, 0.091)	0.012	0.029	0.039 (0.013, 0.083)	0.004	0.016	87.2
c_Erysipelotrichia	intestinal_Tryptophanamide	0.045 (0.011, 0.098)	0.004	0.029	0.036 (0.009, 0.074)	0.008	0.025	80.9
c_Erysipelotrichia	intestinal_Ubiquinone-2	0.045 (0.008, 0.102)	0.016	0.029	0.042 (0.016, 0.104)	<0.001	<0.001	94
c_Erysipelotrichia	serum_Esterase	0.045 (0.01, 0.101)	0.012	0.029	0.031 (0.008, 0.071)	0.02	0.051	69.4
c_Erysipelotrichia	serum_Glycocholic acid	0.045 (0.007, 0.098)	0.024	0.029	0.044 (0.014, 0.096)	0.008	0.025	98.9
c_Erysipelotrichia	serum_PC(16:0/20:4(5Z,8Z,11Z,14Z))	0.045 (0.004, 0.107)	0.020	0.029	0.044 (0.017, 0.091)	0.004	0.016	99.4
c_Erysipelotrichia	serum_PC(16:0/18:1(9Z))	0.045 (0.006, 0.093)	0.036	0.036	0.032 (0.008, 0.075)	0.004	0.016	72.2
c_Erysipelotrichia	serum_1-Palmitoylphosphatidylcholine	0.045 (0.007, 0.099)	0.016	0.029	0.042 (0.016, 0.089)	<0.001	<0.001	94.8
c_Erysipelotrichia	serum_Sphinganine	0.045 (0.007, 0.091)	0.016	0.029	0.038 (0.012, 0.08)	<0.001	<0.001	84.1
c_Erysipelotrichia	serum_Deoxycholic acid	0.045 (0.004, 0.098)	0.028	0.031	0.043 (0.015, 0.092)	<0.001	<0.001	96.5
c_Erysipelotrichia	serum_Docosapentaenoic acid (22n-3)	0.045 (0.009, 0.102)	0.008	0.029	0.043 (0.015, 0.097)	0.004	0.016	96.7
c_Erysipelotrichia	serum_Alkergot	0.045 (0.011, 0.094)	0.020	0.029	0.037 (0.013, 0.079)	<0.001	<0.001	82.6
c_Erysipelotrichia	serum_1,6-Digalloyl-beta-D-glucopyranose	0.045 (0.006, 0.099)	0.012	0.029	0.041 (0.014, 0.08)	0.004	0.016	91.1
c_Erysipelotrichia	serum_1-Methylguanosine	0.045 (0.008, 0.093)	0.008	0.029	0.037 (0.006, 0.082)	0.032	0.077	83.8
c_Erysipelotrichia	serum_Retinal	0.045 (0.008, 0.099)	0.024	0.029	0.036 (0.008, 0.086)	0.008	0.025	79.8
c_Erysipelotrichia	serum_Phytosphingosine-1-P	0.045 (0.006, 0.105)	0.032	0.033	0.038 (0.01, 0.088)	0.016	0.043	85.7
c_Erysipelotrichia	serum_PS(18:1(11Z)/20:0)	0.045 (0.008, 0.098)	0.008	0.029	0.028 (0.001, 0.069)	0.048	0.102	62.2
c_Erysipelotrichia	serum_LysoPC(17:0/0:0)	0.045 (0.01, 0.088)	0.012	0.029	0.032 (0.009, 0.07)	0.004	0.016	71.7
c_Erysipelotrichia	serum_Glucosamine 6-phosphate	0.045 (0.008, 0.093)	0.036	0.036	0.023 (0.001, 0.065)	0.04	0.090	52.1
c_Erysipelotrichia	serum_Leukotriene C4	0.045 (0.007, 0.095)	0.024	0.029	0.034 (0.01, 0.069)	0.004	0.016	76.1
o_Erysipelotrichales	intestinal_Cer(d18:1/16:0)	0.045 (0.009, 0.092)	0.020	0.030	0.031 (0.001, 0.086)	0.036	0.076	69.6

o_Erysipelotrichales	intestinal_Cortisone	0.045 (0.007, 0.093)	0.020	0.030	0.028 (0.003, 0.071)	0.048	0.096	63.6
o_Erysipelotrichales	intestinal_Docosanol	0.045 (0.007, 0.102)	0.012	0.030	0.041 (0.007, 0.091)	0.016	0.046	91.1
o_Erysipelotrichales	intestinal_PC(16:0/18:1(9Z))	0.045 (0.003, 0.099)	0.028	0.031	0.039 (0.013, 0.089)	0.004	0.017	87.2
o_Erysipelotrichales	intestinal_Tryptophanamide	0.045 (0.005, 0.097)	0.020	0.030	0.036 (0.007, 0.074)	0.028	0.067	80.9
o_Erysipelotrichales	intestinal_Ubiquinone-2	0.045 (0.007, 0.094)	0.028	0.031	0.042 (0.016, 0.106)	<0.001	<0.001	94
o_Erysipelotrichales	intestinal_(±)-2-Hydroxy-4-(methylthio)butanoic acid	0.045 (0.006, 0.107)	0.020	0.030	0.03 (0.001, 0.085)	0.028	0.067	68
o_Erysipelotrichales	serum_Esterase	0.045 (0.006, 0.098)	0.008	0.030	0.031 (0.006, 0.07)	0.02	0.053	69.4
o_Erysipelotrichales	serum_Glycocholic acid	0.045 (0.009, 0.097)	0.016	0.030	0.044 (0.014, 0.087)	0.012	0.036	98.9
o_Erysipelotrichales	serum_PC(16:0/20:4(5Z,8Z,11Z,14Z))	0.045 (0.007, 0.086)	0.016	0.030	0.044 (0.019, 0.085)	<0.001	<0.001	99.4
o_Erysipelotrichales	serum_PC(16:0/18:1(9Z))	0.045 (0.008, 0.096)	0.016	0.030	0.032 (0.007, 0.075)	0.004	0.017	72.2
o_Erysipelotrichales	serum_1-Palmitoylphosphatidylcholine	0.045 (0.007, 0.103)	0.032	0.033	0.042 (0.015, 0.094)	<0.001	<0.001	94.8
o_Erysipelotrichales	serum_Sphinganine	0.045 (0.009, 0.103)	0.012	0.030	0.038 (0.014, 0.085)	<0.001	<0.001	84.1
o_Erysipelotrichales	serum_Deoxycholic acid	0.045 (0.007, 0.099)	0.032	0.033	0.043 (0.013, 0.094)	<0.001	<0.001	96.5
o_Erysipelotrichales	serum_Docosapentaenoic acid (22n-3)	0.045 (0.006, 0.1)	0.024	0.030	0.043 (0.014, 0.101)	<0.001	<0.001	96.7
o_Erysipelotrichales	serum_Alkergot	0.045 (0.009, 0.096)	0.020	0.030	0.037 (0.012, 0.094)	0.012	0.036	82.6
o_Erysipelotrichales	serum_1,6-Digalloyl-beta-D-glucopyranose	0.045 (0.005, 0.1)	0.024	0.030	0.041 (0.013, 0.079)	0.004	0.017	91.1
o_Erysipelotrichales	serum_1-Methylguanosine	0.045 (0.008, 0.095)	0.020	0.030	0.037 (0.007, 0.077)	0.024	0.062	83.8
o_Erysipelotrichales	serum_Retinal	0.045 (0.007, 0.1)	0.008	0.030	0.036 (0.01, 0.086)	0.004	0.017	79.8
o_Erysipelotrichales	serum_Phytosphingosine-1-P	0.045 (0.007, 0.095)	0.032	0.033	0.038 (0.01, 0.087)	0.02	0.053	85.7
o_Erysipelotrichales	serum_PS(18:1(11Z)/20:0)	0.045 (0.008, 0.097)	0.016	0.030	0.028 (0.004, 0.072)	0.032	0.072	62.2
o_Erysipelotrichales	serum_LysoPC(17:0/0:0)	0.045 (0.01, 0.106)	0.012	0.030	0.032 (0.009, 0.078)	0.008	0.029	71.7
o_Erysipelotrichales	serum_Glucosamine 6-phosphate	0.045 (0.007, 0.097)	0.020	0.030	0.023 (0.001, 0.066)	0.036	0.076	52.1

o_Erysipelotrichales	serum_Leukotriene C4	0.045 (0.012, 0.094)	0.012	0.030	0.034 (0.009, 0.074)	0.004	0.017	76.1
o_Erysipelotrichales	serum_gamma-Linolenic acid	0.045 (0.01, 0.094)	0.016	0.030	0.019 (0.001, 0.054)	0.032	0.072	42.8
s_Erysipelotrichaceae_bacterium	intestinal_PC(16:0/18:1(9Z))	0.045 (0.007, 0.103)	0.024	0.036	0.044 (0.016, 0.108)	0.004	0.034	97.8
s_Erysipelotrichaceae_bacterium	intestinal_Tolmetin	0.045 (0.006, 0.09)	0.020	0.036	0.019 (0.001, 0.044)	0.028	0.078	41.1
s_Erysipelotrichaceae_bacterium	intestinal_Tryptophanamide	0.045 (0.008, 0.096)	0.032	0.037	0.04 (0.001, 0.082)	0.048	0.108	87.2
s_Erysipelotrichaceae_bacterium	intestinal_Ubiquinone-2	0.045 (0.006, 0.104)	0.028	0.037	0.043 (0.02, 0.108)	<0.001	<0.001	95
s_Erysipelotrichaceae_bacterium	serum_(±)-Tryptophan	0.045 (0.01, 0.099)	0.008	0.036	0.044 (0.008, 0.115)	0.008	0.034	96.3
s_Erysipelotrichaceae_bacterium	serum_Esterase	0.045 (0.008, 0.093)	0.024	0.036	0.032 (0.003, 0.076)	0.036	0.089	70.9
s_Erysipelotrichaceae_bacterium	serum_PC(16:0/18:1(9Z))	0.045 (0.011, 0.101)	0.008	0.036	0.033 (0.006, 0.076)	0.012	0.045	73.7
s_Erysipelotrichaceae_bacterium	serum_1-Palmitoylphosphatidylcholine	0.045 (0.008, 0.102)	0.024	0.036	0.043 (0.013, 0.091)	0.008	0.034	93.7
s_Erysipelotrichaceae_bacterium	serum_Sphinganine	0.045 (0.008, 0.093)	0.028	0.037	0.037 (0.011, 0.084)	0.008	0.034	81.2
s_Erysipelotrichaceae_bacterium	serum_Deoxycholic acid	0.045 (0.009, 0.097)	0.012	0.036	0.044 (0.013, 0.098)	0.008	0.034	96.7
s_Erysipelotrichaceae_bacterium	serum_Docosapentaenoic acid (22n-3)	0.045 (0.007, 0.09)	0.024	0.036	0.042 (0.008, 0.1)	0.02	0.065	92.8
s_Erysipelotrichaceae_bacterium	serum_Anandamide	0.045 (0.008, 0.094)	0.008	0.036	0.045 (0.016, 0.109)	0.008	0.034	99.6
s_Erysipelotrichaceae_bacterium	serum_Alkergot	0.045 (0.001, 0.091)	0.044	0.045	0.036 (0.007, 0.087)	0.008	0.034	79.8
s_Erysipelotrichaceae_bacterium	serum_1-Methylguanosine	0.045 (0.005, 0.091)	0.024	0.036	0.037 (0.001, 0.09)	0.036	0.089	81

s_Erysipelotrichaceae_bacterium	serum_Retinal	0.045 (0.005, 0.101)	0.020	0.036	0.035 (0.009, 0.094)	0.008	0.034	76.6
s_Erysipelotrichaceae_bacterium	serum_Phytosphingosine-1-P	0.045 (0.006, 0.092)	0.024	0.036	0.037 (0.01, 0.083)	0.024	0.069	80.8
s_Erysipelotrichaceae_bacterium	serum_PS(18:1(11Z)/20:0)	0.045 (0.003, 0.101)	0.036	0.038	0.028 (0.002, 0.07)	0.024	0.069	61.8
s_Erysipelotrichaceae_bacterium	serum_LysoPC(17:0/0:0)	0.045 (0.006, 0.099)	0.020	0.036	0.034 (0.008, 0.082)	0.004	0.034	75.4
s_Erysipelotrichaceae_bacterium	serum_Leukotriene C4	0.045 (0.009, 0.094)	0.008	0.036	0.04 (0.01, 0.078)	0.004	0.034	87.1
s_Erysipelotrichaceae_bacterium	serum_gamma-Linolenic acid	0.045 (0.007, 0.104)	0.028	0.037	0.022 (0.001, 0.083)	0.04	0.096	48.4
s_Muribaculaceae_bacterium_Isolate_037_Harlan	intestinal_Cer(d18:1/16:0)	-0.127 (-0.278, -0.01)	0.036	0.049	-0.105 (-0.26, -0.005)	0.044	0.113	83
s_Muribaculaceae_bacterium_Isolate_037_Harlan	intestinal_Docosahexaenoic acid	-0.127 (-0.289, -0.018)	0.020	0.049	-0.067 (-0.163, -0.009)	0.024	0.082	53.2
s_Muribaculaceae_bacterium_Isolate_037_Harlan	intestinal_Estrogen	-0.127 (-0.255, -0.009)	0.040	0.049	-0.05 (-0.138, -0.005)	0.028	0.084	39.2
s_Muribaculaceae_bacterium_Isolate_037_Harlan	intestinal_Ubiquinone-2	-0.127 (-0.278, -0.011)	0.024	0.049	-0.093 (-0.294, -0.029)	<0.001	<0.001	73.4
s_Muribaculaceae_bacterium_Isolate_037_Harlan	intestinal_PE(P-16:0/20:4(5Z,8Z,11Z,14Z))	-0.127 (-0.275, -0.014)	0.040	0.049	-0.077 (-0.186, -0.001)	0.044	0.113	60.8
s_Muribaculaceae_bacterium_Isolate_037_Harlan	serum_(±)-Tryptophan	-0.127 (-0.251, -0.019)	0.024	0.049	-0.115 (-0.265, -0.025)	0.008	0.041	91.3
s_Muribaculaceae_bacterium_Isolate_037_Harlan	serum_Arachidoyl Ethanolamide	-0.127 (-0.273, -0.007)	0.036	0.049	-0.103 (-0.215, -0.03)	0.004	0.036	81.5
s_Muribaculaceae_bacterium_Isolate_037_Harlan	serum_Phenyllactic acid	-0.127 (-0.267, -0.008)	0.032	0.049	-0.08 (-0.187, -0.018)	0.004	0.036	63
s_Muribaculaceae_bacterium_Isolate_037_Harlan	serum_Stearoylethanolamide	-0.127 (-0.281, -0.007)	0.044	0.049	-0.114 (-0.221, -0.028)	0.004	0.036	90.1
s_Muribaculaceae_bacterium_Isolate_037_Harlan	serum_Heptadecanoic acid	-0.127 (-0.28, -0.002)	0.048	0.052	-0.108 (-0.234, -0.029)	<0.001	<0.001	85.6

s_Muribaculaceae_bacterium_Isolate_037_Harlan	serum_Esterase	-0.127 (-0.281, -0.012)	0.036	0.049	-0.088 (-0.194, -0.01)	0.036	0.104	69.6
s_Muribaculaceae_bacterium_Isolate_037_Harlan	serum_Palmitoylethanolamide	-0.127 (-0.274, -0.019)	0.020	0.049	-0.101 (-0.215, -0.021)	0.008	0.041	79.9
s_Muribaculaceae_bacterium_Isolate_037_Harlan	serum_Glycocholic acid	-0.127 (-0.274, -0.016)	0.020	0.049	-0.103 (-0.215, -0.032)	0.008	0.041	81.4
s_Muribaculaceae_bacterium_Isolate_037_Harlan	serum_PC(16:0/20:4(5Z,8Z,11Z,14Z))	-0.127 (-0.279, -0.007)	0.036	0.049	-0.105 (-0.236, -0.034)	<0.001	<0.001	83
s_Muribaculaceae_bacterium_Isolate_037_Harlan	serum_PC(16:0/18:1(9Z))	-0.127 (-0.277, -0.005)	0.040	0.049	-0.09 (-0.203, -0.012)	0.008	0.041	71.4
s_Muribaculaceae_bacterium_Isolate_037_Harlan	serum_1-Palmitoylphosphatidylcholine	-0.127 (-0.288, -0.009)	0.036	0.049	-0.094 (-0.205, -0.018)	0.016	0.058	74.4
s_Muribaculaceae_bacterium_Isolate_037_Harlan	serum_Sphinganine	-0.127 (-0.269, -0.013)	0.024	0.049	-0.08 (-0.179, -0.007)	0.04	0.111	63.5
s_Muribaculaceae_bacterium_Isolate_037_Harlan	serum_Deoxycholic acid	-0.127 (-0.278, -0.018)	0.012	0.049	-0.101 (-0.226, -0.019)	0.016	0.058	79.7
s_Muribaculaceae_bacterium_Isolate_037_Harlan	serum_Docosapentaenoic acid (22n-3)	-0.127 (-0.269, -0.014)	0.032	0.049	-0.1 (-0.214, -0.024)	0.012	0.054	79.1
s_Muribaculaceae_bacterium_Isolate_037_Harlan	serum_1,6-Digalloyl-beta-D-glucopyranose	-0.127 (-0.28, -0.018)	0.012	0.049	-0.072 (-0.185, -0.013)	<0.001	<0.001	56.7
s_Muribaculaceae_bacterium_Isolate_037_Harlan	serum_1-Methylguanosine	-0.127 (-0.285, -0.01)	0.036	0.049	-0.085 (-0.195, -0.02)	0.008	0.041	67.2
s_Muribaculaceae_bacterium_Isolate_037_Harlan	serum_Retinal	-0.127 (-0.27, -0.01)	0.032	0.049	-0.118 (-0.239, -0.033)	0.004	0.036	93.5
s_Muribaculaceae_bacterium_Isolate_037_Harlan	serum_Phytosphingosine-1-P	-0.127 (-0.271, -0.019)	0.020	0.049	-0.06 (-0.142, -0.004)	0.028	0.084	47.7
s_Muribaculaceae_bacterium_Isolate_037_Harlan	serum_Leukotriene C4	-0.127 (-0.264, -0.013)	0.028	0.049	-0.091 (-0.221, -0.015)	0.016	0.058	71.8
s_Muribaculaceae_bacterium_Isolate_037_Harlan	serum_gamma-Linolenic acid	-0.127 (-0.28, -0.011)	0.028	0.049	-0.096 (-0.247, -0.008)	0.028	0.084	75.6
f_Rikenellaceae	intestinal_Cortisone	-0.075 (-0.122, -0.032)	<0.001	<0.001	-0.017 (-0.051, -0.006)	0.008	0.192	23.1

f_Rikenellaceae	intestinal_Tryptophanamide	-0.075 (-0.116, -0.029)	0.004	0.004	-0.022 (-0.057, -0.002)	0.02	0.247	28.8
f_Rikenellaceae	serum_PC(16:0/20:4(5Z,8Z,11Z,14Z))	-0.075 (-0.117, -0.029)	<0.001	<0.001	-0.044 (-0.094, -0.004)	0.036	0.264	58.3
f_Rikenellaceae	serum_PC(16:0/18:1(9Z))	-0.075 (-0.117, -0.033)	<0.001	<0.001	-0.029 (-0.078, -0.001)	0.044	0.264	38.7
f_Rikenellaceae	serum_Sphinganine	-0.075 (-0.119, -0.031)	0.004	0.004	-0.039 (-0.072, -0.004)	0.032	0.264	51.3
f_Rikenellaceae	serum_Anandamide	-0.075 (-0.12, -0.034)	<0.001	<0.001	-0.055 (-0.107, -0.018)	0.008	0.192	73.1
f_Rikenellaceae	serum_Retinal	-0.075 (-0.117, -0.031)	0.004	0.004	-0.053 (-0.1, -0.01)	0.02	0.247	70.4
f_Rikenellaceae	serum_Phytosphingosine-1-P	-0.075 (-0.125, -0.032)	<0.001	<0.001	-0.05 (-0.112, -0.001)	0.044	0.264	66.6
f_Rikenellaceae	serum_LysoPC(17:0/0:0)	-0.075 (-0.125, -0.039)	<0.001	<0.001	-0.038 (-0.082, -0.012)	0.008	0.192	50.4
f_Rikenellaceae	serum_Leukotriene C4	-0.075 (-0.123, -0.03)	<0.001	<0.001	-0.031 (-0.073, -0.006)	0.024	0.247	40.8
f_Rikenellaceae	serum_gamma-Linolenic acid	-0.075 (-0.119, -0.029)	0.004	0.004	-0.027 (-0.073, -0.002)	0.024	0.247	35.9
g_Alistipes	intestinal_Cortisone	-0.067 (-0.103, -0.027)	<0.001	<0.001	-0.015 (-0.049, -0.004)	0.004	0.288	21.7
g_Alistipes	intestinal_Tryptophanamide	-0.067 (-0.109, -0.031)	<0.001	<0.001	-0.018 (-0.046, -0.002)	0.028	0.317	27.4
g_Alistipes	serum_PC(16:0/20:4(5Z,8Z,11Z,14Z))	-0.067 (-0.106, -0.028)	<0.001	<0.001	-0.036 (-0.078, -0.001)	0.044	0.317	54.4
g_Alistipes	serum_Sphinganine	-0.067 (-0.104, -0.03)	<0.001	<0.001	-0.032 (-0.06, -0.005)	0.032	0.317	47.8
g_Alistipes	serum_Anandamide	-0.067 (-0.105, -0.03)	0.004	0.004	-0.045 (-0.079, -0.001)	0.044	0.317	67.8
g_Alistipes	serum_LysoPC(17:0/0:0)	-0.067 (-0.105, -0.026)	<0.001	<0.001	-0.032 (-0.061, -0.006)	0.028	0.317	48
g_Alistipes	serum_Leukotriene C4	-0.067 (-0.1, -0.029)	<0.001	<0.001	-0.027 (-0.065, -0.003)	0.02	0.317	40
g_Alistipes	serum_gamma-Linolenic acid	-0.067 (-0.107, -0.029)	<0.001	<0.001	-0.024 (-0.061, -0.002)	0.028	0.317	35.1
s_Prevotella_sp_PMUR	intestinal_Cortisone	-0.039 (-0.096, -0.006)	0.028	0.065	-0.023 (-0.058, -0.002)	0.036	0.096	59.5
s_Prevotella_sp_PMUR	intestinal_Docosahexaenoic acid	-0.039 (-0.091, -0.002)	0.044	0.065	-0.035 (-0.088, -0.007)	0.008	0.034	88.3
s_Prevotella_sp_PMUR	intestinal_Docosanol	-0.039 (-0.098, -0.005)	0.020	0.065	-0.027 (-0.062, -0.005)	0.02	0.060	69.1
s_Prevotella_sp_PMUR	intestinal_PE(P-16:0/20:4(5Z,8Z,11Z,14Z))	-0.039 (-0.102, -0.003)	0.040	0.065	-0.029 (-0.065, -0.001)	0.044	0.106	74.1
s_Prevotella_sp_PMUR	serum_Esterase	-0.039 (-0.094, 0)	0.048	0.065	-0.03 (-0.066, -0.007)	0.008	0.034	75.8

s_Prevotella_sp_PMUR	serum_Palmitoylethanolamide	-0.039 (-0.098, -0.002)	0.044	0.065	-0.038 (-0.082, -0.012)	<0.001	<0.001	97.6
s_Prevotella_sp_PMUR	serum_PC(16:0/20:4(5Z,8Z,11Z,14Z))	-0.039 (-0.091, 0)	0.048	0.065	-0.034 (-0.074, -0.008)	0.016	0.052	87.3
s_Prevotella_sp_PMUR	serum_PC(16:0/18:1(9Z))	-0.039 (-0.097, -0.001)	0.048	0.065	-0.029 (-0.072, -0.005)	<0.001	<0.001	73.4
s_Prevotella_sp_PMUR	serum_1-Palmitoylphosphatidylcholine	-0.039 (-0.097, -0.001)	0.048	0.065	-0.034 (-0.067, -0.01)	<0.001	<0.001	85.6
s_Prevotella_sp_PMUR	serum_Docosapentaenoic acid (22n-3)	-0.039 (-0.108, -0.001)	0.044	0.065	-0.037 (-0.075, -0.009)	0.008	0.034	93.2
s_Prevotella_sp_PMUR	serum_Anandamide	-0.039 (-0.098, -0.001)	0.048	0.065	-0.039 (-0.087, -0.012)	0.008	0.034	98.4
s_Prevotella_sp_PMUR	serum_Alkergot	-0.039 (-0.097, -0.002)	0.040	0.065	-0.033 (-0.069, -0.006)	0.02	0.060	83.6
s_Prevotella_sp_PMUR	serum_Retinal	-0.039 (-0.098, -0.003)	0.036	0.065	-0.033 (-0.08, -0.009)	0.016	0.052	83.6
s_Prevotella_sp_PMUR	serum_LysoPC(17:0/0:0)	-0.039 (-0.098, -0.001)	0.040	0.065	-0.027 (-0.068, -0.002)	0.032	0.089	68.1
s_Prevotella_sp_PMUR	serum_Glucosamine 6-phosphate	-0.039 (-0.1, -0.003)	0.044	0.065	-0.023 (-0.055, -0.001)	0.04	0.103	58.2
g_Duncaniella	intestinal_Cortisone	0.081 (0.003, 0.142)	0.044	0.062	0.044 (0.017, 0.119)	<0.001	<0.001	54.4
g_Duncaniella	intestinal_Estrogen	0.081 (0.002, 0.141)	0.048	0.062	0.049 (0.009, 0.122)	0.004	0.029	60.3
g_Duncaniella	serum_(±)-Tryptophan	0.081 (0.002, 0.142)	0.044	0.062	0.074 (0.02, 0.181)	0.012	0.041	91.3
g_Duncaniella	serum_PC(16:0/18:1(9Z))	0.081 (0.007, 0.144)	0.028	0.062	0.06 (0.011, 0.206)	0.016	0.048	74.2
g_Duncaniella	serum_1,6-Digalloyl-beta-D-glucopyranose	0.081 (0.007, 0.152)	0.028	0.062	0.068 (0.016, 0.194)	0.012	0.041	84.3
g_Duncaniella	serum_1-Methylguanosine	0.081 (0.009, 0.138)	0.028	0.062	0.079 (0.02, 0.173)	0.016	0.048	98.2
g_Duncaniella	serum_LysoPC(17:0/0:0)	0.081 (0.005, 0.143)	0.036	0.062	0.074 (0.021, 0.185)	0.012	0.041	91.5
g_Duncaniella	serum_Glucosamine 6-phosphate	0.081 (0.001, 0.14)	0.048	0.062	0.075 (0.009, 0.17)	0.036	0.096	92.7
g_Heminiphilus	intestinal_Lauroyl diethanolamide	-0.039 (-0.099, -0.001)	0.044	0.107	-0.025 (-0.063, -0.002)	0.036	0.096	64.5
s_Heminiphilus_faecis	intestinal_Tryptophanamide	-0.039 (-0.112, -0.001)	0.044	0.110	-0.028 (-0.065, -0.007)	0.02	0.063	73.5
s_Heminiphilus_faecis	serum_Esterase	-0.039 (-0.097, -0.002)	0.032	0.110	-0.028 (-0.072, -0.008)	0.02	0.063	72.9

s_Heminiphilus_faecis	serum_1,6-Digalloyl-beta-D-glucopyranose	-0.039 (-0.098, -0.002)	0.036	0.110	-0.026 (-0.065, -0.004)	0.02	0.063	66.8
s_Duncaniella_dubosii	intestinal_Cortisone	0.053 (0.005, 0.089)	0.032	0.060	0.033 (0.012, 0.088)	0.004	0.026	63.4
s_Duncaniella_dubosii	intestinal_PC(16:0/18:1(9Z))	0.053 (0.001, 0.09)	0.044	0.060	0.032 (0.002, 0.087)	0.032	0.089	61.2
s_Duncaniella_dubosii	intestinal_Tryptophanamide	0.053 (0.005, 0.088)	0.020	0.060	0.029 (0.003, 0.082)	0.032	0.089	56
s_Duncaniella_dubosii	serum_Esterase	0.053 (0.008, 0.095)	0.028	0.060	0.041 (0.001, 0.128)	0.044	0.109	78.8
s_Duncaniella_dubosii	serum_PC(16:0/18:1(9Z))	0.053 (0.003, 0.1)	0.040	0.060	0.038 (0.004, 0.105)	0.028	0.084	71.4
s_Duncaniella_dubosii	serum_Sphinganine	0.053 (0.001, 0.09)	0.048	0.060	0.051 (0.016, 0.106)	<0.001	<0.001	97.8
s_Duncaniella_dubosii	serum_1-Methylguanosine	0.053 (0.004, 0.092)	0.044	0.060	0.05 (0.015, 0.123)	0.012	0.045	95.2
s_Duncaniella_dubosii	serum_LysoPC(17:0/0:0)	0.053 (0.005, 0.097)	0.032	0.060	0.043 (0.014, 0.096)	<0.001	<0.001	81.8
s_Duncaniella_dubosii	serum_Glucosamine 6-phosphate	0.053 (0, 0.095)	0.044	0.060	0.044 (0.002, 0.105)	0.04	0.103	84
g_Faecalibaculum	intestinal_Cortisone	0.028 (0.005, 0.052)	0.020	0.020	0.014 (0.003, 0.038)	0.02	0.060	51.1
g_Faecalibaculum	intestinal_Docosahexaenoic acid	0.028 (0.007, 0.055)	0.020	0.020	0.026 (0.007, 0.059)	0.008	0.034	94.9
g_Faecalibaculum	intestinal_Docosanol	0.028 (0.009, 0.052)	<0.001	<0.001	0.018 (0.004, 0.045)	0.016	0.052	66.1
g_Faecalibaculum	intestinal_PC(16:0/18:1(9Z))	0.028 (0.007, 0.053)	0.012	0.014	0.021 (0.007, 0.045)	<0.001	<0.001	76.9
g_Faecalibaculum	intestinal_Tryptophanamide	0.028 (0.008, 0.056)	0.004	0.008	0.019 (0.002, 0.041)	0.024	0.066	66.9
g_Faecalibaculum	intestinal_Ubiquinone-2	0.028 (0.008, 0.053)	<0.001	<0.001	0.025 (0.011, 0.058)	<0.001	<0.001	90.7
g_Faecalibaculum	serum_(±)-Tryptophan	0.028 (0.008, 0.051)	0.004	0.008	0.027 (0.003, 0.063)	0.024	0.066	97.7
g_Faecalibaculum	serum_Phenyllactic acid	0.028 (0.008, 0.054)	0.008	0.010	0.024 (0.003, 0.049)	0.032	0.079	86.7
g_Faecalibaculum	serum_Heptadecanoic acid	0.028 (0.008, 0.054)	<0.001	<0.001	0.027 (0.008, 0.054)	<0.001	<0.001	97.3
g_Faecalibaculum	serum_Esterase	0.028 (0.007, 0.052)	<0.001	<0.001	0.016 (0.001, 0.039)	0.036	0.084	59.1
g_Faecalibaculum	serum_Palmitoylethanolamide	0.028 (0.007, 0.056)	0.008	0.010	0.026 (0.007, 0.051)	0.008	0.034	94.3
g_Faecalibaculum	serum_Glycocholic acid	0.028 (0.007, 0.055)	0.008	0.010	0.023 (0.005, 0.047)	0.008	0.034	84
g_Faecalibaculum	serum_PC(16:0/20:4(5Z,8Z,11Z,14Z))	0.028 (0.007, 0.055)	<0.001	<0.001	0.025 (0.008, 0.048)	<0.001	<0.001	88.9

g_Faecalibaculum	serum_PC(16:0/18:1(9Z))	0.028 (0.008, 0.057)	0.008	0.010	0.018 (0.003, 0.04)	0.016	0.052	63.2
g_Faecalibaculum	serum_1-Palmitoylphosphatidylcholine	0.028 (0.006, 0.053)	0.008	0.010	0.022 (0.007, 0.043)	0.004	0.022	80.5
g_Faecalibaculum	serum_Sphinganine	0.028 (0.008, 0.055)	<0.001	<0.001	0.022 (0.008, 0.055)	<0.001	<0.001	78.3
g_Faecalibaculum	serum_Deoxycholic acid	0.028 (0.008, 0.053)	0.004	0.008	0.023 (0.005, 0.047)	0.012	0.043	82.5
g_Faecalibaculum	serum_Docosapentaenoic acid (22n-3)	0.028 (0.009, 0.058)	<0.001	<0.001	0.023 (0.004, 0.051)	0.004	0.022	82.2
g_Faecalibaculum	serum_Anandamide	0.028 (0.008, 0.056)	0.008	0.010	0.025 (0.008, 0.052)	0.004	0.022	90.3
g_Faecalibaculum	serum_Alkergot	0.028 (0.008, 0.054)	0.004	0.008	0.021 (0.004, 0.047)	0.028	0.075	73.7
g_Faecalibaculum	serum_1,6-Digalloyl-beta-D-glucopyranose	0.028 (0.007, 0.054)	0.008	0.010	0.021 (0.005, 0.045)	0.02	0.060	76.4
g_Faecalibaculum	serum_Retinal	0.028 (0.006, 0.054)	0.008	0.010	0.021 (0.006, 0.047)	0.012	0.043	74.4
g_Faecalibaculum	serum_Phytosphingosine-1-P	0.028 (0.008, 0.057)	0.004	0.008	0.02 (0.005, 0.051)	0.004	0.022	73.4
g_Faecalibaculum	serum_PS(18:1(11Z)/20:0)	0.028 (0.006, 0.055)	0.016	0.017	0.015 (0.001, 0.046)	0.036	0.084	54.8
g_Faecalibaculum	serum_LysoPC(17:0/0:0)	0.028 (0.008, 0.053)	0.008	0.010	0.017 (0.005, 0.036)	0.004	0.022	62.6
g_Faecalibaculum	serum_Glucosamine 6-phosphate	0.028 (0.005, 0.054)	0.004	0.008	0.013 (0.001, 0.034)	0.04	0.090	45.9
g_Faecalibaculum	serum_Leukotriene C4	0.028 (0.006, 0.055)	0.008	0.010	0.018 (0.004, 0.046)	<0.001	<0.001	63.1
g_Faecalibaculum	serum_gamma-Linolenic acid	0.028 (0.008, 0.055)	0.008	0.010	0.01 (0, 0.031)	0.048	0.105	36.9
s_Faecalibaculum_rodentium	intestinal_Cortisone	0.028 (0.008, 0.055)	0.008	0.013	0.014 (0.003, 0.041)	0.032	0.074	51.1
s_Faecalibaculum_rodentium	intestinal_Docosahexaenoic acid	0.028 (0.005, 0.055)	0.012	0.014	0.026 (0.008, 0.058)	0.012	0.043	94.9
s_Faecalibaculum_rodentium	intestinal_Docosanol	0.028 (0.008, 0.057)	0.004	0.010	0.018 (0.001, 0.048)	0.032	0.074	66.1
s_Faecalibaculum_rodentium	intestinal_PC(16:0/18:1(9Z))	0.028 (0.007, 0.057)	0.012	0.014	0.021 (0.007, 0.046)	0.004	0.024	76.9
s_Faecalibaculum_rodentium	intestinal_Tryptophanamide	0.028 (0.006, 0.058)	0.004	0.010	0.019 (0.004, 0.039)	0.016	0.052	66.9

s_Faecalibaculum_rodentium	intestinal_Ubiquinone-2	0.028 (0.005, 0.055)	0.016	0.017	0.025 (0.01, 0.054)	<0.001	<0.001	90.7
s_Faecalibaculum_rodentium	serum_(±)-Tryptophan	0.028 (0.006, 0.054)	0.004	0.010	0.027 (0.004, 0.062)	0.024	0.066	97.7
s_Faecalibaculum_rodentium	serum_Phenyllactic acid	0.028 (0.008, 0.056)	0.008	0.013	0.024 (0.005, 0.05)	0.02	0.058	86.7
s_Faecalibaculum_rodentium	serum_Heptadecanoic acid	0.028 (0.006, 0.057)	0.008	0.013	0.027 (0.009, 0.051)	0.008	0.034	97.3
s_Faecalibaculum_rodentium	serum_Palmitoylethanolamide	0.028 (0.008, 0.054)	0.004	0.010	0.026 (0.008, 0.052)	0.004	0.024	94.3
s_Faecalibaculum_rodentium	serum_Glycocholic acid	0.028 (0.005, 0.054)	0.020	0.020	0.023 (0.007, 0.048)	0.004	0.024	84
s_Faecalibaculum_rodentium	serum_PC(16:0/20:4(5Z,8Z,11Z,14Z))	0.028 (0.008, 0.057)	0.004	0.010	0.025 (0.01, 0.048)	0.004	0.024	88.9
s_Faecalibaculum_rodentium	serum_PC(16:0/18:1(9Z))	0.028 (0.005, 0.056)	0.012	0.014	0.018 (0.004, 0.04)	<0.001	<0.001	63.2
s_Faecalibaculum_rodentium	serum_1-Palmitoylphosphatidylcholine	0.028 (0.008, 0.062)	0.004	0.010	0.022 (0.007, 0.047)	0.004	0.024	80.5
s_Faecalibaculum_rodentium	serum_Sphinganine	0.028 (0.007, 0.057)	<0.001	<0.001	0.022 (0.007, 0.048)	<0.001	<0.001	78.3
s_Faecalibaculum_rodentium	serum_Deoxycholic acid	0.028 (0.007, 0.057)	0.012	0.014	0.023 (0.005, 0.047)	0.008	0.034	82.5
s_Faecalibaculum_rodentium	serum_Docosapentaenoic acid (22n-3)	0.028 (0.008, 0.053)	<0.001	<0.001	0.023 (0.007, 0.049)	<0.001	<0.001	82.2
s_Faecalibaculum_rodentium	serum_Anandamide	0.028 (0.006, 0.055)	0.012	0.014	0.025 (0.009, 0.051)	<0.001	<0.001	90.3
s_Faecalibaculum_rodentium	serum_Alkergot	0.028 (0.009, 0.054)	0.008	0.013	0.021 (0.005, 0.043)	0.008	0.034	73.7
s_Faecalibaculum_rodentium	serum_1,6-Digalloyl-beta-D-glucopyranose	0.028 (0.008, 0.053)	0.016	0.017	0.021 (0.003, 0.043)	0.016	0.052	76.4

s_Faecalibaculum_rodentium	serum_1-Methylguanosine	0.028 (0.009, 0.056)	0.004	0.010	0.019 (0.003, 0.039)	0.028	0.070	68.2
s_Faecalibaculum_rodentium	serum_Retinal	0.028 (0.007, 0.053)	0.012	0.014	0.021 (0.005, 0.049)	0.012	0.043	74.4
s_Faecalibaculum_rodentium	serum_Phytosphingosine-1-P	0.028 (0.007, 0.055)	0.004	0.010	0.02 (0.004, 0.045)	0.008	0.034	73.4
s_Faecalibaculum_rodentium	serum_PS(18:1(11Z)/20:0)	0.028 (0.006, 0.055)	0.012	0.014	0.015 (0.001, 0.044)	0.036	0.079	54.8
s_Faecalibaculum_rodentium	serum_LysoPC(17:0/0:0)	0.028 (0.008, 0.053)	0.008	0.013	0.017 (0.005, 0.039)	<0.001	<0.001	62.6
s_Faecalibaculum_rodentium	serum_Glucosamine 6-phosphate	0.028 (0.006, 0.057)	0.008	0.013	0.013 (0.001, 0.035)	0.036	0.079	45.9
s_Faecalibaculum_rodentium	serum_Leukotriene C4	0.028 (0.005, 0.055)	0.020	0.020	0.018 (0.004, 0.045)	0.02	0.058	63.1
s_Faecalibaculum_rodentium	serum_gamma-Linolenic acid	0.028 (0.006, 0.052)	0.012	0.014	0.01 (0, 0.036)	0.028	0.070	36.9
g_Allobaculum	intestinal_Cortisone	0.014 (0.003, 0.028)	0.012	0.018	0.006 (0.003, 0.017)	0.004	0.029	44.1
g_Allobaculum	intestinal_Docosahexaenoic acid	0.014 (0.002, 0.031)	0.008	0.015	0.011 (0.001, 0.031)	0.012	0.045	79.9
g_Allobaculum	intestinal_Docosanol	0.014 (0.003, 0.028)	0.004	0.015	0.01 (0.001, 0.022)	0.02	0.058	71.2
g_Allobaculum	intestinal_Lauroyl diethanolamide	0.014 (0.003, 0.029)	0.016	0.019	0.005 (0, 0.012)	0.044	0.102	34.3
g_Allobaculum	intestinal_L-Kynurenine	0.014 (0.002, 0.03)	0.008	0.015	0.004 (0, 0.018)	0.032	0.079	27.2
g_Allobaculum	intestinal_Tryptophanamide	0.014 (0.002, 0.029)	0.016	0.019	0.007 (0.001, 0.019)	0.016	0.055	51.7
g_Allobaculum	intestinal_Ubiquinone-2	0.014 (0.002, 0.027)	0.028	0.028	0.013 (0.004, 0.031)	0.012	0.045	89.1
g_Allobaculum	serum_(±)-Tryptophan	0.014 (0.003, 0.029)	0.008	0.015	0.013 (0.003, 0.029)	0.02	0.058	92.7
g_Allobaculum	serum_Phenyllactic acid	0.014 (0.003, 0.029)	0.016	0.019	0.013 (0.003, 0.028)	0.012	0.045	92.1
g_Allobaculum	serum_Glycocholic acid	0.014 (0.003, 0.03)	0.004	0.015	0.014 (0.004, 0.026)	0.016	0.055	95.6
g_Allobaculum	serum_PC(16:0/20:4(5Z,8Z,11Z,14Z))	0.014 (0.004, 0.029)	0.004	0.015	0.012 (0.004, 0.025)	<0.001	<0.001	85.7
g_Allobaculum	serum_PC(16:0/18:1(9Z))	0.014 (0.003, 0.028)	0.016	0.019	0.009 (0.001, 0.026)	0.02	0.058	60.8

g_Allobaculum	serum_1-Palmitoylphosphatidylcholine	0.014 (0.003, 0.028)	0.020	0.021	0.013 (0.005, 0.025)	0.008	0.041	91.8
g_Allobaculum	serum_Sphinganine	0.014 (0.003, 0.03)	0.012	0.018	0.01 (0.004, 0.022)	<0.001	<0.001	73.2
g_Allobaculum	serum_Deoxycholic acid	0.014 (0.003, 0.028)	0.008	0.015	0.014 (0.005, 0.027)	<0.001	<0.001	96.7
g_Allobaculum	serum_Alkergot	0.014 (0.002, 0.028)	0.016	0.019	0.014 (0.005, 0.026)	0.008	0.041	99
g_Allobaculum	serum_1,6-Digalloyl-beta-D-glucopyranose	0.014 (0.002, 0.03)	0.024	0.025	0.01 (0.003, 0.021)	0.028	0.075	67.5
g_Allobaculum	serum_1-Methylguanosine	0.014 (0.003, 0.028)	0.004	0.015	0.013 (0.002, 0.026)	0.02	0.058	90.9
g_Allobaculum	serum_LysoPC(17:0/0:0)	0.014 (0.004, 0.029)	0.028	0.028	0.01 (0.004, 0.02)	0.008	0.041	72
g_Allobaculum	serum_Glucosamine 6-phosphate	0.014 (0.002, 0.03)	0.008	0.015	0.009 (0.001, 0.02)	0.032	0.079	61.8
s_Allobaculum_sp_539	intestinal_Cortisone	0.014 (0.004, 0.024)	0.004	0.006	0.005 (0.002, 0.013)	0.004	0.048	35
s_Allobaculum_sp_539	intestinal_Docosahexaenoic acid	0.014 (0.005, 0.026)	<0.001	<0.001	0.009 (0, 0.019)	0.036	0.104	63.3
s_Allobaculum_sp_539	intestinal_PC(16:0/18:1(9Z))	0.014 (0.005, 0.027)	0.004	0.006	0.006 (0, 0.014)	0.036	0.104	42.6
s_Allobaculum_sp_539	intestinal_Tryptophanamide	0.014 (0.004, 0.026)	0.004	0.006	0.006 (0.001, 0.014)	0.012	0.058	42.9
s_Allobaculum_sp_539	intestinal_Ubiquinone-2	0.014 (0.003, 0.026)	0.008	0.009	0.01 (0.003, 0.026)	0.016	0.068	74.6
s_Allobaculum_sp_539	serum_Arachidoyl Ethanolamide	0.014 (0.004, 0.025)	<0.001	<0.001	0.012 (0.004, 0.022)	0.008	0.058	89.2
s_Allobaculum_sp_539	serum_Stearoylethanolamide	0.014 (0.005, 0.026)	<0.001	<0.001	0.013 (0.005, 0.024)	0.012	0.058	97
s_Allobaculum_sp_539	serum_Heptadecanoic acid	0.014 (0.004, 0.025)	<0.001	<0.001	0.012 (0.003, 0.024)	0.032	0.100	88.8
s_Allobaculum_sp_539	serum_Palmitoylethanolamide	0.014 (0.005, 0.027)	0.004	0.006	0.011 (0.002, 0.022)	0.016	0.068	83
s_Allobaculum_sp_539	serum_Glycocholic acid	0.014 (0.005, 0.024)	0.004	0.006	0.011 (0.001, 0.021)	0.028	0.092	78.6
s_Allobaculum_sp_539	serum_PC(16:0/20:4(5Z,8Z,11Z,14Z))	0.014 (0.004, 0.026)	0.004	0.006	0.01 (0.004, 0.02)	<0.001	<0.001	75.3
s_Allobaculum_sp_539	serum_PC(16:0/18:1(9Z))	0.014 (0.005, 0.024)	0.004	0.006	0.007 (0.001, 0.021)	0.02	0.080	54.2

s_Allobaculum_sp_539	serum_1-Palmitoylphosphatidylcholine	0.014 (0.004, 0.025)	0.012	0.012	0.01 (0.004, 0.02)	0.004	0.048	76.9
s_Allobaculum_sp_539	serum_Sphinganine	0.014 (0.004, 0.027)	0.008	0.009	0.009 (0.003, 0.018)	0.008	0.058	65.2
s_Allobaculum_sp_539	serum_Deoxycholic acid	0.014 (0.004, 0.025)	0.008	0.009	0.011 (0.004, 0.021)	0.012	0.058	82.2
s_Allobaculum_sp_539	serum_Docosapentaenoic acid (22n-3)	0.014 (0.004, 0.024)	0.012	0.012	0.012 (0.003, 0.022)	0.012	0.058	88.9
s_Allobaculum_sp_539	serum_Alkergot	0.014 (0.005, 0.025)	0.004	0.006	0.011 (0.003, 0.019)	0.004	0.048	80.5
s_Allobaculum_sp_539	serum_1,6-Digalloyl-beta-D-glucopyranose	0.014 (0.004, 0.028)	0.004	0.006	0.008 (0.002, 0.017)	0.028	0.092	55.5
s_Allobaculum_sp_539	serum_Retinal	0.014 (0.004, 0.024)	0.004	0.006	0.013 (0.004, 0.023)	0.008	0.058	92.1
s_Allobaculum_sp_539	serum_Phytosphingosine-1-P	0.014 (0.004, 0.026)	0.004	0.006	0.012 (0.003, 0.025)	0.008	0.058	86.2
s_Allobaculum_sp_539	serum_LysoPC(17:0/0:0)	0.014 (0.004, 0.025)	0.008	0.009	0.009 (0.002, 0.016)	<0.001	<0.001	63.5
s_Bacteroidales_bacterium	intestinal_Cer(d18:1/16:0)	-0.133 (-0.276, -0.024)	0.016	0.022	-0.093 (-0.23, 0)	0.048	0.105	69.7
s_Bacteroidales_bacterium	intestinal_Cortisone	-0.133 (-0.268, -0.019)	0.016	0.022	-0.035 (-0.137, -0.009)	0.016	0.066	26.5
s_Bacteroidales_bacterium	intestinal_Docosahexaenoic acid	-0.133 (-0.269, -0.022)	0.020	0.022	-0.07 (-0.154, -0.015)	0.012	0.066	52.4
s_Bacteroidales_bacterium	intestinal_Docosanol	-0.133 (-0.264, -0.027)	0.020	0.022	-0.053 (-0.109, -0.009)	0.028	0.075	39.9
s_Bacteroidales_bacterium	intestinal_L-Kynurenine	-0.133 (-0.273, -0.025)	0.016	0.022	-0.022 (-0.156, -0.003)	0.036	0.084	16.8
s_Bacteroidales_bacterium	intestinal_PC(16:0/18:1(9Z))	-0.133 (-0.277, -0.014)	0.020	0.022	-0.089 (-0.262, -0.017)	0.008	0.064	66.8
s_Bacteroidales_bacterium	intestinal_Tryptophanamide	-0.133 (-0.275, -0.018)	0.020	0.022	-0.048 (-0.136, -0.006)	0.024	0.066	35.8
s_Bacteroidales_bacterium	intestinal_Ubiquinone-2	-0.133 (-0.26, -0.03)	0.004	0.022	-0.095 (-0.264, -0.038)	<0.001	<0.001	71.1
s_Bacteroidales_bacterium	serum_(±)-Tryptophan	-0.133 (-0.269, -0.025)	0.012	0.022	-0.107 (-0.241, -0.008)	0.036	0.084	80.3

s_Bacteroidales_bacterium	serum_Arachidoyl Ethanolamide	-0.133 (-0.267, -0.023)	0.016	0.022	-0.104 (-0.209, -0.027)	0.012	0.066	78.3
s_Bacteroidales_bacterium	serum_Phenyllactic acid	-0.133 (-0.268, -0.036)	0.008	0.022	-0.079 (-0.175, -0.012)	0.036	0.084	59.3
s_Bacteroidales_bacterium	serum_Stearoylethanolamide	-0.133 (-0.263, -0.023)	0.020	0.022	-0.114 (-0.229, -0.036)	0.008	0.064	85.9
s_Bacteroidales_bacterium	serum_Heptadecanoic acid	-0.133 (-0.287, -0.018)	0.012	0.022	-0.108 (-0.221, -0.016)	0.02	0.066	81
s_Bacteroidales_bacterium	serum_Esterase	-0.133 (-0.263, -0.024)	0.024	0.024	-0.08 (-0.176, -0.004)	0.044	0.099	60.4
s_Bacteroidales_bacterium	serum_Palmitoylethanolamide	-0.133 (-0.263, -0.024)	0.020	0.022	-0.101 (-0.217, -0.03)	0.004	0.048	76.1
s_Bacteroidales_bacterium	serum_Glycocholic acid	-0.133 (-0.258, -0.034)	0.008	0.022	-0.102 (-0.23, -0.026)	0.004	0.048	76.8
s_Bacteroidales_bacterium	serum_Sphingosine	-0.133 (-0.267, -0.032)	0.016	0.022	-0.098 (-0.176, -0.03)	0.008	0.064	73.9
s_Bacteroidales_bacterium	serum_PC(16:0/20:4(5Z,8Z,11Z,14Z))	-0.133 (-0.267, -0.022)	0.016	0.022	-0.107 (-0.223, -0.04)	<0.001	<0.001	80.3
s_Bacteroidales_bacterium	serum_PC(16:0/18:1(9Z))	-0.133 (-0.261, -0.022)	0.020	0.022	-0.089 (-0.188, -0.014)	0.02	0.066	66.8
s_Bacteroidales_bacterium	serum_1-Palmitoylphosphatidylcholine	-0.133 (-0.275, -0.027)	0.020	0.022	-0.094 (-0.215, -0.025)	0.016	0.066	71
s_Bacteroidales_bacterium	serum_Sphinganine	-0.133 (-0.276, -0.027)	0.024	0.024	-0.083 (-0.177, -0.016)	0.004	0.048	62.4
s_Bacteroidales_bacterium	serum_Deoxycholic acid	-0.133 (-0.266, -0.023)	0.012	0.022	-0.102 (-0.226, -0.021)	0.016	0.066	76.7
s_Bacteroidales_bacterium	serum_Docosapentaenoic acid (22n-3)	-0.133 (-0.274, -0.022)	0.024	0.024	-0.1 (-0.237, -0.016)	0.024	0.066	75.2
s_Bacteroidales_bacterium	serum_Anandamide	-0.133 (-0.281, -0.026)	0.004	0.022	-0.112 (-0.254, -0.036)	0.012	0.066	84

s_Bacteroidales_bacterium	serum_Alkergot	-0.133 (-0.272, -0.025)	0.016	0.022	-0.088 (-0.204, -0.006)	0.036	0.084	65.9
s_Bacteroidales_bacterium	serum_1,6-Digalloyl-beta-D-glucopyranose	-0.133 (-0.288, -0.024)	0.012	0.022	-0.074 (-0.191, -0.019)	0.02	0.066	55.5
s_Bacteroidales_bacterium	serum_1-Methylguanosine	-0.133 (-0.269, -0.027)	0.020	0.022	-0.083 (-0.177, -0.013)	0.024	0.066	62
s_Bacteroidales_bacterium	serum_Retinal	-0.133 (-0.279, -0.026)	0.012	0.022	-0.119 (-0.236, -0.032)	0.004	0.048	89.4
s_Bacteroidales_bacterium	serum_Phytosphingosine-1-P	-0.133 (-0.272, -0.024)	0.012	0.022	-0.066 (-0.137, -0.009)	0.016	0.066	49.9
s_Bacteroidales_bacterium	serum_PS(18:1(11Z)/20:0)	-0.133 (-0.274, -0.036)	0.004	0.022	-0.069 (-0.212, -0.007)	0.024	0.066	52.2
s_Bacteroidales_bacterium	serum_LysoPC(17:0/0:0)	-0.133 (-0.261, -0.032)	0.016	0.022	-0.086 (-0.203, -0.011)	0.024	0.066	64.9
s_Bacteroidales_bacterium	serum_Leukotriene C4	-0.133 (-0.275, -0.036)	0.004	0.022	-0.09 (-0.196, -0.016)	0.012	0.066	67.5
s_Bacteroidales_bacterium	serum_gamma-Linolenic acid	-0.133 (-0.272, -0.021)	0.016	0.022	-0.092 (-0.213, -0.007)	0.02	0.066	68.9
s_Muribaculaceae_bacterium_Isolate_080_Janvier	intestinal_Cortisone	0.063 (0.003, 0.11)	0.044	0.071	0.041 (0.016, 0.113)	0.012	0.045	65
s_Muribaculaceae_bacterium_Isolate_080_Janvier	intestinal_L-Kynurenine	0.063 (0.007, 0.108)	0.032	0.071	0.024 (0.002, 0.12)	0.04	0.103	39
s_Muribaculaceae_bacterium_Isolate_080_Janvier	intestinal_PC(16:0/18:1(9Z))	0.063 (0.005, 0.11)	0.032	0.071	0.039 (0.004, 0.118)	0.024	0.072	61.6
s_Muribaculaceae_bacterium_Isolate_080_Janvier	serum_PC(16:0/18:1(9Z))	0.063 (0.003, 0.106)	0.040	0.071	0.044 (0.005, 0.141)	0.012	0.045	70.9
s_Muribaculaceae_bacterium_Isolate_080_Janvier	serum_1,6-Digalloyl-beta-D-glucopyranose	0.063 (0.002, 0.104)	0.044	0.071	0.055 (0.016, 0.128)	0.004	0.036	88
s_Muribaculaceae_bacterium_Isolate_080_Janvier	serum_1-Methylguanosine	0.063 (0.002, 0.115)	0.044	0.071	0.059 (0.011, 0.127)	0.016	0.050	94.4
s_Muribaculaceae_bacterium_Isolate_080_Janvier	serum_LysoPC(17:0/0:0)	0.063 (0.003, 0.104)	0.040	0.071	0.053 (0.018, 0.119)	<0.001	<0.001	84.3

s_Muribaculaceae_bacterium_Isolate_080_Janvier	serum_Glucosamine 6-phosphate	0.063 (0.002, 0.11)	0.044	0.071	0.053 (0.001, 0.126)	0.048	0.119	84.4
s_Muribaculaceae_bacterium_Isolate_002_NCI	intestinal_Cortisone	-0.091 (-0.154, -0.015)	0.008	0.010	-0.037 (-0.132, -0.009)	0.012	0.041	40.4
s_Muribaculaceae_bacterium_Isolate_002_NCI	intestinal_Docosahexaenoic acid	-0.091 (-0.148, -0.023)	0.008	0.010	-0.042 (-0.143, -0.006)	0.02	0.053	46
s_Muribaculaceae_bacterium_Isolate_002_NCI	intestinal_Docosanol	-0.091 (-0.152, -0.025)	0.012	0.013	-0.043 (-0.119, -0.007)	0.016	0.046	47.1
s_Muribaculaceae_bacterium_Isolate_002_NCI	intestinal_PC(16:0/18:1(9Z))	-0.091 (-0.154, -0.023)	0.004	0.007	-0.039 (-0.094, -0.008)	0.016	0.046	42.2
s_Muribaculaceae_bacterium_Isolate_002_NCI	intestinal_Tryptophanamide	-0.091 (-0.15, -0.022)	<0.001	<0.001	-0.065 (-0.16, -0.011)	0.012	0.041	70.7
s_Muribaculaceae_bacterium_Isolate_002_NCI	intestinal_Ubiquinone-2	-0.091 (-0.153, -0.022)	0.008	0.010	-0.043 (-0.11, -0.016)	0.004	0.018	46.7
s_Muribaculaceae_bacterium_Isolate_002_NCI	serum_(±)-Tryptophan	-0.091 (-0.147, -0.027)	0.004	0.007	-0.055 (-0.159, -0.006)	0.024	0.060	60
s_Muribaculaceae_bacterium_Isolate_002_NCI	serum_Arachidoyl Ethanolamide	-0.091 (-0.148, -0.023)	0.004	0.007	-0.061 (-0.141, -0.019)	0.004	0.018	66.6
s_Muribaculaceae_bacterium_Isolate_002_NCI	serum_Phenyllactic acid	-0.091 (-0.153, -0.02)	0.012	0.013	-0.045 (-0.122, -0.006)	0.028	0.067	48.9
s_Muribaculaceae_bacterium_Isolate_002_NCI	serum_Stearoylethanolamide	-0.091 (-0.151, -0.025)	0.004	0.007	-0.061 (-0.135, -0.018)	0.004	0.018	67.2
s_Muribaculaceae_bacterium_Isolate_002_NCI	serum_Heptadecanoic acid	-0.091 (-0.148, -0.026)	<0.001	<0.001	-0.052 (-0.125, -0.01)	0.004	0.018	57
s_Muribaculaceae_bacterium_Isolate_002_NCI	serum_Esterase	-0.091 (-0.149, -0.021)	0.004	0.007	-0.034 (-0.102, -0.004)	0.02	0.053	36.9
s_Muribaculaceae_bacterium_Isolate_002_NCI	serum_Palmitoylethanolamide	-0.091 (-0.144, -0.023)	0.008	0.010	-0.053 (-0.129, -0.016)	<0.001	<0.001	58.2
s_Muribaculaceae_bacterium_Isolate_002_NCI	serum_Glycocholic acid	-0.091 (-0.151, -0.018)	0.008	0.010	-0.051 (-0.13, -0.011)	<0.001	<0.001	55.4
s_Muribaculaceae_bacterium_Isolate_002_NCI	serum_Sphingosine	-0.091 (-0.151, -0.02)	0.012	0.013	-0.067 (-0.192, -0.019)	0.004	0.018	72.8

s_Muribaculaceae_bacterium_Isolate_002_NCI	serum_PC(16:0/20:4(5Z,8Z,11Z,14Z))	-0.091 (-0.15, -0.026)	0.008	0.010	-0.063 (-0.134, -0.024)	0.004	0.018	68.5
s_Muribaculaceae_bacterium_Isolate_002_NCI	serum_PC(16:0/18:1(9Z))	-0.091 (-0.148, -0.021)	0.012	0.013	-0.043 (-0.098, -0.006)	0.016	0.046	46.8
s_Muribaculaceae_bacterium_Isolate_002_NCI	serum_1-Palmitoylphosphatidylcholine	-0.091 (-0.146, -0.018)	<0.001	<0.001	-0.05 (-0.108, -0.014)	0.004	0.018	54.5
s_Muribaculaceae_bacterium_Isolate_002_NCI	serum_Sphinganine	-0.091 (-0.151, -0.03)	0.004	0.007	-0.045 (-0.096, -0.01)	0.008	0.032	48.8
s_Muribaculaceae_bacterium_Isolate_002_NCI	serum_Deoxycholic acid	-0.091 (-0.155, -0.027)	0.008	0.010	-0.061 (-0.145, -0.016)	<0.001	<0.001	66.5
s_Muribaculaceae_bacterium_Isolate_002_NCI	serum_Docosapentaenoic acid (22n-3)	-0.091 (-0.149, -0.025)	0.008	0.010	-0.052 (-0.124, -0.009)	<0.001	<0.001	56.4
s_Muribaculaceae_bacterium_Isolate_002_NCI	serum_Anandamide	-0.091 (-0.149, -0.024)	0.016	0.016	-0.057 (-0.122, -0.019)	<0.001	<0.001	62.6
s_Muribaculaceae_bacterium_Isolate_002_NCI	serum_Alkergot	-0.091 (-0.146, -0.03)	0.004	0.007	-0.06 (-0.144, -0.02)	<0.001	<0.001	65.8
s_Muribaculaceae_bacterium_Isolate_002_NCI	serum_1,6-Digalloyl-beta-D-glucopyranose	-0.091 (-0.149, -0.028)	0.004	0.007	-0.048 (-0.131, -0.012)	<0.001	<0.001	52.7
s_Muribaculaceae_bacterium_Isolate_002_NCI	serum_1-Methylguanosine	-0.091 (-0.149, -0.029)	0.008	0.010	-0.045 (-0.11, -0.003)	0.036	0.081	48.8
s_Muribaculaceae_bacterium_Isolate_002_NCI	serum_Retinal	-0.091 (-0.143, -0.023)	0.008	0.010	-0.048 (-0.102, -0.006)	0.016	0.046	52.3
s_Muribaculaceae_bacterium_Isolate_002_NCI	serum_Phytosphingosine-1-P	-0.091 (-0.152, -0.028)	0.008	0.010	-0.047 (-0.11, -0.007)	0.024	0.060	51.6
s_Muribaculaceae_bacterium_Isolate_002_NCI	serum_PS(18:1(11Z)/20:0)	-0.091 (-0.153, -0.026)	0.008	0.010	-0.027 (-0.074, -0.001)	0.036	0.081	29.4
s_Muribaculaceae_bacterium_Isolate_002_NCI	serum_LysoPC(17:0/0:0)	-0.091 (-0.151, -0.025)	0.004	0.007	-0.043 (-0.091, -0.012)	0.008	0.032	47.2
s_Muribaculaceae_bacterium_Isolate_002_NCI	serum_Glucosamine 6-phosphate	-0.091 (-0.154, -0.028)	0.004	0.007	-0.032 (-0.078, 0)	0.044	0.093	34.8

s_Muribaculaceae_bacterium_Isolate_002_NCI	serum_Leukotriene C4	-0.091 (-0.155, -0.029)	<0.001	<0.001	-0.037 (-0.082, -0.002)	0.044	0.093	40.9
o_Coriobacteriales	intestinal_Cortisone	0.043 (0.009, 0.076)	0.012	0.015	0.014 (0.006, 0.042)	<0.001	<0.001	31.8
o_Coriobacteriales	intestinal_Docosahexaenoic acid	0.043 (0.011, 0.082)	0.012	0.015	0.025 (0.005, 0.053)	0.02	0.144	58.5
o_Coriobacteriales	intestinal_L-Kynurenine	0.043 (0.009, 0.082)	0.008	0.013	0.007 (0.001, 0.032)	0.036	0.173	16.4
o_Coriobacteriales	intestinal_Tryptophanamide	0.043 (0.009, 0.078)	0.016	0.018	0.019 (0.006, 0.052)	0.008	0.115	44.4
o_Coriobacteriales	intestinal_Ubiquinone-2	0.043 (0.009, 0.08)	0.016	0.018	0.031 (0.009, 0.062)	<0.001	<0.001	71.8
o_Coriobacteriales	serum_Stearoylethanolamide	0.043 (0.008, 0.081)	0.012	0.015	0.036 (0.003, 0.071)	0.04	0.173	82.3
o_Coriobacteriales	serum_Sphingosine	0.043 (0.011, 0.078)	0.012	0.015	0.023 (0.004, 0.043)	0.028	0.173	53.1
o_Coriobacteriales	serum_Sphinganine	0.043 (0.009, 0.074)	0.008	0.013	0.032 (0.007, 0.064)	0.016	0.144	74
o_Coriobacteriales	serum_Deoxycholic acid	0.043 (0.013, 0.08)	0.004	0.009	0.032 (0.007, 0.06)	0.02	0.144	73.3
o_Coriobacteriales	serum_Docosapentaenoic acid (22n-3)	0.043 (0.007, 0.08)	0.016	0.018	0.029 (0, 0.066)	0.048	0.173	67
o_Coriobacteriales	serum_Anandamide	0.043 (0.012, 0.082)	0.012	0.015	0.032 (0.002, 0.074)	0.044	0.173	73.7
o_Coriobacteriales	serum_Alkergot	0.043 (0.013, 0.08)	0.016	0.018	0.024 (0.001, 0.051)	0.044	0.173	56.1
o_Coriobacteriales	serum_1,6-Digalloyl-beta-D-glucopyranose	0.043 (0.012, 0.077)	0.004	0.009	0.028 (0.001, 0.057)	0.04	0.173	64.8
o_Coriobacteriales	serum_Retinal	0.043 (0.012, 0.084)	0.004	0.009	0.031 (0.005, 0.069)	0.02	0.144	71.2
o_Coriobacteriales	serum_Phytosphingosine-1-P	0.043 (0.013, 0.078)	0.004	0.009	0.022 (0.004, 0.048)	0.012	0.144	51.5
o_Coriobacteriales	serum_PS(18:1(11Z)/20:0)	0.043 (0.012, 0.077)	0.012	0.015	0.022 (0.003, 0.062)	0.008	0.115	50.6
o_Coriobacteriales	serum_LysoPC(17:0/0:0)	0.043 (0.011, 0.076)	0.008	0.013	0.029 (0.002, 0.075)	0.048	0.173	67.7
f_Odoribacteraceae	intestinal_Cortisone	-0.07 (-0.123, -0.015)	0.028	0.028	-0.025 (-0.082, -0.009)	<0.001	<0.001	36.1
f_Odoribacteraceae	intestinal_Docosahexaenoic acid	-0.07 (-0.121, -0.019)	<0.001	<0.001	-0.042 (-0.103, -0.003)	0.044	0.122	59.7
f_Odoribacteraceae	intestinal_Docosanol	-0.07 (-0.128, -0.017)	0.016	0.019	-0.032 (-0.112, -0.003)	0.028	0.092	45.4
f_Odoribacteraceae	intestinal_PC(16:0/18:1(9Z))	-0.07 (-0.123, -0.022)	0.012	0.017	-0.039 (-0.092, -0.012)	0.012	0.062	54.9

f_Odoribacteraceae	intestinal_Tryptophanamide	-0.07 (-0.119, -0.02)	0.008	0.017	-0.035 (-0.08, -0.009)	0.024	0.086	49.1
f_Odoribacteraceae	intestinal_Ubiquinone-2	-0.07 (-0.125, -0.02)	0.004	0.015	-0.048 (-0.099, -0.014)	0.016	0.077	67.5
f_Odoribacteraceae	serum_(±)-Tryptophan	-0.07 (-0.12, -0.017)	0.004	0.015	-0.04 (-0.087, -0.001)	0.044	0.122	56.6
f_Odoribacteraceae	serum_Arachidoyl Ethanolamide	-0.07 (-0.12, -0.017)	0.016	0.019	-0.046 (-0.096, -0.008)	0.012	0.062	65.2
f_Odoribacteraceae	serum_Stearoylethanolamide	-0.07 (-0.119, -0.018)	<0.001	<0.001	-0.051 (-0.092, -0.014)	0.008	0.058	72.3
f_Odoribacteraceae	serum_Heptadecanoic acid	-0.07 (-0.121, -0.014)	0.012	0.017	-0.045 (-0.096, -0.008)	0.012	0.062	63.6
f_Odoribacteraceae	serum_Palmitoylethanolamide	-0.07 (-0.122, -0.014)	0.008	0.017	-0.046 (-0.093, -0.008)	0.012	0.062	64.8
f_Odoribacteraceae	serum_Glycocholic acid	-0.07 (-0.125, -0.021)	0.012	0.017	-0.042 (-0.086, -0.008)	0.028	0.092	59.6
f_Odoribacteraceae	serum_Sphingosine	-0.07 (-0.123, -0.024)	0.012	0.017	-0.056 (-0.113, -0.008)	0.036	0.108	79.9
f_Odoribacteraceae	serum_PC(16:0/20:4(5Z,8Z,11Z,14Z))	-0.07 (-0.121, -0.016)	<0.001	<0.001	-0.044 (-0.092, -0.016)	<0.001	<0.001	62.6
f_Odoribacteraceae	serum_PC(16:0/18:1(9Z))	-0.07 (-0.122, -0.02)	0.024	0.025	-0.033 (-0.076, -0.003)	0.032	0.100	46.8
f_Odoribacteraceae	serum_1-Palmitoylphosphatidylcholine	-0.07 (-0.121, -0.018)	0.012	0.017	-0.04 (-0.09, -0.007)	0.024	0.086	57.4
f_Odoribacteraceae	serum_Sphinganine	-0.07 (-0.122, -0.019)	0.004	0.015	-0.038 (-0.074, -0.009)	0.008	0.058	54.3
f_Odoribacteraceae	serum_Deoxycholic acid	-0.07 (-0.124, -0.019)	0.004	0.015	-0.043 (-0.088, -0.007)	0.024	0.086	61.2
f_Odoribacteraceae	serum_Docosapentaenoic acid (22n-3)	-0.07 (-0.123, -0.012)	0.028	0.028	-0.045 (-0.095, -0.006)	0.02	0.085	64
f_Odoribacteraceae	serum_Anandamide	-0.07 (-0.118, -0.019)	0.012	0.017	-0.05 (-0.115, -0.017)	<0.001	<0.001	71.6
f_Odoribacteraceae	serum_Alkergot	-0.07 (-0.119, -0.018)	0.008	0.017	-0.037 (-0.074, -0.006)	0.004	0.041	52.6
f_Odoribacteraceae	serum_1,6-Digalloyl-beta-D-glucopyranose	-0.07 (-0.125, -0.017)	0.016	0.019	-0.034 (-0.074, -0.012)	<0.001	<0.001	47.9
f_Odoribacteraceae	serum_Retinal	-0.07 (-0.115, -0.015)	0.024	0.025	-0.045 (-0.091, -0.012)	0.004	0.041	63.3
f_Odoribacteraceae	serum_Phytosphingosine-1-P	-0.07 (-0.118, -0.018)	0.012	0.017	-0.047 (-0.098, -0.01)	0.008	0.058	66.8
f_Odoribacteraceae	serum_LysoPC(17:0/0:0)	-0.07 (-0.121, -0.021)	0.008	0.017	-0.036 (-0.078, -0.005)	0.02	0.085	51.6

f_Odoribacteraceae	serum_Glucosamine 6-phosphate	-0.07 (-0.119, -0.019)	0.004	0.015	-0.024 (-0.062, -0.001)	0.048	0.128	34.3
f_Odoribacteraceae	serum_Leukotriene C4	-0.07 (-0.125, -0.023)	0.004	0.015	-0.038 (-0.092, -0.007)	0.004	0.041	53.9
f_Coriobacteriaceae	intestinal_Cortisone	0.044 (0.008, 0.081)	0.016	0.020	0.014 (0.006, 0.045)	0.004	0.072	32.3
f_Coriobacteriaceae	intestinal_Docosahexaenoic acid	0.044 (0.009, 0.083)	0.016	0.020	0.026 (0.005, 0.056)	0.012	0.144	59.3
f_Coriobacteriaceae	intestinal_Estrogen	0.044 (0.008, 0.08)	0.020	0.022	0.012 (0.001, 0.032)	0.044	0.144	26.4
f_Coriobacteriaceae	intestinal_Tryptophanamide	0.044 (0.009, 0.077)	0.004	0.020	0.02 (0.005, 0.048)	<0.001	<0.001	45.3
f_Coriobacteriaceae	intestinal_Ubiquinone-2	0.044 (0.007, 0.08)	0.020	0.022	0.032 (0.006, 0.065)	0.032	0.144	73.4
f_Coriobacteriaceae	serum_Arachidoyl Ethanolamide	0.044 (0.011, 0.081)	0.020	0.022	0.036 (0.007, 0.086)	0.024	0.144	81.4
f_Coriobacteriaceae	serum_Stearoylethanolamide	0.044 (0.007, 0.078)	0.016	0.020	0.037 (0.009, 0.083)	0.016	0.144	83.4
f_Coriobacteriaceae	serum_Glycocholic acid	0.044 (0.011, 0.082)	0.012	0.020	0.033 (0.002, 0.069)	0.04	0.144	74.1
f_Coriobacteriaceae	serum_Sphingosine	0.044 (0.007, 0.08)	0.020	0.022	0.024 (0.002, 0.049)	0.028	0.144	53.6
f_Coriobacteriaceae	serum_PC(16:0/18:1(9Z))	0.044 (0.007, 0.076)	0.016	0.020	0.035 (0, 0.086)	0.04	0.144	79.7
f_Coriobacteriaceae	serum_Sphinganine	0.044 (0.015, 0.077)	0.004	0.020	0.033 (0.004, 0.076)	0.036	0.144	75.1
f_Coriobacteriaceae	serum_Deoxycholic acid	0.044 (0.008, 0.081)	0.012	0.020	0.033 (0.005, 0.067)	0.036	0.144	74.4
f_Coriobacteriaceae	serum_Docosapentaenoic acid (22n-3)	0.044 (0.008, 0.082)	0.012	0.020	0.03 (0.002, 0.072)	0.036	0.144	68.1
f_Coriobacteriaceae	serum_Anandamide	0.044 (0.011, 0.079)	0.016	0.020	0.033 (0.007, 0.074)	0.02	0.144	74.7
f_Coriobacteriaceae	serum_Alkergot	0.044 (0.008, 0.082)	0.008	0.020	0.025 (0.002, 0.053)	0.04	0.144	57
f_Coriobacteriaceae	serum_1,6-Digalloyl-beta-D-glucopyranose	0.044 (0.01, 0.085)	0.012	0.020	0.029 (0.001, 0.058)	0.04	0.144	66.6
f_Coriobacteriaceae	serum_1-Methylguanosine	0.044 (0.01, 0.084)	0.020	0.022	0.024 (0, 0.058)	0.048	0.144	53.9
f_Coriobacteriaceae	serum_Retinal	0.044 (0.008, 0.082)	0.008	0.020	0.032 (0.007, 0.061)	0.004	0.072	71.8
f_Coriobacteriaceae	serum_Phytosphingosine-1-P	0.044 (0.011, 0.081)	0.020	0.022	0.022 (0.003, 0.045)	0.016	0.144	51.1
f_Coriobacteriaceae	serum_PS(18:1(11Z)/20:0)	0.044 (0.005, 0.082)	0.028	0.029	0.023 (0.001, 0.063)	0.02	0.144	51.9
f_Coriobacteriaceae	serum_LysoPC(17:0/0:0)	0.044 (0.012, 0.08)	0.004	0.020	0.03 (0.001, 0.076)	0.048	0.144	68.8

f_Coriobacteriaceae	serum_gamma-Linolenic acid	0.044 (0.006, 0.085)	0.024	0.025	0.034 (0.004, 0.079)	0.02	0.144	77.5
s_Bacteroidales_bacterium_55_9	intestinal_Cortisone	-0.039 (-0.06, -0.016)	<0.001	<0.001	-0.008 (-0.025, -0.003)	0.004	0.288	20.4
s_Bacteroidales_bacterium_55_9	intestinal_Tryptophanamide	-0.039 (-0.063, -0.019)	<0.001	<0.001	-0.01 (-0.024, -0.001)	0.032	0.561	25.6
s_Bacteroidales_bacterium_55_9	serum_PC(16:0/20:4(5Z,8Z,11Z,14Z))	-0.039 (-0.062, -0.016)	<0.001	<0.001	-0.019 (-0.043, -0.001)	0.028	0.561	49.4
s_Bacteroidales_bacterium_55_9	serum_LysoPC(17:0/0:0)	-0.039 (-0.063, -0.014)	<0.001	<0.001	-0.018 (-0.031, -0.002)	0.032	0.561	47
s_Coriobacteriaceae_bacterium	intestinal_Cortisone	0.023 (0.009, 0.037)	<0.001	<0.001	0.006 (0.002, 0.019)	0.004	0.288	24.9
s_Coriobacteriaceae_bacterium	intestinal_Tryptophanamide	0.023 (0.008, 0.037)	<0.001	<0.001	0.007 (0.001, 0.019)	0.032	0.600	33.1
s_Alistipes_senegalensis	intestinal_Cortisone	-0.071 (-0.114, -0.034)	<0.001	<0.001	-0.016 (-0.051, -0.006)	0.004	0.144	22.2
s_Alistipes_senegalensis	intestinal_Tryptophanamide	-0.071 (-0.107, -0.036)	<0.001	<0.001	-0.02 (-0.051, -0.004)	0.004	0.144	28
s_Alistipes_senegalensis	serum_PC(16:0/20:4(5Z,8Z,11Z,14Z))	-0.071 (-0.113, -0.031)	<0.001	<0.001	-0.039 (-0.085, -0.001)	0.044	0.288	54.4
s_Alistipes_senegalensis	serum_Sphinganine	-0.071 (-0.107, -0.034)	<0.001	<0.001	-0.034 (-0.066, -0.002)	0.04	0.288	47.8
s_Alistipes_senegalensis	serum_Anandamide	-0.071 (-0.11, -0.031)	0.004	0.004	-0.047 (-0.086, -0.012)	0.012	0.288	65.7
s_Alistipes_senegalensis	serum_Retinal	-0.071 (-0.111, -0.035)	0.004	0.004	-0.045 (-0.089, -0.005)	0.032	0.288	62.9
s_Alistipes_senegalensis	serum_LysoPC(17:0/0:0)	-0.071 (-0.109, -0.033)	<0.001	<0.001	-0.034 (-0.066, -0.006)	0.024	0.288	47.3
s_Alistipes_senegalensis	serum_Leukotriene C4	-0.071 (-0.111, -0.038)	<0.001	<0.001	-0.028 (-0.075, -0.004)	0.016	0.288	39.9
s_Alistipes_senegalensis	serum_gamma-Linolenic acid	-0.071 (-0.108, -0.035)	<0.001	<0.001	-0.024 (-0.068, -0.002)	0.028	0.288	33.7
s_Alistipes_finegoldii	intestinal_Cortisone	-0.064 (-0.101, -0.025)	<0.001	<0.001	-0.014 (-0.05, -0.006)	0.008	0.247	22.5
s_Alistipes_finegoldii	intestinal_L-Kynurenine	-0.064 (-0.1, -0.031)	<0.001	<0.001	-0.007 (-0.042, 0)	0.048	0.267	11.2
s_Alistipes_finegoldii	intestinal_Tryptophanamide	-0.064 (-0.098, -0.03)	0.004	0.004	-0.018 (-0.045, -0.002)	0.036	0.267	28.2
s_Alistipes_finegoldii	serum_PC(16:0/20:4(5Z,8Z,11Z,14Z))	-0.064 (-0.097, -0.031)	<0.001	<0.001	-0.035 (-0.084, -0.004)	0.02	0.247	55.1

s_Alistipes_finegoldii	serum_Anandamide	-0.064 (-0.099, -0.027)	<0.001	<0.001	-0.043 (-0.081, -0.007)	0.024	0.247	66.9
s_Alistipes_finegoldii	serum_Retinal	-0.064 (-0.104, -0.031)	<0.001	<0.001	-0.041 (-0.095, -0.006)	0.016	0.247	64.2
s_Alistipes_finegoldii	serum_LysoPC(17:0/0:0)	-0.064 (-0.095, -0.03)	<0.001	<0.001	-0.031 (-0.06, -0.008)	0.02	0.247	47.9
s_Alistipes_finegoldii	serum_Leukotriene C4	-0.064 (-0.099, -0.033)	<0.001	<0.001	-0.026 (-0.059, -0.005)	0.024	0.247	40.5
s_Alistipes_finegoldii	serum_gamma-Linolenic acid	-0.064 (-0.101, -0.034)	<0.001	<0.001	-0.022 (-0.063, -0.003)	0.02	0.247	33.8
g_Odoribacter	intestinal_Cortisone	-0.072 (-0.12, -0.021)	0.016	0.017	-0.025 (-0.088, -0.01)	0.004	0.058	34.9
g_Odoribacter	intestinal_Docosahexaenoic acid	-0.072 (-0.117, -0.024)	0.008	0.013	-0.042 (-0.091, -0.003)	0.036	0.100	58.2
g_Odoribacter	intestinal_PC(16:0/18:1(9Z))	-0.072 (-0.124, -0.024)	<0.001	<0.001	-0.038 (-0.076, -0.007)	0.024	0.091	52.6
g_Odoribacter	intestinal_Tryptophanamide	-0.072 (-0.116, -0.016)	0.020	0.020	-0.033 (-0.08, -0.008)	0.012	0.072	46.6
g_Odoribacter	intestinal_Ubiquinone-2	-0.072 (-0.123, -0.02)	0.016	0.017	-0.047 (-0.097, -0.012)	0.016	0.077	65.9
g_Odoribacter	serum_(±)-Tryptophan	-0.072 (-0.117, -0.015)	0.016	0.017	-0.039 (-0.086, -0.003)	0.032	0.096	53.8
g_Odoribacter	serum_Arachidoyl Ethanolamide	-0.072 (-0.121, -0.019)	0.016	0.017	-0.046 (-0.092, -0.011)	0.016	0.077	63.5
g_Odoribacter	serum_Stearoylethanolamide	-0.072 (-0.124, -0.023)	0.016	0.017	-0.05 (-0.108, -0.013)	0.016	0.077	70.2
g_Odoribacter	serum_Heptadecanoic acid	-0.072 (-0.12, -0.021)	0.004	0.013	-0.044 (-0.088, -0.005)	0.024	0.091	61.8
g_Odoribacter	serum_Palmitoylethanolamide	-0.072 (-0.125, -0.022)	0.004	0.013	-0.045 (-0.098, -0.007)	0.036	0.100	62.9
g_Odoribacter	serum_Glycocholic acid	-0.072 (-0.126, -0.017)	0.012	0.015	-0.041 (-0.086, -0.007)	0.028	0.096	57.7
g_Odoribacter	serum_Sphingosine	-0.072 (-0.12, -0.02)	0.012	0.015	-0.057 (-0.117, -0.005)	0.032	0.096	79.7
g_Odoribacter	serum_PC(16:0/20:4(5Z,8Z,11Z,14Z))	-0.072 (-0.128, -0.02)	0.004	0.013	-0.043 (-0.09, -0.016)	<0.001	<0.001	60.3
g_Odoribacter	serum_PC(16:0/18:1(9Z))	-0.072 (-0.125, -0.022)	0.008	0.013	-0.033 (-0.083, -0.006)	0.024	0.091	45.2
g_Odoribacter	serum_1-Palmitoylphosphatidylcholine	-0.072 (-0.12, -0.017)	0.008	0.013	-0.04 (-0.088, -0.012)	0.008	0.064	55.8
g_Odoribacter	serum_Sphinganine	-0.072 (-0.122, -0.02)	0.008	0.013	-0.038 (-0.077, -0.011)	<0.001	<0.001	53.2
g_Odoribacter	serum_Deoxycholic acid	-0.072 (-0.124, -0.015)	0.016	0.017	-0.043 (-0.092, -0.009)	0.008	0.064	59.7

g_Odoribacter	serum_Docosapentaenoic acid (22n-3)	-0.072 (-0.125, -0.022)	0.008	0.013	-0.045 (-0.091, -0.008)	0.008	0.064	62.4
g_Odoribacter	serum_Anandamide	-0.072 (-0.119, -0.019)	0.008	0.013	-0.05 (-0.1, -0.013)	0.008	0.064	70
g_Odoribacter	serum_Alkergot	-0.072 (-0.122, -0.02)	<0.001	<0.001	-0.037 (-0.086, -0.007)	0.012	0.072	51.7
g_Odoribacter	serum_1,6-Digalloyl-beta-D-glucopyranose	-0.072 (-0.128, -0.017)	0.016	0.017	-0.033 (-0.068, -0.006)	0.032	0.096	46.5
g_Odoribacter	serum_1-Methylguanosine	-0.072 (-0.124, -0.024)	0.004	0.013	-0.037 (-0.085, -0.003)	0.032	0.096	52
g_Odoribacter	serum_Retinal	-0.072 (-0.127, -0.02)	0.012	0.015	-0.045 (-0.088, -0.011)	0.004	0.058	62.6
g_Odoribacter	serum_Phytosphingosine-1-P	-0.072 (-0.12, -0.02)	<0.001	<0.001	-0.048 (-0.092, -0.009)	<0.001	<0.001	67
g_Odoribacter	serum_LysoPC(17:0/0:0)	-0.072 (-0.12, -0.016)	0.008	0.013	-0.036 (-0.076, -0.007)	0.012	0.072	50
g_Odoribacter	serum_Glucosamine 6-phosphate	-0.072 (-0.125, -0.016)	0.012	0.015	-0.024 (-0.058, -0.001)	0.04	0.107	33.4
g_Odoribacter	serum_Leukotriene C4	-0.072 (-0.12, -0.02)	0.008	0.013	-0.037 (-0.085, -0.007)	0.024	0.091	51.2
g_Odoribacter	serum_gamma-Linolenic acid	-0.072 (-0.124, -0.019)	0.012	0.015	-0.025 (-0.068, 0)	0.048	0.123	34.8
s_Alistipes_sp_58_9_plus	intestinal_Cortisone	-0.038 (-0.057, -0.019)	<0.001	<0.001	-0.008 (-0.025, -0.003)	0.008	0.544	20.2
s_Alistipes_sp_58_9_plus	intestinal_Tryptophanamide	-0.038 (-0.058, -0.017)	<0.001	<0.001	-0.01 (-0.021, -0.001)	0.032	0.544	25.4
s_Alistipes_sp_58_9_plus	serum_Retinal	-0.038 (-0.059, -0.014)	<0.001	<0.001	-0.023 (-0.047, 0)	0.048	0.544	62.6
s_Alistipes_sp_58_9_plus	serum_LysoPC(17:0/0:0)	-0.038 (-0.058, -0.015)	<0.001	<0.001	-0.017 (-0.033, -0.001)	0.044	0.544	45.9
s_Alistipes_sp_58_9_plus	serum_Leukotriene C4	-0.038 (-0.057, -0.014)	<0.001	<0.001	-0.014 (-0.03, -0.002)	0.032	0.544	38.4
s_Alistipes_sp_	intestinal_Cortisone	-0.045 (-0.074, -0.015)	<0.001	<0.001	-0.011 (-0.038, -0.004)	0.004	0.288	24.5
s_Alistipes_sp_	intestinal_Tryptophanamide	-0.045 (-0.073, -0.019)	<0.001	<0.001	-0.014 (-0.035, -0.001)	0.04	0.401	32
s_Alistipes_sp_	serum_PC(16:0/20:4(5Z,8Z,11Z,14Z))	-0.045 (-0.074, -0.016)	0.008	0.008	-0.031 (-0.064, -0.003)	0.02	0.401	70.1
s_Alistipes_sp_	serum_Retinal	-0.045 (-0.073, -0.016)	<0.001	<0.001	-0.036 (-0.065, -0.004)	0.04	0.401	79.6
s_Alistipes_sp_	serum_LysoPC(17:0/0:0)	-0.045 (-0.071, -0.017)	<0.001	<0.001	-0.026 (-0.044, -0.005)	0.024	0.401	57
s_Alistipes_sp_	serum_Leukotriene C4	-0.045 (-0.073, -0.017)	<0.001	<0.001	-0.021 (-0.053, -0.003)	0.012	0.401	47.6
s_Bacteroides_caecimuris	intestinal_Cortisone	-0.067 (-0.13, -0.013)	0.004	0.011	-0.024 (-0.076, -0.01)	0.004	0.036	35.5

s_Bacteroides_caecimuris	intestinal_Docosahexaenoic acid	-0.067 (-0.138, -0.016)	0.008	0.013	-0.047 (-0.093, -0.009)	0.02	0.069	69.6
s_Bacteroides_caecimuris	intestinal_Docosanol	-0.067 (-0.139, -0.014)	0.008	0.013	-0.033 (-0.078, -0.004)	0.02	0.069	48.8
s_Bacteroides_caecimuris	intestinal_PC(16:0/18:1(9Z))	-0.067 (-0.132, -0.015)	0.012	0.014	-0.048 (-0.11, -0.015)	<0.001	<0.001	70.6
s_Bacteroides_caecimuris	intestinal_Tolmetin	-0.067 (-0.126, -0.013)	0.004	0.011	-0.022 (-0.056, -0.001)	0.036	0.104	31.9
s_Bacteroides_caecimuris	intestinal_Tryptophanamide	-0.067 (-0.136, -0.015)	0.004	0.011	-0.032 (-0.076, -0.009)	0.016	0.069	47.8
s_Bacteroides_caecimuris	intestinal_Ubiquinone-2	-0.067 (-0.135, -0.019)	0.004	0.011	-0.054 (-0.175, -0.03)	<0.001	<0.001	80.2
s_Bacteroides_caecimuris	serum_Arachidoyl Ethanolamide	-0.067 (-0.125, -0.012)	0.004	0.011	-0.063 (-0.12, -0.018)	0.016	0.069	93.9
s_Bacteroides_caecimuris	serum_Stearoylethanolamide	-0.067 (-0.14, -0.012)	0.012	0.014	-0.067 (-0.126, -0.015)	0.02	0.069	99.5
s_Bacteroides_caecimuris	serum_Heptadecanoic acid	-0.067 (-0.14, -0.017)	0.004	0.011	-0.063 (-0.139, -0.006)	0.028	0.088	93.7
s_Bacteroides_caecimuris	serum_Palmitoylethanolamide	-0.067 (-0.133, -0.015)	0.004	0.011	-0.063 (-0.115, -0.017)	0.028	0.088	93
s_Bacteroides_caecimuris	serum_Glycocholic acid	-0.067 (-0.133, -0.018)	0.008	0.013	-0.059 (-0.107, -0.013)	0.02	0.069	87.7
s_Bacteroides_caecimuris	serum_PC(16:0/20:4(5Z,8Z,11Z,14Z))	-0.067 (-0.134, -0.017)	<0.001	<0.001	-0.057 (-0.109, -0.024)	0.004	0.036	84.7
s_Bacteroides_caecimuris	serum_PC(16:0/18:1(9Z))	-0.067 (-0.137, -0.014)	0.008	0.013	-0.045 (-0.083, -0.001)	0.04	0.107	66.9
s_Bacteroides_caecimuris	serum_1-Palmitoylphosphatidylcholine	-0.067 (-0.135, -0.013)	0.016	0.017	-0.053 (-0.117, -0.011)	0.016	0.069	79.1
s_Bacteroides_caecimuris	serum_Sphinganine	-0.067 (-0.137, -0.021)	<0.001	<0.001	-0.046 (-0.09, -0.016)	0.004	0.036	67.9
s_Bacteroides_caecimuris	serum_Deoxycholic acid	-0.067 (-0.135, -0.01)	0.016	0.017	-0.056 (-0.104, -0.018)	0.008	0.052	83.7
s_Bacteroides_caecimuris	serum_Docosapentaenoic acid (22n-3)	-0.067 (-0.136, -0.015)	0.004	0.011	-0.058 (-0.118, -0.013)	0.008	0.052	85.8
s_Bacteroides_caecimuris	serum_Anandamide	-0.067 (-0.131, -0.012)	0.008	0.013	-0.063 (-0.135, -0.017)	0.02	0.069	93.4
s_Bacteroides_caecimuris	serum_Alkergot	-0.067 (-0.135, -0.017)	0.012	0.014	-0.047 (-0.106, -0.004)	0.04	0.107	69.6
s_Bacteroides_caecimuris	serum_1,6-Digalloyl-beta-D-glucopyranose	-0.067 (-0.124, -0.015)	0.016	0.017	-0.046 (-0.092, -0.014)	0.012	0.069	68
s_Bacteroides_caecimuris	serum_1-Methylguanosine	-0.067 (-0.13, -0.012)	0.016	0.017	-0.047 (-0.102, 0)	0.048	0.123	69

s_Bacteroides_caecimuris	serum_Retinal	-0.067 (-0.14, -0.012)	0.004	0.011	-0.057 (-0.109, -0.019)	0.016	0.069	85.1
s_Bacteroides_caecimuris	serum_Phytosphingosine-1-P	-0.067 (-0.132, -0.013)	<0.001	<0.001	-0.043 (-0.075, -0.012)	0.004	0.036	63.5
s_Bacteroides_caecimuris	serum_PS(18:1(11Z)/20:0)	-0.067 (-0.131, -0.016)	0.004	0.011	-0.037 (-0.099, -0.001)	0.032	0.096	55.4
s_Bacteroides_caecimuris	serum_LysoPC(17:0/0:0)	-0.067 (-0.136, -0.016)	0.012	0.014	-0.046 (-0.096, -0.009)	0.008	0.052	68.3
s_Bacteroides_caecimuris	serum_Leukotriene C4	-0.067 (-0.134, -0.017)	0.012	0.014	-0.044 (-0.091, -0.013)	0.004	0.036	65.1
s_Bacteroides_caecimuris	serum_gamma-Linolenic acid	-0.067 (-0.138, -0.008)	0.012	0.014	-0.038 (-0.089, -0.007)	0.004	0.036	56.8
s_Alistipes_onderdonkii	intestinal_Cortisone	-0.062 (-0.096, -0.027)	<0.001	<0.001	-0.014 (-0.048, -0.005)	0.016	0.288	22.6
s_Alistipes_onderdonkii	intestinal_Tryptophanamide	-0.062 (-0.098, -0.032)	<0.001	<0.001	-0.017 (-0.056, -0.003)	0.02	0.288	28.2
s_Alistipes_onderdonkii	serum_PC(16:0/20:4(5Z,8Z,11Z,14Z))	-0.062 (-0.092, -0.029)	<0.001	<0.001	-0.035 (-0.078, -0.004)	0.024	0.288	55.8
s_Alistipes_onderdonkii	serum_Sphinganine	-0.062 (-0.097, -0.029)	<0.001	<0.001	-0.031 (-0.056, -0.005)	0.024	0.288	49.9
s_Alistipes_onderdonkii	serum_Anandamide	-0.062 (-0.093, -0.027)	<0.001	<0.001	-0.042 (-0.084, 0)	0.048	0.288	68.2
s_Alistipes_onderdonkii	serum_Retinal	-0.062 (-0.1, -0.029)	<0.001	<0.001	-0.041 (-0.091, -0.007)	0.032	0.288	65.6
s_Alistipes_onderdonkii	serum_LysoPC(17:0/0:0)	-0.062 (-0.091, -0.032)	<0.001	<0.001	-0.03 (-0.065, -0.009)	0.008	0.288	48.5
s_Alistipes_onderdonkii	serum_Leukotriene C4	-0.062 (-0.093, -0.029)	<0.001	<0.001	-0.025 (-0.063, -0.002)	0.032	0.288	40.7
s_Alistipes_onderdonkii	serum_gamma-Linolenic acid	-0.062 (-0.099, -0.028)	<0.001	<0.001	-0.021 (-0.063, 0)	0.044	0.288	34.3
s_Alistipes_shahii	intestinal_Cortisone	-0.081 (-0.131, -0.039)	<0.001	<0.001	-0.018 (-0.057, -0.007)	0.004	0.256	22.5
s_Alistipes_shahii	intestinal_Lauroyl diethanolamide	-0.081 (-0.122, -0.035)	<0.001	<0.001	-0.012 (-0.03, 0)	0.048	0.256	14.7
s_Alistipes_shahii	intestinal_L-Kynurenine	-0.081 (-0.122, -0.036)	<0.001	<0.001	-0.009 (-0.049, 0)	0.048	0.256	11.3
s_Alistipes_shahii	intestinal_Tryptophanamide	-0.081 (-0.127, -0.036)	0.004	0.004	-0.023 (-0.054, -0.002)	0.032	0.256	28.5
s_Alistipes_shahii	serum_Sphinganine	-0.081 (-0.126, -0.035)	<0.001	<0.001	-0.04 (-0.074, -0.005)	0.024	0.256	49.7
s_Alistipes_shahii	serum_Deoxycholic acid	-0.081 (-0.123, -0.034)	<0.001	<0.001	-0.046 (-0.101, -0.002)	0.048	0.256	57.2
s_Alistipes_shahii	serum_Anandamide	-0.081 (-0.124, -0.037)	<0.001	<0.001	-0.056 (-0.103, -0.006)	0.032	0.256	69.3
s_Alistipes_shahii	serum_LysoPC(17:0/0:0)	-0.081 (-0.129, -0.039)	<0.001	<0.001	-0.039 (-0.088, -0.01)	0.016	0.256	48.9
s_Alistipes_shahii	serum_Leukotriene C4	-0.081 (-0.128, -0.033)	0.004	0.004	-0.033 (-0.085, -0.008)	0.024	0.256	41.2

s_Alistipes_shahii	serum_gamma-Linolenic acid	-0.081 (-0.117, -0.037)	<0.001	<0.001	-0.029 (-0.076, -0.002)	0.024	0.256	35.4
s_Alistipes_timonensis	intestinal_Cortisone	-0.067 (-0.104, -0.029)	<0.001	<0.001	-0.015 (-0.048, -0.003)	0.028	0.288	22.6
s_Alistipes_timonensis	intestinal_Lauroyl diethanolamide	-0.067 (-0.109, -0.034)	<0.001	<0.001	-0.01 (-0.025, 0)	0.048	0.288	14.5
s_Alistipes_timonensis	intestinal_Tryptophanamide	-0.067 (-0.11, -0.03)	<0.001	<0.001	-0.019 (-0.055, -0.002)	0.04	0.288	28.2
s_Alistipes_timonensis	serum_PC(16:0/20:4(5Z,8Z,11Z,14Z))	-0.067 (-0.102, -0.033)	<0.001	<0.001	-0.037 (-0.081, -0.003)	0.04	0.288	55.6
s_Alistipes_timonensis	serum_PC(16:0/18:1(9Z))	-0.067 (-0.102, -0.032)	<0.001	<0.001	-0.024 (-0.073, -0.001)	0.04	0.288	36.1
s_Alistipes_timonensis	serum_Anandamide	-0.067 (-0.102, -0.033)	<0.001	<0.001	-0.045 (-0.09, -0.012)	0.02	0.288	67
s_Alistipes_timonensis	serum_Retinal	-0.067 (-0.101, -0.033)	<0.001	<0.001	-0.043 (-0.095, -0.005)	0.028	0.288	63.9
s_Alistipes_timonensis	serum_LysoPC(17:0/0:0)	-0.067 (-0.101, -0.033)	<0.001	<0.001	-0.032 (-0.07, -0.007)	0.02	0.288	48
s_Alistipes_timonensis	serum_Leukotriene C4	-0.067 (-0.104, -0.028)	<0.001	<0.001	-0.027 (-0.069, -0.003)	0.028	0.288	40.6
s_Alistipes_timonensis	serum_gamma-Linolenic acid	-0.067 (-0.106, -0.034)	<0.001	<0.001	-0.023 (-0.062, -0.002)	0.032	0.288	33.8
s_Alistipes_sp_An66	intestinal_Cortisone	-0.059 (-0.093, -0.023)	<0.001	<0.001	-0.013 (-0.045, -0.006)	<0.001	<0.001	22.7
s_Alistipes_sp_An66	intestinal_Lauroyl diethanolamide	-0.059 (-0.089, -0.027)	0.004	0.004	-0.009 (-0.023, 0)	0.044	0.244	14.9
s_Alistipes_sp_An66	intestinal_Tryptophanamide	-0.059 (-0.092, -0.029)	<0.001	<0.001	-0.017 (-0.044, -0.003)	0.02	0.240	28.2
s_Alistipes_sp_An66	serum_PC(16:0/20:4(5Z,8Z,11Z,14Z))	-0.059 (-0.098, -0.031)	<0.001	<0.001	-0.033 (-0.076, -0.007)	0.02	0.240	56.2
s_Alistipes_sp_An66	serum_PC(16:0/18:1(9Z))	-0.059 (-0.089, -0.03)	<0.001	<0.001	-0.022 (-0.068, -0.001)	0.04	0.244	37
s_Alistipes_sp_An66	serum_Sphinganine	-0.059 (-0.09, -0.026)	<0.001	<0.001	-0.029 (-0.053, -0.005)	0.032	0.244	49.9
s_Alistipes_sp_An66	serum_Docosapentaenoic acid (22n-3)	-0.059 (-0.091, -0.03)	<0.001	<0.001	-0.034 (-0.076, -0.004)	0.048	0.247	57
s_Alistipes_sp_An66	serum_Anandamide	-0.059 (-0.096, -0.026)	0.004	0.004	-0.041 (-0.078, -0.011)	0.016	0.240	69.3
s_Alistipes_sp_An66	serum_Retinal	-0.059 (-0.094, -0.029)	<0.001	<0.001	-0.039 (-0.091, -0.006)	0.02	0.240	66.1
s_Alistipes_sp_An66	serum_LysoPC(17:0/0:0)	-0.059 (-0.09, -0.026)	<0.001	<0.001	-0.029 (-0.056, -0.004)	0.024	0.244	48.6
s_Alistipes_sp_An66	serum_Leukotriene C4	-0.059 (-0.095, -0.022)	<0.001	<0.001	-0.024 (-0.061, -0.004)	0.012	0.240	40.3

	s_Alistipes_sp_An66	serum_gamma-Linolenic acid	-0.059 (-0.089, -0.028)	<0.001	<0.001	-0.02 (-0.058, 0)	0.044	0.244	34.2
	s_Duncaniella_muris	intestinal_Cortisone	0.159 (0.017, 0.343)	0.040	0.046	0.104 (0.046, 0.329)	0.008	0.025	65.7
	s_Duncaniella_muris	intestinal_Estrogen	0.159 (0.014, 0.315)	0.028	0.046	0.101 (0.012, 0.248)	0.024	0.062	63.5
	s_Duncaniella_muris	intestinal_L-Kynurenine	0.159 (0.025, 0.319)	0.028	0.046	0.059 (0.006, 0.231)	0.032	0.074	37.3
	s_Duncaniella_muris	serum_(±)-Tryptophan	0.159 (0.029, 0.328)	0.020	0.046	0.151 (0.039, 0.302)	0.012	0.035	94.9
	s_Duncaniella_muris	serum_PS(18:1(11Z)/20:0)	0.159 (0.021, 0.317)	0.024	0.046	0.14 (0.016, 0.402)	0.04	0.090	88.2
	s_Duncaniella_muris	serum_Glucosamine 6-phosphate	0.159 (0.013, 0.321)	0.016	0.046	0.152 (0.019, 0.361)	0.032	0.074	95.6
	s_Duncaniella_muris	serum_Leukotriene C4	0.159 (0.024, 0.318)	0.020	0.046	0.125 (0.01, 0.449)	0.012	0.035	78.6
	s_Duncaniella_muris	serum_gamma-Linolenic acid	0.159 (0.014, 0.319)	0.028	0.046	0.13 (0.02, 0.345)	0.028	0.070	81.6
brain_L-Acetylcarnitine	f_Erysipelotrichaceae	serum_Leukotriene C4	-0.24 (-0.53, -0.009)	0.044	0.082	-0.182 (-0.332, -0.063)	<0.001	<0.001	75.8
	s_Erysipelotrichaceae_bacterium	intestinal_Tolmetin	-0.304 (-0.612, -0.057)	0.020	0.034	-0.171 (-0.341, -0.013)	0.04	0.264	56.4
	s_Erysipelotrichaceae_bacterium	intestinal_Ubiquinone-2	-0.304 (-0.585, -0.07)	0.012	0.034	-0.204 (-0.5, -0.022)	0.036	0.264	67.1
	s_Erysipelotrichaceae_bacterium	serum_Dimyristoylphosphatidylcholine, DL	-0.304 (-0.594, -0.066)	0.016	0.034	-0.206 (-0.494, -0.005)	0.044	0.264	67.7
	s_Erysipelotrichaceae_bacterium	serum_Leukotriene C4	-0.304 (-0.591, -0.066)	0.020	0.034	-0.211 (-0.403, -0.058)	0.004	0.144	69.3
	s_Erysipelotrichaceae_bacterium	serum_gamma-Linolenic acid	-0.304 (-0.586, -0.064)	0.012	0.034	-0.117 (-0.355, -0.005)	0.036	0.264	38.5
	s_Muribaculaceae_bacterium_Isolate_037_Harlan	intestinal_Cer(d18:1/16:0)	0.939 (0.289, 1.756)	0.004	0.007	0.832 (0.171, 1.869)	0.008	0.576	88.6
	s_Muribaculaceae_bacterium_Isolate_037_Harlan	intestinal_Tolmetin	0.939 (0.256, 1.728)	0.012	0.012	0.383 (0.018, 0.856)	0.028	0.763	40.8
	s_Muribaculaceae_bacterium_Isolate_037_Harlan	serum_Leukotriene C4	0.939 (0.291, 1.664)	0.008	0.009	0.47 (0.027, 1.433)	0.048	0.763	50.1
	s_Muribaculaceae_bacterium_Isolate_037_Harlan	serum_gamma-Linolenic acid	0.939 (0.278, 1.722)	0.008	0.009	0.458 (0.025, 1.587)	0.032	0.763	48.8

	g_Alistipes	serum_Dimyristoylphosphatidylcholine, DL	0.295 (0.008, 0.569)	0.044	0.085	0.139 (0.01, 0.374)	0.04	0.720	47
	s_Bacteroidales_bacterium	intestinal_Cer(d18:1/16:0)	0.915 (0.271, 1.618)	0.008	0.008	0.822 (0.245, 1.987)	0.004	0.288	89.9
	s_Bacteroidales_bacterium	intestinal_Tolmetin	0.915 (0.37, 1.782)	<0.001	<0.001	0.384 (0.043, 0.865)	0.02	0.480	42
	s_Bacteroidales_bacterium	serum_Leukotriene C4	0.915 (0.353, 1.687)	0.008	0.008	0.468 (0.101, 1.254)	0.012	0.432	51.2
	o_Coriobacteriales	intestinal_Tolmetin	-0.349 (-0.531, -0.195)	<0.001	<0.001	-0.125 (-0.318, -0.003)	0.044	0.864	35.7
	f_Odoribacteraceae	intestinal_Cer(d18:1/16:0)	0.437 (0.065, 0.736)	0.032	0.038	0.27 (0.06, 0.482)	0.02	0.360	61.7
	f_Odoribacteraceae	serum_Dimyristoylphosphatidylcholine, DL	0.437 (0.046, 0.72)	0.028	0.038	0.178 (0.015, 0.429)	0.036	0.432	40.8
	f_Odoribacteraceae	serum_Leukotriene C4	0.437 (0.033, 0.756)	0.036	0.039	0.211 (0.017, 0.396)	0.02	0.360	48.2
	f_Odoribacteraceae	serum_gamma-Linolenic acid	0.437 (0.061, 0.737)	0.032	0.038	0.138 (0.001, 0.373)	0.048	0.432	31.6
	f_Coriobacteriaceae	intestinal_Tolmetin	-0.361 (-0.523, -0.185)	<0.001	<0.001	-0.131 (-0.331, 0)	0.048	0.967	36.1
	g_Odoribacter	intestinal_Cer(d18:1/16:0)	0.428 (0.045, 0.747)	0.032	0.047	0.267 (0.021, 0.48)	0.032	0.518	62.4
	g_Odoribacter	serum_Dimyristoylphosphatidylcholine, DL	0.428 (0.026, 0.717)	0.040	0.047	0.177 (0.021, 0.438)	0.024	0.518	41.4
	g_Odoribacter	serum_Leukotriene C4	0.428 (0.015, 0.744)	0.044	0.047	0.211 (0.021, 0.404)	0.036	0.518	49.3
	s_Alistipes_sp_	intestinal_Tolmetin	0.222 (0.017, 0.431)	0.044	0.052	0.12 (0.018, 0.292)	0.024	0.624	54
	s_Alistipes_sp_	serum_Leukotriene C4	0.222 (0.029, 0.42)	0.032	0.050	0.153 (0.009, 0.312)	0.04	0.624	68.9
	s_Bacteroides_caecimuris	intestinal_Tolmetin	0.474 (0.132, 0.813)	0.008	0.012	0.198 (0.016, 0.449)	0.036	0.480	41.7
	s_Bacteroides_caecimuris	serum_Leukotriene C4	0.474 (0.122, 0.793)	0.004	0.012	0.217 (0.033, 0.433)	0.024	0.432	45.8
	s_Bacteroides_caecimuris	serum_gamma-Linolenic acid	0.474 (0.115, 0.79)	0.020	0.020	0.179 (0.017, 0.486)	0.024	0.432	37.9
brain_Isoleucyl-Valine	c_Erysipelotrichia	intestinal_Estrogen	0.345 (0.127, 0.558)	0.004	0.004	0.196 (0.014, 0.446)	0.044	0.684	56.8
	c_Erysipelotrichia	intestinal_Staurosporine	0.345 (0.152, 0.575)	<0.001	<0.001	0.117 (0.012, 0.235)	0.024	0.684	34
	o_Erysipelotrichales	intestinal_Anandamide	0.345 (0.138, 0.536)	0.004	0.004	0.107 (0.003, 0.251)	0.048	0.554	31
	o_Erysipelotrichales	intestinal_Estrogen	0.345 (0.155, 0.565)	<0.001	<0.001	0.196 (0.018, 0.449)	0.04	0.554	56.8
	o_Erysipelotrichales	intestinal_Staurosporine	0.345 (0.143, 0.531)	<0.001	<0.001	0.117 (0.004, 0.216)	0.044	0.554	34

s_Erysipelotrichaceae_bacterium	intestinal_Anandamide	0.339 (0.148, 0.554)	<0.001	<0.001	0.107 (0.018, 0.273)	0.004	0.288	31.6
s_Erysipelotrichaceae_bacterium	intestinal_Estrogen	0.339 (0.15, 0.55)	<0.001	<0.001	0.215 (0.024, 0.492)	0.04	0.518	63.5
s_Erysipelotrichaceae_bacterium	serum_Sphingosine	0.339 (0.152, 0.552)	<0.001	<0.001	0.247 (0.058, 0.476)	0.012	0.432	72.8
f_Rikenellaceae	intestinal_3a,6a,7b-Trihydroxy-5b-cholanoic acid	-0.426 (-0.645, -0.247)	<0.001	<0.001	-0.097 (-0.237, -0.004)	0.044	0.813	22.7
f_Rikenellaceae	intestinal_Estrogen	-0.426 (-0.664, -0.251)	<0.001	<0.001	-0.178 (-0.303, -0.024)	0.04	0.813	41.7
f_Rikenellaceae	intestinal_Staurosporine	-0.426 (-0.669, -0.247)	<0.001	<0.001	-0.1 (-0.252, -0.015)	0.016	0.813	23.4
g_Alistipes	intestinal_3a,6a,7b-Trihydroxy-5b-cholanoic acid	-0.352 (-0.538, -0.196)	<0.001	<0.001	-0.081 (-0.19, -0.01)	0.008	0.576	23
g_Alistipes	intestinal_Estrogen	-0.352 (-0.512, -0.172)	<0.001	<0.001	-0.162 (-0.28, -0.008)	0.044	0.816	45.8
g_Alistipes	intestinal_Staurosporine	-0.352 (-0.539, -0.182)	<0.001	<0.001	-0.083 (-0.21, -0.003)	0.04	0.816	23.6
s_Prevotella_sp_PMUR	intestinal_2,3-Diphosphoglyceric acid	-0.325 (-0.494, -0.092)	0.012	0.018	-0.105 (-0.278, -0.011)	0.02	0.288	32.3
s_Prevotella_sp_PMUR	intestinal_Estrogen	-0.325 (-0.478, -0.1)	0.008	0.018	-0.221 (-0.417, -0.081)	<0.001	<0.001	68
s_Prevotella_sp_PMUR	intestinal_Indoxyl sulfate	-0.325 (-0.478, -0.086)	0.012	0.018	-0.094 (-0.257, -0.008)	0.024	0.288	29
s_Prevotella_sp_PMUR	serum_Arachidoyl Ethanolamide	-0.325 (-0.483, -0.058)	0.020	0.021	-0.109 (-0.254, -0.007)	0.04	0.288	33.5
s_Prevotella_sp_PMUR	serum_Glycocholic acid	-0.325 (-0.487, -0.079)	0.008	0.018	-0.113 (-0.217, -0.004)	0.044	0.288	34.8
s_Prevotella_sp_PMUR	serum_Sphingosine	-0.325 (-0.479, -0.094)	0.004	0.018	-0.216 (-0.399, -0.087)	0.012	0.288	66.4
s_Prevotella_sp_PMUR	serum_Alkergot	-0.325 (-0.486, -0.098)	0.008	0.018	-0.12 (-0.239, -0.009)	0.032	0.288	36.9
s_Prevotella_sp_PMUR	serum_1,6-Digalloyl-beta-D-glucopyranose	-0.325 (-0.477, -0.074)	0.012	0.018	-0.132 (-0.287, -0.034)	0.016	0.288	40.7
g_Duncaniella	intestinal_Estrogen	0.8 (0.473, 1.271)	<0.001	<0.001	0.309 (0.12, 0.665)	<0.001	<0.001	38.6
g_Duncaniella	intestinal_Staurosporine	0.8 (0.501, 1.275)	<0.001	<0.001	0.24 (0.004, 0.703)	0.036	0.379	30
g_Duncaniella	intestinal_Indoxyl sulfate	0.8 (0.512, 1.252)	<0.001	<0.001	0.175 (0.003, 0.528)	0.044	0.379	21.9
g_Duncaniella	intestinal_9-Hpode	0.8 (0.524, 1.252)	<0.001	<0.001	0.162 (0.012, 0.481)	0.028	0.379	20.2
g_Duncaniella	serum_(±)-Tryptophan	0.8 (0.49, 1.214)	<0.001	<0.001	0.236 (0.035, 0.695)	0.016	0.379	29.5

g_Duncaniella	serum_Sphingosine	0.8 (0.509, 1.195)	<0.001	<0.001	0.576 (0.024, 1.317)	0.044	0.379	72
g_Duncaniella	serum_1,6-Digalloyl-beta-D-glucopyranose	0.8 (0.514, 1.249)	<0.001	<0.001	0.261 (0.089, 0.665)	0.004	0.144	32.6
g_Heminiphilus	intestinal_Anandamide	-0.443 (-0.739, -0.158)	<0.001	<0.001	-0.124 (-0.342, -0.007)	0.036	0.220	28.1
g_Heminiphilus	intestinal_Estrogen	-0.443 (-0.701, -0.142)	<0.001	<0.001	-0.2 (-0.39, -0.045)	0.016	0.220	45.1
g_Heminiphilus	intestinal_Tryptophanamide	-0.443 (-0.727, -0.168)	0.004	0.005	-0.096 (-0.219, -0.005)	0.036	0.220	21.6
g_Heminiphilus	serum_(±)-Tryptophan	-0.443 (-0.733, -0.163)	0.004	0.005	-0.162 (-0.405, -0.019)	0.028	0.220	36.5
g_Heminiphilus	serum_Arachidoyl Ethanolamide	-0.443 (-0.708, -0.166)	0.004	0.005	-0.116 (-0.234, -0.011)	0.028	0.220	26.1
g_Heminiphilus	serum_Phenyllactic acid	-0.443 (-0.729, -0.141)	0.004	0.005	-0.136 (-0.292, -0.018)	0.012	0.220	30.7
g_Heminiphilus	serum_Esterase	-0.443 (-0.729, -0.154)	<0.001	<0.001	-0.081 (-0.206, -0.003)	0.04	0.220	18.4
g_Heminiphilus	serum_Palmitoylethanolamide	-0.443 (-0.723, -0.15)	0.004	0.005	-0.106 (-0.241, -0.008)	0.016	0.220	23.9
g_Heminiphilus	serum_Glycocholic acid	-0.443 (-0.744, -0.195)	<0.001	<0.001	-0.111 (-0.226, -0.019)	0.024	0.220	25
g_Heminiphilus	serum_Sphingosine	-0.443 (-0.71, -0.186)	0.004	0.005	-0.299 (-0.515, -0.108)	<0.001	<0.001	67.4
g_Heminiphilus	serum_PC(16:0/20:4(5Z,8Z,11Z,14Z))	-0.443 (-0.728, -0.161)	0.004	0.005	-0.083 (-0.186, -0.003)	0.04	0.220	18.6
g_Heminiphilus	serum_Deoxycholic acid	-0.443 (-0.761, -0.186)	<0.001	<0.001	-0.095 (-0.191, -0.006)	0.044	0.220	21.5
g_Heminiphilus	serum_Docosapentaenoic acid (22n-3)	-0.443 (-0.728, -0.145)	<0.001	<0.001	-0.093 (-0.22, -0.001)	0.048	0.220	20.9
g_Heminiphilus	serum_Alkergot	-0.443 (-0.75, -0.171)	0.004	0.005	-0.13 (-0.258, -0.016)	0.024	0.220	29.5
g_Heminiphilus	serum_1,6-Digalloyl-beta-D-glucopyranose	-0.443 (-0.721, -0.168)	0.004	0.005	-0.117 (-0.255, -0.008)	0.028	0.220	26.4
s_Heminiphilus_faecis	intestinal_alpha-Linolenic acid	-0.443 (-0.694, -0.123)	<0.001	<0.001	-0.079 (-0.509, -0.001)	0.048	0.220	17.8
s_Heminiphilus_faecis	intestinal_Anandamide	-0.443 (-0.739, -0.163)	0.004	0.006	-0.124 (-0.309, -0.014)	0.024	0.216	28.1
s_Heminiphilus_faecis	intestinal_Estrogen	-0.443 (-0.715, -0.16)	0.004	0.006	-0.2 (-0.463, -0.053)	0.016	0.216	45.1
s_Heminiphilus_faecis	intestinal_Tryptophanamide	-0.443 (-0.72, -0.127)	0.016	0.016	-0.096 (-0.236, -0.001)	0.048	0.220	21.6
s_Heminiphilus_faecis	serum_(±)-Tryptophan	-0.443 (-0.74, -0.148)	0.008	0.009	-0.162 (-0.422, -0.023)	0.004	0.216	36.5

s_Heminiphilus_faecis	serum_Arachidoyl Ethanolamide	-0.443 (-0.73, -0.131)	0.004	0.006	-0.116 (-0.271, -0.019)	0.024	0.216	26.1
s_Heminiphilus_faecis	serum_Phenyllactic acid	-0.443 (-0.741, -0.182)	0.004	0.006	-0.136 (-0.289, -0.03)	0.016	0.216	30.7
s_Heminiphilus_faecis	serum_Heptadecanoic acid	-0.443 (-0.734, -0.144)	<0.001	<0.001	-0.111 (-0.24, -0.006)	0.036	0.220	25.1
s_Heminiphilus_faecis	serum_Palmitoylethanolamide	-0.443 (-0.732, -0.135)	0.008	0.009	-0.106 (-0.234, -0.014)	0.032	0.220	23.9
s_Heminiphilus_faecis	serum_Glycocholic acid	-0.443 (-0.715, -0.131)	<0.001	<0.001	-0.111 (-0.225, -0.02)	0.02	0.216	25
s_Heminiphilus_faecis	serum_Sphingosine	-0.443 (-0.725, -0.123)	0.004	0.006	-0.299 (-0.518, -0.092)	0.008	0.216	67.4
s_Heminiphilus_faecis	serum_Deoxycholic acid	-0.443 (-0.715, -0.111)	0.004	0.006	-0.095 (-0.195, -0.002)	0.04	0.220	21.5
s_Heminiphilus_faecis	serum_Alkergot	-0.443 (-0.746, -0.176)	0.004	0.006	-0.13 (-0.265, -0.02)	0.024	0.216	29.5
s_Heminiphilus_faecis	serum_1,6-Digalloyl-beta-D-glucopyranose	-0.443 (-0.721, -0.149)	<0.001	<0.001	-0.117 (-0.239, -0.003)	0.044	0.220	26.4
s_Heminiphilus_faecis	serum_1-Methylguanosine	-0.443 (-0.717, -0.172)	<0.001	<0.001	-0.105 (-0.247, -0.002)	0.044	0.220	23.8
s_Duncaniella_dubosii	intestinal_Anandamide	0.585 (0.375, 0.86)	<0.001	<0.001	0.108 (0.004, 0.344)	0.04	0.470	18.4
s_Duncaniella_dubosii	intestinal_Estrogen	0.585 (0.388, 0.864)	<0.001	<0.001	0.198 (0.069, 0.38)	0.004	0.144	33.9
s_Duncaniella_dubosii	intestinal_Stearic acid	0.585 (0.373, 0.889)	<0.001	<0.001	0.104 (0.001, 0.329)	0.044	0.470	17.8
s_Duncaniella_dubosii	serum_1,6-Digalloyl-beta-D-glucopyranose	0.585 (0.399, 0.871)	<0.001	<0.001	0.157 (0.051, 0.338)	0.004	0.144	26.7
g_Allobaculum	intestinal_Estrogen	0.129 (0.077, 0.176)	<0.001	<0.001	0.043 (0.008, 0.083)	0.032	0.939	33.2
g_Allobaculum	serum_1,6-Digalloyl-beta-D-glucopyranose	0.129 (0.085, 0.176)	<0.001	<0.001	0.033 (0.005, 0.082)	0.024	0.939	25.5
s_Allobaculum_sp_539	intestinal_Estrogen	0.101 (0.057, 0.144)	<0.001	<0.001	0.037 (0.001, 0.069)	0.044	0.969	36.8
s_Allobaculum_sp_539	serum_1,6-Digalloyl-beta-D-glucopyranose	0.101 (0.054, 0.14)	<0.001	<0.001	0.029 (0.002, 0.062)	0.036	0.969	28.6
s_Muribaculaceae_bacterium_Isolate_080_Janvier	intestinal_Anandamide	0.687 (0.423, 1.01)	<0.001	<0.001	0.121 (0.001, 0.447)	0.048	0.457	17.6
s_Muribaculaceae_bacterium_Isolate_080_Janvier	intestinal_Estrogen	0.687 (0.438, 1.024)	<0.001	<0.001	0.242 (0.075, 0.508)	0.008	0.288	35.2
s_Muribaculaceae_bacterium_Isolate_080_Janvier	intestinal_Stearic acid	0.687 (0.458, 1.047)	<0.001	<0.001	0.119 (0.002, 0.35)	0.04	0.457	17.4
s_Muribaculaceae_bacterium_Isolate_080_Janvier	serum_1,6-Digalloyl-beta-D-glucopyranose	0.687 (0.435, 1.035)	<0.001	<0.001	0.193 (0.061, 0.398)	0.004	0.288	28

s_Muribaculaceae_bacterium_Isolate_002_NCI	serum_Sphingosine	-0.602 (-1.16, -0.319)	<0.001	<0.001	-0.33 (-0.716, -0.021)	0.044	0.662	54.8
s_Muribaculaceae_bacterium_Isolate_002_NCI	serum_1,6-Digalloyl-beta-D-glucopyranose	-0.602 (-1.149, -0.296)	<0.001	<0.001	-0.221 (-0.43, -0.036)	0.02	0.662	36.7
f_Odoribacteraceae	intestinal_2,3-Diphosphoglyceric acid	-0.381 (-0.674, -0.135)	0.004	0.005	-0.117 (-0.31, -0.012)	0.044	0.403	30.7
f_Odoribacteraceae	intestinal_Estrogen	-0.381 (-0.66, -0.123)	<0.001	<0.001	-0.222 (-0.488, -0.083)	0.008	0.192	58.4
f_Odoribacteraceae	intestinal_9-Hpode	-0.381 (-0.653, -0.139)	<0.001	<0.001	-0.12 (-0.356, -0.013)	0.02	0.360	31.5
f_Odoribacteraceae	serum_Sphingosine	-0.381 (-0.705, -0.14)	<0.001	<0.001	-0.362 (-0.708, -0.129)	0.008	0.192	95.2
f_Odoribacteraceae	serum_1,6-Digalloyl-beta-D-glucopyranose	-0.381 (-0.656, -0.118)	<0.001	<0.001	-0.192 (-0.358, -0.057)	0.008	0.192	50.4
s_Bacteroidales_bacterium_55_9	intestinal_Estrogen	-0.169 (-0.294, -0.069)	<0.001	<0.001	-0.109 (-0.218, -0.036)	0.012	0.432	64.5
s_Bacteroidales_bacterium_55_9	serum_1,6-Digalloyl-beta-D-glucopyranose	-0.169 (-0.298, -0.066)	<0.001	<0.001	-0.12 (-0.216, -0.008)	0.036	0.656	70.8
s_Coriobacteriaceae_bacterium	intestinal_2,3-Diphosphoglyceric acid	0.089 (0.026, 0.168)	0.008	0.008	0.026 (0.003, 0.065)	0.028	0.384	29.4
s_Coriobacteriaceae_bacterium	intestinal_alpha-Linolenic acid	0.089 (0.025, 0.175)	0.012	0.012	0.022 (0.002, 0.104)	0.044	0.384	25.2
s_Coriobacteriaceae_bacterium	intestinal_Anandamide	0.089 (0.034, 0.172)	0.004	0.006	0.052 (0.001, 0.105)	0.04	0.384	58.3
s_Coriobacteriaceae_bacterium	intestinal_Estrogen	0.089 (0.027, 0.173)	<0.001	<0.001	0.07 (0.03, 0.15)	0.008	0.192	78.6
s_Coriobacteriaceae_bacterium	intestinal_N-Acetyl-L-phenylalanine	0.089 (0.031, 0.167)	<0.001	<0.001	0.034 (0.002, 0.081)	0.032	0.384	37.9
s_Alistipes_senegalensis	intestinal_2,3-Diphosphoglyceric acid	-0.39 (-0.565, -0.221)	<0.001	<0.001	-0.076 (-0.185, -0.002)	0.048	0.691	19.6
s_Alistipes_senegalensis	intestinal_3a,6a,7b-Trihydroxy-5b-cholanoic acid	-0.39 (-0.595, -0.232)	<0.001	<0.001	-0.088 (-0.233, -0.004)	0.04	0.691	22.6
s_Alistipes_senegalensis	intestinal_Estrogen	-0.39 (-0.582, -0.233)	<0.001	<0.001	-0.167 (-0.293, -0.007)	0.048	0.691	42.8
s_Alistipes_senegalensis	intestinal_Staurosporine	-0.39 (-0.601, -0.218)	<0.001	<0.001	-0.091 (-0.21, -0.005)	0.032	0.691	23.3

s_Alistipes_finegoldii	intestinal_3a,6a,7b-Trihydroxy-5b-cholanoic acid	-0.35 (-0.519, -0.205)	<0.001	<0.001	-0.081 (-0.203, -0.003)	0.036	0.758	23
s_Alistipes_finegoldii	intestinal_Estrogen	-0.35 (-0.54, -0.199)	<0.001	<0.001	-0.152 (-0.258, -0.026)	0.028	0.758	43.5
s_Alistipes_finegoldii	intestinal_Staurosporine	-0.35 (-0.537, -0.195)	<0.001	<0.001	-0.083 (-0.21, -0.003)	0.04	0.758	23.7
g_Odoribacter	intestinal_2,3-Diphosphoglyceric acid	-0.393 (-0.675, -0.153)	<0.001	<0.001	-0.116 (-0.35, -0.012)	0.024	0.432	29.5
g_Odoribacter	intestinal_Estrogen	-0.393 (-0.653, -0.168)	0.004	0.004	-0.219 (-0.47, -0.085)	0.004	0.144	55.6
g_Odoribacter	serum_Sphingosine	-0.393 (-0.671, -0.136)	<0.001	<0.001	-0.367 (-0.765, -0.119)	0.004	0.144	93.3
g_Odoribacter	serum_Alkergot	-0.393 (-0.663, -0.151)	<0.001	<0.001	-0.163 (-0.363, -0.004)	0.048	0.432	41.4
g_Odoribacter	serum_1,6-Digalloyl-beta-D-glucopyranose	-0.393 (-0.698, -0.166)	<0.001	<0.001	-0.189 (-0.359, -0.065)	0.008	0.192	48
s_Alistipes_sp_58_9_plus	intestinal_alpha-Linolenic acid	-0.168 (-0.284, -0.071)	<0.001	<0.001	-0.035 (-0.184, -0.001)	0.048	0.662	20.8
s_Alistipes_sp_58_9_plus	intestinal_Estrogen	-0.168 (-0.293, -0.069)	0.004	0.004	-0.101 (-0.196, -0.037)	0.012	0.432	60.3
s_Alistipes_sp_58_9_plus	serum_1,6-Digalloyl-beta-D-glucopyranose	-0.168 (-0.283, -0.078)	<0.001	<0.001	-0.105 (-0.195, -0.014)	0.024	0.576	62.8
s_Alistipes_sp_	intestinal_2,3-Diphosphoglyceric acid	-0.224 (-0.368, -0.11)	<0.001	<0.001	-0.057 (-0.118, -0.002)	0.044	0.672	25.5
s_Alistipes_sp_	intestinal_Estrogen	-0.224 (-0.368, -0.099)	<0.001	<0.001	-0.129 (-0.238, -0.047)	0.008	0.432	57.6
s_Bacteroides_caecimuris	intestinal_2,3-Diphosphoglyceric acid	-0.378 (-0.707, -0.101)	0.004	0.004	-0.109 (-0.275, -0.02)	0.024	0.346	28.9
s_Bacteroides_caecimuris	intestinal_alpha-Linolenic acid	-0.378 (-0.663, -0.135)	<0.001	<0.001	-0.086 (-0.443, -0.017)	0.016	0.288	22.7
s_Bacteroides_caecimuris	intestinal_Estrogen	-0.378 (-0.722, -0.134)	0.004	0.004	-0.261 (-0.609, -0.116)	0.004	0.144	68.9
s_Bacteroides_caecimuris	intestinal_N-Acetyl-L-phenylalanine	-0.378 (-0.679, -0.13)	0.004	0.004	-0.108 (-0.268, -0.008)	0.044	0.403	28.5
s_Bacteroides_caecimuris	serum_1,6-Digalloyl-beta-D-glucopyranose	-0.378 (-0.695, -0.142)	0.004	0.004	-0.252 (-0.519, -0.084)	0.012	0.288	66.8
s_Alistipes_onderdonkii	intestinal_3a,6a,7b-Trihydroxy-5b-cholanoic acid	-0.339 (-0.508, -0.196)	<0.001	<0.001	-0.078 (-0.187, -0.003)	0.04	0.757	23

	s_Alistipes_onderdonkii	intestinal_Estrogen	-0.339 (-0.522, -0.189)	<0.001	<0.001	-0.146 (-0.267, -0.017)	0.036	0.757	43.2
	s_Alistipes_onderdonkii	intestinal_Staurosporine	-0.339 (-0.528, -0.208)	<0.001	<0.001	-0.08 (-0.19, -0.005)	0.036	0.757	23.6
	s_Alistipes_shahii	intestinal_3a,6a,7b-Trihydroxy-5b-cholanoic acid	-0.436 (-0.662, -0.228)	<0.001	<0.001	-0.102 (-0.258, -0.003)	0.044	0.672	23.4
	s_Alistipes_shahii	intestinal_Estrogen	-0.436 (-0.686, -0.234)	<0.001	<0.001	-0.196 (-0.329, -0.046)	0.02	0.672	44.8
	s_Alistipes_shahii	intestinal_Staurosporine	-0.436 (-0.668, -0.261)	<0.001	<0.001	-0.105 (-0.277, -0.002)	0.032	0.672	24.1
	s_Alistipes_timonensis	intestinal_3a,6a,7b-Trihydroxy-5b-cholanoic acid	-0.368 (-0.566, -0.215)	<0.001	<0.001	-0.084 (-0.23, -0.01)	0.02	0.720	22.7
	s_Alistipes_timonensis	intestinal_Estrogen	-0.368 (-0.572, -0.196)	<0.001	<0.001	-0.158 (-0.296, -0.034)	0.032	0.720	43
	s_Alistipes_timonensis	intestinal_Staurosporine	-0.368 (-0.551, -0.202)	<0.001	<0.001	-0.086 (-0.229, -0.006)	0.032	0.720	23.5
	s_Alistipes_sp_An66	intestinal_Estrogen	-0.327 (-0.527, -0.185)	<0.001	<0.001	-0.138 (-0.249, -0.017)	0.044	0.756	42
	s_Alistipes_sp_An66	intestinal_Staurosporine	-0.327 (-0.502, -0.196)	<0.001	<0.001	-0.077 (-0.193, -0.001)	0.044	0.756	23.6
	s_Duncaniella_muris	intestinal_Estrogen	1.259 (0.538, 1.948)	<0.001	<0.001	0.754 (0.22, 1.358)	0.008	0.288	59.9
	s_Duncaniella_muris	serum_1,6-Digalloyl-beta-D-glucopyranose	1.259 (0.398, 2.015)	0.008	0.009	0.828 (0.214, 2.16)	0.008	0.288	65.8
brain_Cytidine	f_Rikenellaceae	intestinal_2,3-Diphosphoglyceric acid	-0.618 (-1.021, -0.295)	<0.001	<0.001	-0.151 (-0.346, -0.01)	0.04	0.360	24.5
	f_Rikenellaceae	intestinal_Linoleoyl ethanolamide	-0.618 (-1.009, -0.264)	<0.001	<0.001	-0.246 (-0.573, -0.012)	0.04	0.360	39.7
	f_Rikenellaceae	intestinal_N2-gamma-Glutamylglutamine	-0.618 (-1.03, -0.253)	<0.001	<0.001	-0.187 (-0.445, -0.007)	0.048	0.384	30.3
	g_Alistipes	intestinal_2,3-Diphosphoglyceric acid	-0.515 (-0.832, -0.207)	<0.001	<0.001	-0.132 (-0.289, -0.023)	0.016	0.192	25.6
	g_Alistipes	intestinal_Linoleoyl ethanolamide	-0.515 (-0.848, -0.185)	0.004	0.004	-0.216 (-0.496, -0.039)	0.016	0.192	41.9
	g_Alistipes	intestinal_N2-gamma-Glutamylglutamine	-0.515 (-0.857, -0.25)	<0.001	<0.001	-0.16 (-0.344, -0.013)	0.036	0.288	31.1
	s_Prevotella_sp_PMUR	intestinal_2,3-Diphosphoglyceric acid	-0.439 (-0.743, -0.019)	0.048	0.048	-0.206 (-0.478, -0.019)	0.036	0.554	46.9
	s_Prevotella_sp_PMUR	intestinal_Estrogen	-0.439 (-0.774, -0.086)	0.016	0.031	-0.29 (-0.613, -0.024)	0.028	0.554	66.1

s_Prevotella_sp_PMUR	serum_Sphingosine	-0.439 (-0.75, -0.081)	0.012	0.031	-0.251 (-0.584, -0.054)	0.02	0.554	57.2
g_Duncaniella	intestinal_2,3-Diphosphoglyceric acid	1.036 (0.488, 1.739)	<0.001	<0.001	0.304 (0.02, 0.881)	0.024	0.754	29.3
g_Duncaniella	intestinal_Estrogen	1.036 (0.42, 1.743)	0.004	0.004	0.422 (0.004, 0.829)	0.048	0.754	40.8
g_Duncaniella	intestinal_N2-gamma-Glutamylglutamine	1.036 (0.477, 1.706)	0.004	0.004	0.361 (0.058, 0.744)	0.036	0.754	34.9
s_Duncaniella_dubosii	intestinal_2,3-Diphosphoglyceric acid	0.663 (0.304, 1.149)	<0.001	<0.001	0.217 (0.009, 0.599)	0.044	0.695	32.7
s_Duncaniella_dubosii	intestinal_Estrogen	0.663 (0.24, 1.105)	0.016	0.016	0.314 (0.02, 0.647)	0.036	0.695	47.4
s_Duncaniella_dubosii	intestinal_N2-gamma-Glutamylglutamine	0.663 (0.245, 1.114)	0.004	0.005	0.274 (0.017, 0.579)	0.028	0.695	41.3
g_Faecalibaculum	intestinal_N2-gamma-Glutamylglutamine	0.215 (0.052, 0.429)	0.008	0.025	0.124 (0.003, 0.257)	0.048	0.657	57.5
s_Faecalibaculum_rodentium	intestinal_2,3-Diphosphoglyceric acid	0.215 (0.043, 0.431)	0.004	0.023	0.109 (0.006, 0.217)	0.036	0.643	50.6
g_Allobaculum	intestinal_2,3-Diphosphoglyceric acid	0.179 (0.087, 0.263)	<0.001	<0.001	0.046 (0.002, 0.11)	0.036	0.634	25.6
g_Allobaculum	intestinal_Linoleoyl ethanolamide	0.179 (0.081, 0.272)	0.004	0.004	0.057 (0.003, 0.162)	0.028	0.634	32.1
g_Allobaculum	intestinal_N2-gamma-Glutamylglutamine	0.179 (0.094, 0.263)	<0.001	<0.001	0.054 (0.001, 0.121)	0.044	0.634	30.4
s_Allobaculum_sp_539	intestinal_2,3-Diphosphoglyceric acid	0.141 (0.061, 0.219)	<0.001	<0.001	0.038 (0.003, 0.09)	0.032	0.864	26.8
s_Allobaculum_sp_539	intestinal_Linoleoyl ethanolamide	0.141 (0.063, 0.222)	0.008	0.008	0.05 (0, 0.123)	0.048	0.864	35.4
s_Bacteroidales_bacterium	intestinal_Estrogen	-0.777 (-1.834, -0.009)	0.048	0.089	-0.623 (-1.392, -0.184)	0.024	0.252	80.1
s_Bacteroidales_bacterium	serum_Deoxycholic acid	-0.777 (-1.855, -0.014)	0.048	0.089	-0.55 (-1.563, -0.034)	0.036	0.252	70.7
s_Bacteroidales_bacterium	serum_Docosapentaenoic acid (22n-3)	-0.777 (-1.927, -0.023)	0.044	0.089	-0.617 (-1.92, -0.056)	0.032	0.252	79.4

s_Bacteroidales_bacterium	serum_1-Methylguanosine	-0.777 (-1.759, -0.063)	0.024	0.089	-0.662 (-1.842, -0.059)	0.024	0.252	85.1
s_Muribaculaceae_bacterium_Isolate_080_Janvier	intestinal_Estrogen	0.796 (0.33, 1.31)	0.004	0.004	0.373 (0.039, 0.798)	0.028	0.704	46.8
s_Muribaculaceae_bacterium_Isolate_080_Janvier	intestinal_N2-gamma-Glutamylglutamine	0.796 (0.357, 1.389)	<0.001	<0.001	0.322 (0.07, 0.66)	0.024	0.704	40.5
s_Muribaculaceae_bacterium_Isolate_080_Janvier	serum_1,6-Digalloyl-beta-D-glucopyranose	0.796 (0.32, 1.365)	<0.001	<0.001	0.261 (0.008, 0.883)	0.036	0.704	32.7
f_Odoribacteraceae	intestinal_2,3-Diphosphoglyceric acid	-0.62 (-1.131, -0.133)	0.012	0.014	-0.194 (-0.478, -0.016)	0.032	0.765	31.2
f_Odoribacteraceae	intestinal_Estrogen	-0.62 (-1.14, -0.169)	0.008	0.013	-0.249 (-0.586, -0.006)	0.048	0.765	40.1
f_Odoribacteraceae	intestinal_Linoleoyl ethanolamide	-0.62 (-1.171, -0.138)	0.016	0.017	-0.233 (-0.462, -0.004)	0.044	0.765	37.6
s_Bacteroidales_bacterium_55_9	intestinal_2,3-Diphosphoglyceric acid	-0.305 (-0.487, -0.09)	<0.001	<0.001	-0.073 (-0.168, -0.009)	0.028	0.224	24
s_Bacteroidales_bacterium_55_9	intestinal_Linoleoyl ethanolamide	-0.305 (-0.5, -0.111)	0.004	0.004	-0.121 (-0.255, -0.025)	0.02	0.206	39.8
s_Coriobacteriaceae_bacterium	intestinal_2,3-Diphosphoglyceric acid	0.118 (0.015, 0.259)	0.012	0.049	0.046 (0.008, 0.103)	0.02	0.206	38.9
s_Coriobacteriaceae_bacterium	intestinal_Estrogen	0.118 (0.012, 0.259)	0.036	0.049	0.093 (0.03, 0.209)	0.008	0.192	79.2
s_Coriobacteriaceae_bacterium	intestinal_Linoleoyl ethanolamide	0.118 (0.008, 0.262)	0.024	0.049	0.059 (0.005, 0.148)	0.02	0.206	50.6
s_Alistipes_senegalensis	intestinal_2,3-Diphosphoglyceric acid	-0.538 (-0.968, -0.233)	<0.001	<0.001	-0.144 (-0.322, -0.029)	0.028	0.324	26.7
s_Alistipes_senegalensis	intestinal_Linoleoyl ethanolamide	-0.538 (-0.891, -0.216)	<0.001	<0.001	-0.228 (-0.557, -0.06)	0.004	0.288	42.4
s_Alistipes_senegalensis	intestinal_N2-gamma-Glutamylglutamine	-0.538 (-0.948, -0.175)	<0.001	<0.001	-0.176 (-0.356, -0.029)	0.032	0.324	32.7
s_Alistipes_finegoldii	intestinal_2,3-Diphosphoglyceric acid	-0.481 (-0.832, -0.181)	<0.001	<0.001	-0.131 (-0.292, -0.022)	0.032	0.432	27.1

s_Alistipes_finegoldii	intestinal_Linoleoyl ethanolamide	-0.481 (-0.812, -0.163)	<0.001	<0.001	-0.213 (-0.499, -0.049)	0.004	0.288	44.2
s_Alistipes_finegoldii	intestinal_N2-gamma-Glutamylglutamine	-0.481 (-0.88, -0.139)	0.008	0.008	-0.16 (-0.339, -0.013)	0.036	0.432	33.3
g_Odoribacter	intestinal_2,3-Diphosphoglyceric acid	-0.64 (-1.096, -0.174)	0.004	0.008	-0.192 (-0.479, -0.035)	0.02	0.720	30
g_Odoribacter	intestinal_Estrogen	-0.64 (-1.194, -0.161)	0.004	0.008	-0.243 (-0.624, -0.011)	0.04	0.720	38
s_Alistipes_sp_58_9_plus	intestinal_2,3-Diphosphoglyceric acid	-0.289 (-0.462, -0.116)	0.004	0.004	-0.071 (-0.137, -0.002)	0.048	0.346	24.4
s_Alistipes_sp_	intestinal_2,3-Diphosphoglyceric acid	-0.331 (-0.586, -0.085)	<0.001	<0.001	-0.101 (-0.212, -0.029)	0.012	0.288	30.6
s_Alistipes_sp_	intestinal_Linoleoyl ethanolamide	-0.331 (-0.595, -0.101)	0.008	0.008	-0.148 (-0.317, -0.005)	0.044	0.453	44.8
s_Alistipes_sp_	intestinal_N2-gamma-Glutamylglutamine	-0.331 (-0.562, -0.084)	0.004	0.005	-0.113 (-0.236, -0.017)	0.032	0.384	34
s_Bacteroides_caecimuris	intestinal_2,3-Diphosphoglyceric acid	-0.594 (-1.092, -0.151)	0.004	0.013	-0.184 (-0.392, -0.006)	0.044	0.617	31
s_Alistipes_onderdonkii	intestinal_2,3-Diphosphoglyceric acid	-0.469 (-0.82, -0.178)	<0.001	<0.001	-0.125 (-0.302, -0.016)	0.024	0.346	26.6
s_Alistipes_onderdonkii	intestinal_Linoleoyl ethanolamide	-0.469 (-0.803, -0.163)	0.004	0.004	-0.204 (-0.455, -0.027)	0.016	0.346	43.4
s_Alistipes_onderdonkii	intestinal_N2-gamma-Glutamylglutamine	-0.469 (-0.78, -0.189)	<0.001	<0.001	-0.153 (-0.321, -0.015)	0.036	0.360	32.6
s_Alistipes_shahii	intestinal_2,3-Diphosphoglyceric acid	-0.618 (-1.028, -0.271)	<0.001	<0.001	-0.163 (-0.343, -0.028)	0.032	0.288	26.3
s_Alistipes_shahii	intestinal_Linoleoyl ethanolamide	-0.618 (-1.01, -0.238)	<0.001	<0.001	-0.263 (-0.566, -0.049)	0.024	0.288	42.6
s_Alistipes_shahii	intestinal_N2-gamma-Glutamylglutamine	-0.618 (-0.995, -0.22)	<0.001	<0.001	-0.2 (-0.444, -0.01)	0.044	0.352	32.3
s_Alistipes_timonensis	intestinal_2,3-Diphosphoglyceric acid	-0.501 (-0.857, -0.198)	<0.001	<0.001	-0.135 (-0.313, -0.019)	0.02	0.288	27

	s_Alistipes_timonensis	intestinal_Linoleoyl ethanolamide	-0.501 (-0.842, -0.212)	<0.001	<0.001	-0.217 (-0.522, -0.031)	0.02	0.288	43.4
	s_Alistipes_timonensis	intestinal_N2-gamma-Glutamylglutamine	-0.501 (-0.822, -0.175)	<0.001	<0.001	-0.166 (-0.368, -0.038)	0.02	0.288	33.2
	s_Alistipes_sp_An66	intestinal_2,3-Diphosphoglyceric acid	-0.448 (-0.78, -0.181)	<0.001	<0.001	-0.118 (-0.27, -0.018)	0.012	0.288	26.4
	s_Alistipes_sp_An66	intestinal_Linoleoyl ethanolamide	-0.448 (-0.773, -0.141)	<0.001	<0.001	-0.188 (-0.397, -0.022)	0.036	0.324	41.8
	s_Alistipes_sp_An66	intestinal_N2-gamma-Glutamylglutamine	-0.448 (-0.789, -0.198)	<0.001	<0.001	-0.145 (-0.336, -0.012)	0.032	0.324	32.3
	s_Duncaniella_muris	intestinal_Linoleoyl ethanolamide	1.948 (0.551, 2.961)	0.016	0.018	0.884 (0.038, 2.071)	0.032	0.942	45.4
brain_Cystine	f_Erysipelotrichaceae	intestinal_Cer(d18:1/16:0)	-0.235 (-0.377, -0.128)	<0.001	<0.001	-0.15 (-0.339, -0.062)	0.004	0.144	64
	f_Erysipelotrichaceae	intestinal_PC(16:0/18:1(9Z))	-0.235 (-0.358, -0.126)	<0.001	<0.001	-0.092 (-0.286, -0.007)	0.032	0.220	39.3
	f_Erysipelotrichaceae	intestinal_Ubiquinone-2	-0.235 (-0.354, -0.138)	<0.001	<0.001	-0.151 (-0.315, -0.071)	<0.001	<0.001	64.5
	f_Erysipelotrichaceae	serum_Palmitoylethanolamide	-0.235 (-0.365, -0.13)	<0.001	<0.001	-0.111 (-0.286, 0)	0.048	0.220	47.2
	f_Erysipelotrichaceae	serum_Anandamide	-0.235 (-0.374, -0.12)	<0.001	<0.001	-0.102 (-0.272, -0.008)	0.04	0.220	43.3
	f_Erysipelotrichaceae	serum_Retinal	-0.235 (-0.354, -0.123)	<0.001	<0.001	-0.095 (-0.255, -0.003)	0.044	0.220	40.3
	f_Erysipelotrichaceae	serum_PS(18:1(11Z)/20:0)	-0.235 (-0.358, -0.133)	<0.001	<0.001	-0.123 (-0.256, -0.046)	0.012	0.144	52.3
	f_Erysipelotrichaceae	serum_LysoPC(17:0/0:0)	-0.235 (-0.351, -0.128)	<0.001	<0.001	-0.073 (-0.194, -0.001)	0.048	0.220	30.9
	f_Erysipelotrichaceae	serum_7-Ketodeoxycholic acid	-0.235 (-0.364, -0.119)	<0.001	<0.001	-0.084 (-0.274, -0.006)	0.036	0.220	35.8
	f_Erysipelotrichaceae	serum_Leukotriene C4	-0.235 (-0.363, -0.122)	0.004	0.004	-0.081 (-0.204, -0.018)	0.016	0.165	34.5
	c_Erysipelotrichia	intestinal_Cer(d18:1/16:0)	-0.242 (-0.377, -0.104)	0.008	0.008	-0.168 (-0.392, -0.063)	<0.001	<0.001	69.5
	c_Erysipelotrichia	intestinal_PC(16:0/18:1(9Z))	-0.242 (-0.391, -0.124)	<0.001	<0.001	-0.107 (-0.324, -0.007)	0.024	0.173	44.3
	c_Erysipelotrichia	intestinal_Ubiquinone-2	-0.242 (-0.386, -0.109)	<0.001	<0.001	-0.164 (-0.318, -0.074)	<0.001	<0.001	67.8
	c_Erysipelotrichia	serum_(±)-Tryptophan	-0.242 (-0.382, -0.118)	0.004	0.004	-0.117 (-0.303, -0.005)	0.048	0.204	48.6
	c_Erysipelotrichia	serum_Esterase	-0.242 (-0.36, -0.105)	<0.001	<0.001	-0.092 (-0.209, -0.001)	0.048	0.204	38.1

c_Erysipelotrichia	serum_Palmitoylethanolamide	-0.242 (-0.372, -0.124)	<0.001	<0.001	-0.128 (-0.311, -0.005)	0.036	0.204	53.2
c_Erysipelotrichia	serum_Sphinganine	-0.242 (-0.371, -0.114)	0.004	0.004	-0.094 (-0.246, -0.02)	0.016	0.144	38.8
c_Erysipelotrichia	serum_Glucose 6-phosphate	-0.242 (-0.361, -0.125)	<0.001	<0.001	-0.116 (-0.277, -0.008)	0.036	0.204	47.9
c_Erysipelotrichia	serum_PS(18:1(11Z)/20:0)	-0.242 (-0.363, -0.113)	<0.001	<0.001	-0.134 (-0.245, -0.045)	<0.001	<0.001	55.3
c_Erysipelotrichia	serum_Leukotriene C4	-0.242 (-0.368, -0.119)	<0.001	<0.001	-0.093 (-0.261, -0.02)	0.008	0.115	38.4
o_Erysipelotrichales	intestinal_Cer(d18:1/16:0)	-0.242 (-0.387, -0.118)	<0.001	<0.001	-0.168 (-0.388, -0.065)	<0.001	<0.001	69.5
o_Erysipelotrichales	intestinal_PC(16:0/18:1(9Z))	-0.242 (-0.372, -0.088)	0.008	0.008	-0.107 (-0.345, -0.008)	0.024	0.180	44.3
o_Erysipelotrichales	intestinal_Ubiquinone-2	-0.242 (-0.365, -0.117)	<0.001	<0.001	-0.164 (-0.328, -0.074)	<0.001	<0.001	67.8
o_Erysipelotrichales	serum_Stearoylethanolamide	-0.242 (-0.357, -0.122)	<0.001	<0.001	-0.12 (-0.288, -0.007)	0.04	0.180	49.8
o_Erysipelotrichales	serum_Heptadecanoic acid	-0.242 (-0.374, -0.131)	0.004	0.004	-0.13 (-0.293, -0.004)	0.044	0.186	53.8
o_Erysipelotrichales	serum_Esterase	-0.242 (-0.39, -0.109)	0.012	0.012	-0.092 (-0.198, -0.002)	0.04	0.180	38.1
o_Erysipelotrichales	serum_Palmitoylethanolamide	-0.242 (-0.396, -0.102)	0.004	0.004	-0.128 (-0.333, -0.015)	0.024	0.180	53.2
o_Erysipelotrichales	serum_Sphinganine	-0.242 (-0.371, -0.113)	<0.001	<0.001	-0.094 (-0.229, -0.01)	0.036	0.180	38.8
o_Erysipelotrichales	serum_Anandamide	-0.242 (-0.366, -0.116)	<0.001	<0.001	-0.113 (-0.293, -0.01)	0.04	0.180	46.8
o_Erysipelotrichales	serum_Glucose 6-phosphate	-0.242 (-0.38, -0.125)	<0.001	<0.001	-0.116 (-0.288, -0.011)	0.032	0.180	47.9
o_Erysipelotrichales	serum_PS(18:1(11Z)/20:0)	-0.242 (-0.374, -0.12)	<0.001	<0.001	-0.134 (-0.287, -0.042)	0.016	0.165	55.3
o_Erysipelotrichales	serum_LysoPC(17:0/0:0)	-0.242 (-0.38, -0.127)	<0.001	<0.001	-0.079 (-0.209, -0.003)	0.04	0.180	32.8
o_Erysipelotrichales	serum_Leukotriene C4	-0.242 (-0.375, -0.128)	<0.001	<0.001	-0.093 (-0.266, -0.023)	0.008	0.115	38.4
s_Erysipelotrichaceae_bacterium	intestinal_Cer(d18:1/16:0)	-0.25 (-0.419, -0.127)	<0.001	<0.001	-0.187 (-0.458, -0.065)	0.004	0.144	74.5
s_Erysipelotrichaceae_bacterium	intestinal_PC(16:0/18:1(9Z))	-0.25 (-0.416, -0.113)	<0.001	<0.001	-0.112 (-0.341, -0.006)	0.032	0.256	44.6
s_Erysipelotrichaceae_bacterium	intestinal_Ubiquinone-2	-0.25 (-0.419, -0.133)	<0.001	<0.001	-0.164 (-0.312, -0.066)	<0.001	<0.001	65.7
s_Erysipelotrichaceae_bacterium	serum_Sphinganine	-0.25 (-0.403, -0.122)	<0.001	<0.001	-0.091 (-0.257, -0.012)	0.016	0.230	36.3

s_Erysipelotrichaceae_bacterium	serum_Anandamide	-0.25 (-0.414, -0.111)	<0.001	<0.001	-0.11 (-0.288, -0.007)	0.036	0.259	43.8
s_Erysipelotrichaceae_bacterium	serum_Retinal	-0.25 (-0.427, -0.131)	<0.001	<0.001	-0.098 (-0.24, -0.005)	0.028	0.252	39.2
s_Erysipelotrichaceae_bacterium	serum_PS(18:1(11Z)/20:0)	-0.25 (-0.428, -0.143)	<0.001	<0.001	-0.133 (-0.271, -0.034)	0.02	0.240	53.1
s_Erysipelotrichaceae_bacterium	serum_Leukotriene C4	-0.25 (-0.418, -0.127)	<0.001	<0.001	-0.099 (-0.237, -0.017)	0.016	0.230	39.5
s_Muribaculaceae_bacterium_Isolate_037_Harlan	intestinal_Cortisone	0.807 (0.408, 1.31)	<0.001	<0.001	0.078 (0.005, 0.546)	0.036	0.216	9.6
s_Muribaculaceae_bacterium_Isolate_037_Harlan	intestinal_Docosahexaenoic acid	0.807 (0.407, 1.192)	<0.001	<0.001	0.206 (0.035, 0.529)	0.028	0.216	25.5
s_Muribaculaceae_bacterium_Isolate_037_Harlan	intestinal_Docosanol	0.807 (0.396, 1.176)	<0.001	<0.001	0.145 (0.012, 0.326)	0.044	0.216	18
s_Muribaculaceae_bacterium_Isolate_037_Harlan	intestinal_Ubiquinone-2	0.807 (0.439, 1.226)	<0.001	<0.001	0.342 (0.107, 0.734)	<0.001	<0.001	42.4
s_Muribaculaceae_bacterium_Isolate_037_Harlan	serum_Phenyllactic acid	0.807 (0.419, 1.201)	<0.001	<0.001	0.239 (0.036, 0.466)	0.032	0.216	29.6
s_Muribaculaceae_bacterium_Isolate_037_Harlan	serum_Stearoylethanolamide	0.807 (0.427, 1.232)	<0.001	<0.001	0.257 (0.035, 0.57)	0.02	0.216	31.8
s_Muribaculaceae_bacterium_Isolate_037_Harlan	serum_Heptadecanoic acid	0.807 (0.359, 1.211)	<0.001	<0.001	0.27 (0.033, 0.596)	0.028	0.216	33.5
s_Muribaculaceae_bacterium_Isolate_037_Harlan	serum_Palmitoylethanolamide	0.807 (0.314, 1.15)	<0.001	<0.001	0.257 (0.038, 0.605)	0.028	0.216	31.8
s_Muribaculaceae_bacterium_Isolate_037_Harlan	serum_Glycocholic acid	0.807 (0.449, 1.23)	<0.001	<0.001	0.257 (0.014, 0.553)	0.04	0.216	31.9
s_Muribaculaceae_bacterium_Isolate_037_Harlan	serum_Docosapentaenoic acid (22n-3)	0.807 (0.395, 1.203)	<0.001	<0.001	0.228 (0.009, 0.592)	0.044	0.216	28.3
s_Muribaculaceae_bacterium_Isolate_037_Harlan	serum_Anandamide	0.807 (0.482, 1.187)	<0.001	<0.001	0.258 (0.022, 0.611)	0.032	0.216	32
s_Muribaculaceae_bacterium_Isolate_037_Harlan	serum_1,6-Digalloyl-beta-D-glucopyranose	0.807 (0.417, 1.23)	0.004	0.004	0.203 (0.016, 0.445)	0.028	0.216	25.1

s_Muribaculaceae_bacterium_Isolate_037_Harlan	serum_Retinal	0.807 (0.381, 1.182)	<0.001	<0.001	0.281 (0.024, 0.639)	0.024	0.216	34.8
s_Muribaculaceae_bacterium_Isolate_037_Harlan	serum_PS(18:1(11Z)/20:0)	0.807 (0.357, 1.212)	<0.001	<0.001	0.311 (0.004, 0.592)	0.048	0.216	38.6
f_Rikenellaceae	intestinal_Cer(d18:1/16:0)	0.31 (0.175, 0.482)	<0.001	<0.001	0.169 (0.059, 0.476)	<0.001	<0.001	54.5
f_Rikenellaceae	intestinal_PC(16:0/18:1(9Z))	0.31 (0.194, 0.475)	<0.001	<0.001	0.101 (0.005, 0.285)	0.04	0.320	32.7
f_Rikenellaceae	intestinal_Ubiquinone-2	0.31 (0.182, 0.459)	<0.001	<0.001	0.246 (0.109, 0.531)	<0.001	<0.001	79.2
f_Rikenellaceae	serum_Dimyristoylphosphatidylcholine, DL	0.31 (0.179, 0.472)	<0.001	<0.001	0.066 (0.009, 0.252)	0.032	0.288	21.4
f_Rikenellaceae	serum_Glucose 6-phosphate	0.31 (0.178, 0.47)	<0.001	<0.001	0.097 (0.021, 0.28)	0.02	0.288	31.2
f_Rikenellaceae	serum_PS(18:1(11Z)/20:0)	0.31 (0.185, 0.48)	<0.001	<0.001	0.188 (0.043, 0.477)	0.028	0.288	60.5
f_Rikenellaceae	serum_7-Ketodeoxycholic acid	0.31 (0.18, 0.476)	<0.001	<0.001	0.094 (0.015, 0.279)	0.02	0.288	30.4
f_Rikenellaceae	serum_3a,6b,7b,12a-Tetrahydroxy-5b-cholanoic acid	0.31 (0.192, 0.494)	<0.001	<0.001	0.087 (0.014, 0.266)	0.012	0.288	28.1
f_Rikenellaceae	serum_Leukotriene C4	0.31 (0.185, 0.47)	<0.001	<0.001	0.094 (0.018, 0.237)	0.024	0.288	30.3
g_Alistipes	intestinal_Cer(d18:1/16:0)	0.274 (0.174, 0.424)	<0.001	<0.001	0.148 (0.063, 0.411)	<0.001	<0.001	54.2
g_Alistipes	intestinal_Ubiquinone-2	0.274 (0.164, 0.425)	<0.001	<0.001	0.207 (0.08, 0.46)	0.004	0.144	75.6
g_Alistipes	serum_Dimyristoylphosphatidylcholine, DL	0.274 (0.163, 0.407)	<0.001	<0.001	0.056 (0.003, 0.212)	0.036	0.324	20.5
g_Alistipes	serum_Glucose 6-phosphate	0.274 (0.164, 0.425)	<0.001	<0.001	0.081 (0.006, 0.201)	0.036	0.324	29.7
g_Alistipes	serum_PS(18:1(11Z)/20:0)	0.274 (0.162, 0.417)	<0.001	<0.001	0.158 (0.022, 0.373)	0.028	0.324	57.7
g_Alistipes	serum_7-Ketodeoxycholic acid	0.274 (0.165, 0.408)	<0.001	<0.001	0.08 (0.01, 0.24)	0.016	0.324	29.3
g_Alistipes	serum_3a,6b,7b,12a-Tetrahydroxy-5b-cholanoic acid	0.274 (0.162, 0.414)	<0.001	<0.001	0.074 (0.009, 0.23)	0.024	0.324	26.9
g_Alistipes	serum_Leukotriene C4	0.274 (0.161, 0.419)	<0.001	<0.001	0.079 (0.004, 0.182)	0.032	0.324	28.7

s_Prevotella_sp_PMUR	intestinal_Cer(d18:1/16:0)	0.227 (0.092, 0.366)	0.004	0.010	0.153 (0.05, 0.276)	<0.001	<0.001	67.4
s_Prevotella_sp_PMUR	intestinal_Docosahexaenoic acid	0.227 (0.07, 0.369)	0.008	0.013	0.108 (0.012, 0.349)	0.036	0.118	47.5
s_Prevotella_sp_PMUR	intestinal_Ubiquinone-2	0.227 (0.081, 0.362)	0.008	0.013	0.115 (0.037, 0.286)	0.016	0.068	50.6
s_Prevotella_sp_PMUR	serum_(±)-Tryptophan	0.227 (0.078, 0.364)	0.012	0.015	0.134 (0.029, 0.301)	0.012	0.068	59
s_Prevotella_sp_PMUR	serum_Arachidoyl Ethanolamide	0.227 (0.091, 0.367)	0.004	0.010	0.108 (0.022, 0.252)	0.016	0.068	47.6
s_Prevotella_sp_PMUR	serum_Phenyllactic acid	0.227 (0.094, 0.37)	0.016	0.017	0.114 (0.036, 0.247)	0.008	0.068	50.1
s_Prevotella_sp_PMUR	serum_Stearoylethanolamide	0.227 (0.069, 0.361)	0.004	0.010	0.113 (0.033, 0.258)	0.012	0.068	49.7
s_Prevotella_sp_PMUR	serum_Heptadecanoic acid	0.227 (0.072, 0.353)	0.012	0.015	0.119 (0.035, 0.249)	<0.001	<0.001	52.3
s_Prevotella_sp_PMUR	serum_Esterase	0.227 (0.053, 0.367)	0.016	0.017	0.096 (0.021, 0.25)	0.012	0.068	42.4
s_Prevotella_sp_PMUR	serum_Palmitoylethanolamide	0.227 (0.078, 0.356)	0.008	0.013	0.113 (0.034, 0.253)	0.004	0.058	49.9
s_Prevotella_sp_PMUR	serum_Glycocholic acid	0.227 (0.068, 0.361)	0.012	0.015	0.113 (0.021, 0.229)	0.012	0.068	49.7
s_Prevotella_sp_PMUR	serum_Sphingosine	0.227 (0.067, 0.382)	0.008	0.013	0.135 (0.027, 0.263)	0.028	0.096	59.6
s_Prevotella_sp_PMUR	serum_PC(16:0/20:4(5Z,8Z,11Z,14Z))	0.227 (0.077, 0.364)	<0.001	<0.001	0.09 (0.009, 0.188)	0.024	0.091	39.5
s_Prevotella_sp_PMUR	serum_1-Palmitoylphosphatidylcholine	0.227 (0.065, 0.366)	0.004	0.010	0.094 (0.016, 0.241)	<0.001	<0.001	41.5
s_Prevotella_sp_PMUR	serum_Sphinganine	0.227 (0.086, 0.358)	0.004	0.010	0.075 (0.016, 0.207)	0.008	0.068	33.3
s_Prevotella_sp_PMUR	serum_Deoxycholic acid	0.227 (0.103, 0.364)	0.004	0.010	0.098 (0.017, 0.212)	0.024	0.091	43.1
s_Prevotella_sp_PMUR	serum_Docosapentaenoic acid (22n-3)	0.227 (0.059, 0.366)	0.004	0.010	0.099 (0.017, 0.212)	0.012	0.068	43.5
s_Prevotella_sp_PMUR	serum_Anandamide	0.227 (0.083, 0.358)	0.008	0.013	0.107 (0.031, 0.235)	<0.001	<0.001	47.1
s_Prevotella_sp_PMUR	serum_1-Methylguanosine	0.227 (0.074, 0.36)	0.008	0.013	0.097 (0.012, 0.2)	0.016	0.068	43
s_Prevotella_sp_PMUR	serum_Retinal	0.227 (0.077, 0.349)	0.004	0.010	0.098 (0.018, 0.241)	0.016	0.068	43.4
s_Prevotella_sp_PMUR	serum_PS(18:1(11Z)/20:0)	0.227 (0.076, 0.358)	<0.001	<0.001	0.111 (0.014, 0.262)	0.028	0.096	49.1
s_Prevotella_sp_PMUR	serum_LysoPC(17:0/0:0)	0.227 (0.063, 0.362)	0.016	0.017	0.073 (0.006, 0.203)	0.04	0.122	32.2
g_Duncaniella	intestinal_Cortisone	-0.363 (-0.703, -0.083)	0.016	0.029	-0.111 (-0.45, -0.02)	0.032	0.110	30.6

g_Duncaniella	intestinal_Docosahexaenoic acid	-0.363 (-0.743, -0.049)	0.016	0.029	-0.31 (-0.596, -0.053)	0.02	0.085	85.4
g_Duncaniella	intestinal_Docosanol	-0.363 (-0.736, -0.099)	0.016	0.029	-0.265 (-0.589, -0.019)	0.04	0.120	72.8
g_Duncaniella	serum_(±)-Tryptophan	-0.363 (-0.776, -0.072)	0.024	0.029	-0.271 (-0.575, -0.083)	0.02	0.085	74.5
g_Duncaniella	serum_Arachidoyl Ethanolamide	-0.363 (-0.751, -0.098)	0.016	0.029	-0.312 (-0.639, -0.091)	0.016	0.082	85.8
g_Duncaniella	serum_Phenyllactic acid	-0.363 (-0.703, -0.065)	0.024	0.029	-0.352 (-0.726, -0.119)	0.016	0.082	96.9
g_Duncaniella	serum_Stearoylethanolamide	-0.363 (-0.763, -0.088)	0.020	0.029	-0.321 (-0.647, -0.119)	0.004	0.058	88.4
g_Duncaniella	serum_Esterase	-0.363 (-0.696, -0.026)	0.020	0.029	-0.246 (-0.674, -0.083)	0.008	0.058	67.8
g_Duncaniella	serum_Palmitoylethanolamide	-0.363 (-0.8, -0.06)	0.032	0.033	-0.311 (-0.625, -0.121)	0.02	0.085	85.7
g_Duncaniella	serum_Glycocholic acid	-0.363 (-0.724, -0.054)	0.028	0.031	-0.31 (-0.609, -0.089)	0.008	0.058	85.4
g_Duncaniella	serum_Palmitic acid	-0.363 (-0.737, -0.069)	0.012	0.029	-0.239 (-0.541, -0.025)	0.04	0.120	65.7
g_Duncaniella	serum_PC(16:0/20:4(5Z,8Z,11Z,14Z))	-0.363 (-0.738, -0.058)	0.020	0.029	-0.258 (-0.6, -0.093)	0.008	0.058	71.1
g_Duncaniella	serum_PC(16:0/18:1(9Z))	-0.363 (-0.865, -0.093)	0.008	0.029	-0.177 (-0.589, -0.01)	0.036	0.118	48.6
g_Duncaniella	serum_1-Palmitoylphosphatidylcholine	-0.363 (-0.834, -0.036)	0.032	0.033	-0.284 (-0.614, -0.116)	0.004	0.058	78.2
g_Duncaniella	serum_Sphinganine	-0.363 (-0.738, -0.084)	0.004	0.029	-0.264 (-0.511, -0.081)	0.028	0.101	72.6
g_Duncaniella	serum_Docosapentaenoic acid (22n-3)	-0.363 (-0.679, -0.055)	0.020	0.029	-0.296 (-0.586, -0.093)	0.012	0.079	81.4
g_Duncaniella	serum_Anandamide	-0.363 (-0.801, -0.037)	0.036	0.036	-0.338 (-0.68, -0.119)	0.008	0.058	92.9
g_Duncaniella	serum_Alkergot	-0.363 (-0.787, -0.062)	0.020	0.029	-0.25 (-0.58, -0.014)	0.044	0.127	68.7
g_Duncaniella	serum_1,6-Digalloyl-beta-D-glucopyranose	-0.363 (-0.702, -0.069)	0.020	0.029	-0.242 (-0.477, -0.051)	0.016	0.082	66.6
g_Duncaniella	serum_1-Methylguanosine	-0.363 (-0.738, -0.091)	0.004	0.029	-0.267 (-0.629, -0.047)	0.024	0.096	73.5
g_Duncaniella	serum_Retinal	-0.363 (-0.779, -0.089)	0.012	0.029	-0.338 (-0.65, -0.115)	<0.001	<0.001	92.9
g_Duncaniella	serum_LysoPC(17:0/0:0)	-0.363 (-0.741, -0.048)	0.032	0.033	-0.218 (-0.502, -0.051)	0.028	0.101	60
g_Heminiphilus	intestinal_Docosahexaenoic acid	0.232 (0.038, 0.401)	0.020	0.050	0.128 (0.023, 0.351)	0.016	0.080	55.2

g_Heminiphilus	intestinal_Docosanol	0.232 (0.024, 0.403)	0.032	0.050	0.102 (0.025, 0.219)	0.016	0.080	44
g_Heminiphilus	intestinal_gamma-Linolenic acid	0.232 (0.025, 0.402)	0.044	0.050	0.092 (0.008, 0.24)	0.032	0.096	39.6
g_Heminiphilus	intestinal_Ubiquinone-2	0.232 (0.024, 0.39)	0.040	0.050	0.17 (0.02, 0.346)	0.024	0.082	73.1
g_Heminiphilus	serum_(±)-Tryptophan	0.232 (0.014, 0.402)	0.032	0.050	0.173 (0.042, 0.339)	0.012	0.080	74.6
g_Heminiphilus	serum_Arachidoyl Ethanolamide	0.232 (0.005, 0.404)	0.044	0.050	0.133 (0.048, 0.259)	0.008	0.072	57.4
g_Heminiphilus	serum_Phenyllactic acid	0.232 (0.013, 0.401)	0.044	0.050	0.151 (0.065, 0.236)	0.008	0.072	65.2
g_Heminiphilus	serum_Stearoylethanolamide	0.232 (0.054, 0.395)	0.020	0.050	0.135 (0.035, 0.277)	0.02	0.080	58.2
g_Heminiphilus	serum_Heptadecanoic acid	0.232 (0.016, 0.39)	0.048	0.050	0.145 (0.057, 0.255)	0.012	0.080	62.4
g_Heminiphilus	serum_Esterase	0.232 (0.03, 0.405)	0.024	0.050	0.102 (0.04, 0.215)	0.004	0.072	44.1
g_Heminiphilus	serum_Palmitoylethanolamide	0.232 (0.016, 0.397)	0.036	0.050	0.13 (0.042, 0.23)	0.008	0.072	56.1
g_Heminiphilus	serum_Glycocholic acid	0.232 (0.01, 0.394)	0.040	0.050	0.119 (0.029, 0.236)	0.016	0.080	51.5
g_Heminiphilus	serum_Palmitic acid	0.232 (0.044, 0.403)	0.016	0.050	0.093 (0.006, 0.21)	0.032	0.096	40.1
g_Heminiphilus	serum_PC(16:0/20:4(5Z,8Z,11Z,14Z))	0.232 (0.058, 0.406)	0.028	0.050	0.098 (0.011, 0.192)	0.028	0.092	42.1
g_Heminiphilus	serum_1-Palmitoylphosphatidylcholine	0.232 (0.026, 0.392)	0.020	0.050	0.109 (0.035, 0.229)	0.008	0.072	47.1
g_Heminiphilus	serum_Sphinganine	0.232 (0.012, 0.407)	0.044	0.050	0.104 (0.013, 0.254)	0.02	0.080	44.7
g_Heminiphilus	serum_Deoxycholic acid	0.232 (0.014, 0.401)	0.028	0.050	0.103 (0.012, 0.227)	0.02	0.080	44.4
g_Heminiphilus	serum_Docosapentaenoic acid (22n-3)	0.232 (0.038, 0.394)	0.024	0.050	0.118 (0.034, 0.229)	0.008	0.072	51
g_Heminiphilus	serum_Alkergot	0.232 (0.031, 0.414)	0.032	0.050	0.098 (0.003, 0.23)	0.044	0.117	42.1
g_Heminiphilus	serum_1,6-Digalloyl-beta-D-glucopyranose	0.232 (0.007, 0.4)	0.048	0.050	0.089 (0.01, 0.19)	0.036	0.104	38.4
g_Heminiphilus	serum_1-Methylguanosine	0.232 (0.016, 0.393)	0.044	0.050	0.11 (0.002, 0.234)	0.04	0.111	47.5
g_Heminiphilus	serum_Retinal	0.232 (0.032, 0.409)	0.032	0.050	0.109 (0.013, 0.244)	0.016	0.080	47
g_Heminiphilus	serum_Phytosphingosine-1-P	0.232 (0.041, 0.405)	0.012	0.050	0.091 (0.007, 0.267)	0.024	0.082	39.3

s_Heminiphilus_faecis	intestinal_Cortisone	0.232 (0.02, 0.406)	0.032	0.052	0.057 (0.001, 0.232)	0.048	0.144	24.5
s_Heminiphilus_faecis	intestinal_Docosahexaenoic acid	0.232 (0.026, 0.39)	0.040	0.052	0.128 (0.022, 0.348)	0.012	0.072	55.2
s_Heminiphilus_faecis	intestinal_Docosanol	0.232 (0.035, 0.388)	0.032	0.052	0.102 (0.016, 0.229)	0.024	0.096	44
s_Heminiphilus_faecis	intestinal_gamma-Linolenic acid	0.232 (0.03, 0.414)	0.020	0.052	0.092 (0.018, 0.242)	0.012	0.072	39.6
s_Heminiphilus_faecis	intestinal_Ubiquinone-2	0.232 (0.039, 0.415)	0.024	0.052	0.17 (0.043, 0.36)	0.016	0.077	73.1
s_Heminiphilus_faecis	serum_(±)-Tryptophan	0.232 (0.029, 0.413)	0.032	0.052	0.173 (0.054, 0.319)	0.016	0.077	74.6
s_Heminiphilus_faecis	serum_Arachidoyl Ethanolamide	0.232 (0.009, 0.385)	0.048	0.053	0.133 (0.041, 0.238)	0.008	0.072	57.4
s_Heminiphilus_faecis	serum_Phenyllactic acid	0.232 (0.025, 0.394)	0.028	0.052	0.151 (0.072, 0.257)	<0.001	<0.001	65.2
s_Heminiphilus_faecis	serum_Stearoylethanolamide	0.232 (0.002, 0.415)	0.048	0.053	0.135 (0.034, 0.258)	0.032	0.105	58.2
s_Heminiphilus_faecis	serum_Heptadecanoic acid	0.232 (0.022, 0.403)	0.032	0.052	0.145 (0.053, 0.274)	0.008	0.072	62.4
s_Heminiphilus_faecis	serum_Esterase	0.232 (0.049, 0.384)	0.012	0.052	0.102 (0.032, 0.213)	0.012	0.072	44.1
s_Heminiphilus_faecis	serum_Palmitoylethanolamide	0.232 (0.032, 0.402)	0.028	0.052	0.13 (0.046, 0.235)	0.012	0.072	56.1
s_Heminiphilus_faecis	serum_Glycocholic acid	0.232 (0.028, 0.398)	0.032	0.052	0.119 (0.025, 0.252)	0.004	0.072	51.5
s_Heminiphilus_faecis	serum_1-Palmitoylphosphatidylcholine	0.232 (0.034, 0.414)	0.008	0.052	0.109 (0.029, 0.205)	0.016	0.077	47.1
s_Heminiphilus_faecis	serum_Deoxycholic acid	0.232 (0.026, 0.382)	0.024	0.052	0.103 (0.012, 0.233)	0.024	0.096	44.4
s_Heminiphilus_faecis	serum_Docosapentaenoic acid (22n-3)	0.232 (0.038, 0.389)	0.020	0.052	0.118 (0.027, 0.239)	0.012	0.072	51
s_Heminiphilus_faecis	serum_Anandamide	0.232 (0.03, 0.393)	0.032	0.052	0.127 (0.029, 0.255)	0.008	0.072	54.9
s_Heminiphilus_faecis	serum_1,6-Digalloyl-beta-D-glucopyranose	0.232 (0.034, 0.395)	0.024	0.052	0.089 (0.001, 0.195)	0.048	0.144	38.4
s_Heminiphilus_faecis	serum_1-Methylguanosine	0.232 (0.002, 0.395)	0.040	0.052	0.11 (0.016, 0.23)	0.032	0.105	47.5
s_Duncaniella_dubosii	intestinal_Docosahexaenoic acid	-0.252 (-0.514, -0.048)	0.016	0.025	-0.198 (-0.402, -0.037)	0.028	0.088	78.5
s_Duncaniella_dubosii	intestinal_gamma-Linolenic acid	-0.252 (-0.506, -0.048)	0.004	0.023	-0.114 (-0.311, -0.011)	0.028	0.088	45.1

s_Duncaniella_dubosii	intestinal_PC(16:0/18:1(9Z))	-0.252 (-0.519, -0.034)	0.020	0.025	-0.115 (-0.276, -0.001)	0.048	0.125	45.4
s_Duncaniella_dubosii	serum_(±)-Tryptophan	-0.252 (-0.479, -0.057)	0.012	0.023	-0.196 (-0.446, -0.063)	0.024	0.086	77.7
s_Duncaniella_dubosii	serum_Arachidoyl Ethanolamide	-0.252 (-0.498, -0.042)	0.012	0.023	-0.197 (-0.369, -0.062)	0.012	0.072	78
s_Duncaniella_dubosii	serum_Phenyllactic acid	-0.252 (-0.528, -0.058)	0.008	0.023	-0.225 (-0.423, -0.088)	<0.001	<0.001	89.2
s_Duncaniella_dubosii	serum_Stearoylethanolamide	-0.252 (-0.488, -0.059)	0.008	0.023	-0.199 (-0.422, -0.073)	0.008	0.064	78.8
s_Duncaniella_dubosii	serum_Heptadecanoic acid	-0.252 (-0.475, -0.06)	0.008	0.023	-0.223 (-0.4, -0.107)	0.004	0.064	88.6
s_Duncaniella_dubosii	serum_Esterase	-0.252 (-0.543, -0.028)	0.020	0.025	-0.156 (-0.394, -0.053)	0.008	0.064	61.9
s_Duncaniella_dubosii	serum_Palmitoylethanolamide	-0.252 (-0.525, -0.062)	0.020	0.025	-0.195 (-0.374, -0.066)	0.016	0.072	77.4
s_Duncaniella_dubosii	serum_Glycocholic acid	-0.252 (-0.531, -0.067)	0.004	0.023	-0.19 (-0.362, -0.059)	0.012	0.072	75.3
s_Duncaniella_dubosii	serum_Palmitic acid	-0.252 (-0.492, -0.028)	0.028	0.029	-0.15 (-0.317, -0.022)	0.036	0.108	59.5
s_Duncaniella_dubosii	serum_PC(16:0/20:4(5Z,8Z,11Z,14Z))	-0.252 (-0.508, -0.043)	0.012	0.023	-0.165 (-0.367, -0.045)	0.016	0.072	65.4
s_Duncaniella_dubosii	serum_PC(16:0/18:1(9Z))	-0.252 (-0.548, -0.03)	0.028	0.029	-0.109 (-0.325, -0.011)	0.028	0.088	43.3
s_Duncaniella_dubosii	serum_1-Palmitoylphosphatidylcholine	-0.252 (-0.505, -0.047)	0.008	0.023	-0.173 (-0.331, -0.055)	0.016	0.072	68.4
s_Duncaniella_dubosii	serum_Sphinganine	-0.252 (-0.473, -0.049)	0.016	0.025	-0.158 (-0.319, -0.042)	0.008	0.064	62.7
s_Duncaniella_dubosii	serum_Deoxycholic acid	-0.252 (-0.497, -0.071)	0.008	0.023	-0.165 (-0.362, -0.032)	0.02	0.076	65.3
s_Duncaniella_dubosii	serum_Docosapentaenoic acid (22n-3)	-0.252 (-0.512, -0.065)	0.008	0.023	-0.177 (-0.367, -0.037)	0.008	0.064	70.3
s_Duncaniella_dubosii	serum_Anandamide	-0.252 (-0.509, -0.046)	0.024	0.026	-0.204 (-0.385, -0.061)	0.016	0.072	80.8
s_Duncaniella_dubosii	serum_1,6-Digalloyl-beta-D-glucopyranose	-0.252 (-0.591, -0.046)	0.024	0.026	-0.156 (-0.307, -0.032)	0.02	0.076	61.7
s_Duncaniella_dubosii	serum_1-Methylguanosine	-0.252 (-0.489, -0.052)	0.012	0.023	-0.165 (-0.336, -0.024)	0.02	0.076	65.3
s_Duncaniella_dubosii	serum_Retinal	-0.252 (-0.504, -0.051)	0.012	0.023	-0.189 (-0.373, -0.049)	0.008	0.064	74.8
s_Duncaniella_dubosii	serum_Phytosphingosine-1-P	-0.252 (-0.516, -0.065)	0.012	0.023	-0.177 (-0.396, -0.008)	0.044	0.125	70.3
s_Duncaniella_dubosii	serum_LysoPC(17:0/0:0)	-0.252 (-0.533, -0.057)	0.016	0.025	-0.127 (-0.32, -0.027)	0.012	0.072	50.2

g_Faecalibaculum	intestinal_Cer(d18:1/16:0)	-0.15 (-0.214, -0.086)	<0.001	<0.001	-0.086 (-0.209, -0.032)	<0.001	<0.001	57.2
g_Faecalibaculum	intestinal_PC(16:0/18:1(9Z))	-0.15 (-0.221, -0.094)	<0.001	<0.001	-0.052 (-0.184, -0.002)	0.032	0.160	34.5
g_Faecalibaculum	intestinal_Ubiquinone-2	-0.15 (-0.223, -0.092)	<0.001	<0.001	-0.092 (-0.193, -0.044)	<0.001	<0.001	61
g_Faecalibaculum	serum_Stearoylethanolamide	-0.15 (-0.22, -0.087)	<0.001	<0.001	-0.055 (-0.159, -0.006)	0.028	0.160	36.6
g_Faecalibaculum	serum_Heptadecanoic acid	-0.15 (-0.225, -0.085)	<0.001	<0.001	-0.06 (-0.158, -0.004)	0.028	0.160	39.6
g_Faecalibaculum	serum_Palmitoylethanolamide	-0.15 (-0.232, -0.091)	<0.001	<0.001	-0.058 (-0.145, -0.002)	0.048	0.173	38.2
g_Faecalibaculum	serum_1-Palmitoylphosphatidylcholine	-0.15 (-0.221, -0.084)	<0.001	<0.001	-0.047 (-0.127, -0.002)	0.044	0.167	31.4
g_Faecalibaculum	serum_Sphinganine	-0.15 (-0.215, -0.086)	<0.001	<0.001	-0.046 (-0.116, -0.006)	0.04	0.160	30.9
g_Faecalibaculum	serum_Anandamide	-0.15 (-0.224, -0.091)	<0.001	<0.001	-0.055 (-0.154, -0.009)	0.024	0.160	36.7
g_Faecalibaculum	serum_Retinal	-0.15 (-0.224, -0.086)	<0.001	<0.001	-0.054 (-0.144, -0.001)	0.04	0.160	35.8
g_Faecalibaculum	serum_PS(18:1(11Z)/20:0)	-0.15 (-0.223, -0.092)	<0.001	<0.001	-0.072 (-0.152, -0.021)	0.012	0.108	47.9
g_Faecalibaculum	serum_LysoPC(17:0/0:0)	-0.15 (-0.215, -0.085)	<0.001	<0.001	-0.04 (-0.106, -0.005)	0.02	0.160	26.6
g_Faecalibaculum	serum_3a,6b,7b,12a-Tetrahydroxy-5b-cholanoic acid	-0.15 (-0.216, -0.089)	<0.001	<0.001	-0.044 (-0.127, -0.003)	0.036	0.160	29.1
g_Faecalibaculum	serum_Leukotriene C4	-0.15 (-0.22, -0.096)	<0.001	<0.001	-0.046 (-0.122, -0.011)	<0.001	<0.001	30.2
g_Faecalibaculum	serum_gamma-Linolenic acid	-0.15 (-0.221, -0.094)	<0.001	<0.001	-0.027 (-0.091, 0)	0.04	0.160	17.8
s_Faecalibaculum_rodentium	intestinal_Cer(d18:1/16:0)	-0.15 (-0.212, -0.087)	<0.001	<0.001	-0.086 (-0.221, -0.032)	0.004	0.058	57.2
s_Faecalibaculum_rodentium	intestinal_PC(16:0/18:1(9Z))	-0.15 (-0.21, -0.087)	<0.001	<0.001	-0.052 (-0.166, -0.008)	0.016	0.120	34.5
s_Faecalibaculum_rodentium	intestinal_Ubiquinone-2	-0.15 (-0.226, -0.088)	<0.001	<0.001	-0.092 (-0.183, -0.042)	<0.001	<0.001	61
s_Faecalibaculum_rodentium	serum_Stearoylethanolamide	-0.15 (-0.214, -0.091)	<0.001	<0.001	-0.055 (-0.142, -0.005)	0.04	0.180	36.6

s_Faecalibaculum_rodentium	serum_Sphinganine	-0.15 (-0.216, -0.09)	<0.001	<0.001	-0.046 (-0.133, -0.007)	0.02	0.120	30.9
s_Faecalibaculum_rodentium	serum_Docosapentaenoic acid (22n-3)	-0.15 (-0.22, -0.088)	<0.001	<0.001	-0.048 (-0.139, 0)	0.048	0.183	31.8
s_Faecalibaculum_rodentium	serum_Anandamide	-0.15 (-0.22, -0.09)	<0.001	<0.001	-0.055 (-0.145, -0.007)	0.02	0.120	36.7
s_Faecalibaculum_rodentium	serum_Retinal	-0.15 (-0.217, -0.084)	<0.001	<0.001	-0.054 (-0.14, -0.004)	0.032	0.177	35.8
s_Faecalibaculum_rodentium	serum_PS(18:1(11Z)/20:0)	-0.15 (-0.219, -0.09)	<0.001	<0.001	-0.072 (-0.166, -0.018)	<0.001	<0.001	47.9
s_Faecalibaculum_rodentium	serum_7-Ketodeoxycholic acid	-0.15 (-0.218, -0.089)	<0.001	<0.001	-0.048 (-0.153, -0.002)	0.036	0.180	31.7
s_Faecalibaculum_rodentium	serum_Leukotriene C4	-0.15 (-0.227, -0.093)	<0.001	<0.001	-0.046 (-0.11, -0.011)	0.008	0.072	30.2
s_Faecalibaculum_rodentium	serum_gamma-Linolenic acid	-0.15 (-0.213, -0.085)	<0.001	<0.001	-0.027 (-0.097, 0)	0.044	0.183	17.8
g_Allobaculum	intestinal_Cer(d18:1/16:0)	-0.069 (-0.124, -0.029)	<0.001	<0.001	-0.034 (-0.115, -0.004)	0.016	0.132	50.1
g_Allobaculum	intestinal_Docosahexaenoic acid	-0.069 (-0.121, -0.025)	<0.001	<0.001	-0.037 (-0.103, -0.004)	0.028	0.132	53.9
g_Allobaculum	intestinal_gamma-Linolenic acid	-0.069 (-0.119, -0.026)	<0.001	<0.001	-0.025 (-0.056, -0.004)	0.024	0.132	37
g_Allobaculum	intestinal_Ubiquinone-2	-0.069 (-0.123, -0.023)	<0.001	<0.001	-0.054 (-0.117, -0.021)	<0.001	<0.001	78.2
g_Allobaculum	serum_Stearoylethanolamide	-0.069 (-0.125, -0.027)	0.004	0.004	-0.043 (-0.079, -0.008)	0.032	0.132	62.6
g_Allobaculum	serum_Heptadecanoic acid	-0.069 (-0.12, -0.031)	<0.001	<0.001	-0.046 (-0.086, -0.003)	0.04	0.132	66.7
g_Allobaculum	serum_Esterase	-0.069 (-0.121, -0.027)	0.004	0.004	-0.031 (-0.069, -0.004)	0.036	0.132	45.8
g_Allobaculum	serum_Palmitoylethanolamide	-0.069 (-0.119, -0.028)	0.004	0.004	-0.043 (-0.08, -0.01)	0.016	0.132	61.9
g_Allobaculum	serum_Glycocholic acid	-0.069 (-0.111, -0.029)	<0.001	<0.001	-0.041 (-0.074, -0.004)	0.048	0.133	60.1
g_Allobaculum	serum_PC(16:0/20:4(5Z,8Z,11Z,14Z))	-0.069 (-0.119, -0.026)	<0.001	<0.001	-0.032 (-0.069, -0.001)	0.044	0.132	46

g_Allobaculum	serum_1-Palmitoylphosphatidylcholine	-0.069 (-0.113, -0.03)	<0.001	<0.001	-0.036 (-0.071, -0.004)	0.024	0.132	51.9
g_Allobaculum	serum_Sphinganine	-0.069 (-0.124, -0.031)	<0.001	<0.001	-0.03 (-0.062, -0.004)	0.02	0.132	43.4
g_Allobaculum	serum_Dimyristoylphosphatidylcholine, DL	-0.069 (-0.116, -0.028)	<0.001	<0.001	-0.017 (-0.081, -0.001)	0.036	0.132	25.1
g_Allobaculum	serum_Docosapentaenoic acid (22n-3)	-0.069 (-0.112, -0.029)	0.004	0.004	-0.039 (-0.078, -0.001)	0.044	0.132	56.4
g_Allobaculum	serum_Anandamide	-0.069 (-0.115, -0.028)	<0.001	<0.001	-0.044 (-0.083, -0.011)	0.004	0.096	64.3
g_Allobaculum	serum_1,6-Digalloyl-beta-D-glucopyranose	-0.069 (-0.113, -0.028)	0.004	0.004	-0.031 (-0.059, -0.007)	0.028	0.132	44.9
g_Allobaculum	serum_Glucose 6-phosphate	-0.069 (-0.123, -0.034)	<0.001	<0.001	-0.024 (-0.058, -0.005)	0.004	0.096	35.3
g_Allobaculum	serum_Retinal	-0.069 (-0.128, -0.025)	0.008	0.008	-0.042 (-0.083, -0.004)	0.044	0.132	61.6
g_Allobaculum	serum_PS(18:1(11Z)/20:0)	-0.069 (-0.132, -0.029)	0.008	0.008	-0.039 (-0.093, -0.006)	0.012	0.132	57.2
g_Allobaculum	serum_LysoPC(17:0/0:0)	-0.069 (-0.115, -0.025)	0.004	0.004	-0.027 (-0.064, -0.003)	0.028	0.132	39.7
g_Allobaculum	serum_7-Ketodeoxycholic acid	-0.069 (-0.115, -0.023)	<0.001	<0.001	-0.023 (-0.069, -0.001)	0.048	0.133	33
g_Allobaculum	serum_3a,6b,7b,12a-Tetrahydroxy-5b-cholanoic acid	-0.069 (-0.115, -0.03)	<0.001	<0.001	-0.021 (-0.065, -0.001)	0.044	0.132	30.6
s_Allobaculum_sp_539	intestinal_Cer(d18:1/16:0)	-0.061 (-0.101, -0.022)	<0.001	<0.001	-0.031 (-0.081, -0.007)	0.008	0.144	51.1
s_Allobaculum_sp_539	intestinal_PC(16:0/18:1(9Z))	-0.061 (-0.106, -0.029)	<0.001	<0.001	-0.02 (-0.059, -0.002)	0.024	0.209	33.1
s_Allobaculum_sp_539	intestinal_Ubiquinone-2	-0.061 (-0.108, -0.027)	<0.001	<0.001	-0.046 (-0.097, -0.024)	<0.001	<0.001	75.1
s_Allobaculum_sp_539	serum_Heptadecanoic acid	-0.061 (-0.103, -0.031)	<0.001	<0.001	-0.036 (-0.07, -0.001)	0.04	0.209	59.1
s_Allobaculum_sp_539	serum_Esterase	-0.061 (-0.104, -0.03)	0.004	0.004	-0.024 (-0.049, -0.005)	0.016	0.209	39.8
s_Allobaculum_sp_539	serum_Dimyristoylphosphatidylcholine, DL	-0.061 (-0.106, -0.025)	<0.001	<0.001	-0.015 (-0.069, -0.001)	0.04	0.209	25.3
s_Allobaculum_sp_539	serum_Glucose 6-phosphate	-0.061 (-0.1, -0.027)	<0.001	<0.001	-0.021 (-0.046, -0.006)	<0.001	<0.001	34.9
s_Allobaculum_sp_539	serum_Retinal	-0.061 (-0.101, -0.024)	<0.001	<0.001	-0.035 (-0.076, -0.004)	0.024	0.209	57.8

s_Allobaculum_sp_539	serum_PS(18:1(11Z)/20:0)	-0.061 (-0.103, -0.032)	<0.001	<0.001	-0.035 (-0.082, -0.01)	0.004	0.096	57.6
s_Allobaculum_sp_539	serum_7-Ketodeoxycholic acid	-0.061 (-0.096, -0.024)	<0.001	<0.001	-0.021 (-0.052, -0.002)	0.032	0.209	34
s_Allobaculum_sp_539	serum_3a,6b,7b,12a-Tetrahydroxy-5b-cholanoic acid	-0.061 (-0.102, -0.029)	<0.001	<0.001	-0.019 (-0.05, -0.002)	0.032	0.209	31.7
s_Bacteroidales_bacterium	intestinal_Cortisone	0.796 (0.444, 1.149)	<0.001	<0.001	0.076 (0.017, 0.436)	0.012	0.360	9.5
s_Bacteroidales_bacterium	intestinal_Docosahexaenoic acid	0.796 (0.472, 1.137)	<0.001	<0.001	0.195 (0.028, 0.486)	0.032	0.360	24.5
s_Bacteroidales_bacterium	intestinal_Ubiquinone-2	0.796 (0.434, 1.112)	<0.001	<0.001	0.334 (0.135, 0.721)	<0.001	<0.001	41.9
s_Bacteroidales_bacterium	serum_1,6-Digalloyl-beta-D-glucopyranose	0.796 (0.416, 1.193)	<0.001	<0.001	0.185 (0.003, 0.376)	0.048	0.360	23.3
s_Bacteroidales_bacterium	serum_PS(18:1(11Z)/20:0)	0.796 (0.408, 1.203)	<0.001	<0.001	0.302 (0.058, 0.558)	0.016	0.360	37.9
s_Muribaculaceae_bacterium_Isolate_080_Janvier	intestinal_Docosahexaenoic acid	-0.288 (-0.564, -0.056)	0.032	0.033	-0.251 (-0.522, -0.051)	0.012	0.048	87.3
s_Muribaculaceae_bacterium_Isolate_080_Janvier	intestinal_Docosanol	-0.288 (-0.656, -0.063)	0.020	0.028	-0.217 (-0.514, -0.043)	0.024	0.075	75.3
s_Muribaculaceae_bacterium_Isolate_080_Janvier	intestinal_gamma-Linolenic acid	-0.288 (-0.596, -0.068)	0.016	0.028	-0.134 (-0.429, -0.012)	0.032	0.096	46.6
s_Muribaculaceae_bacterium_Isolate_080_Janvier	serum_(±)-Tryptophan	-0.288 (-0.609, -0.055)	0.016	0.028	-0.227 (-0.48, -0.062)	0.008	0.036	78.9
s_Muribaculaceae_bacterium_Isolate_080_Janvier	serum_Arachidoyl Ethanolamide	-0.288 (-0.566, -0.04)	0.032	0.033	-0.241 (-0.472, -0.081)	0.008	0.036	83.7
s_Muribaculaceae_bacterium_Isolate_080_Janvier	serum_Phenyllactic acid	-0.288 (-0.566, -0.045)	0.020	0.028	-0.277 (-0.573, -0.102)	0.016	0.058	96.3
s_Muribaculaceae_bacterium_Isolate_080_Janvier	serum_Stearoylethanolamide	-0.288 (-0.636, -0.076)	0.004	0.028	-0.243 (-0.524, -0.1)	0.004	0.032	84.5
s_Muribaculaceae_bacterium_Isolate_080_Janvier	serum_Heptadecanoic acid	-0.288 (-0.563, -0.023)	0.036	0.036	-0.275 (-0.537, -0.125)	0.004	0.032	95.5

s_Muribaculaceae_bacterium_Isolate_080_Janvier	serum_Esterase	-0.288 (-0.594, -0.065)	0.008	0.028	-0.189 (-0.49, -0.061)	<0.001	<0.001	65.6
s_Muribaculaceae_bacterium_Isolate_080_Janvier	serum_Palmitoylethanolamide	-0.288 (-0.599, -0.039)	0.028	0.031	-0.238 (-0.459, -0.103)	0.008	0.036	82.6
s_Muribaculaceae_bacterium_Isolate_080_Janvier	serum_Glycocholic acid	-0.288 (-0.592, -0.048)	0.024	0.028	-0.233 (-0.467, -0.075)	0.004	0.032	80.9
s_Muribaculaceae_bacterium_Isolate_080_Janvier	serum_Palmitic acid	-0.288 (-0.605, -0.068)	0.012	0.028	-0.183 (-0.388, -0.035)	0.024	0.075	63.5
s_Muribaculaceae_bacterium_Isolate_080_Janvier	serum_PC(16:0/20:4(5Z,8Z,11Z,14Z))	-0.288 (-0.565, -0.068)	0.004	0.028	-0.202 (-0.453, -0.072)	0.008	0.036	70.1
s_Muribaculaceae_bacterium_Isolate_080_Janvier	serum_PC(16:0/18:1(9Z))	-0.288 (-0.587, -0.055)	0.008	0.028	-0.132 (-0.379, -0.017)	0.036	0.104	45.9
s_Muribaculaceae_bacterium_Isolate_080_Janvier	serum_1-Palmitoylphosphatidylcholine	-0.288 (-0.571, -0.055)	0.012	0.028	-0.213 (-0.435, -0.075)	0.008	0.036	73.9
s_Muribaculaceae_bacterium_Isolate_080_Janvier	serum_Sphinganine	-0.288 (-0.612, -0.053)	0.020	0.028	-0.199 (-0.359, -0.064)	0.008	0.036	69.2
s_Muribaculaceae_bacterium_Isolate_080_Janvier	serum_Deoxycholic acid	-0.288 (-0.578, -0.059)	0.024	0.028	-0.201 (-0.417, -0.038)	0.008	0.036	69.8
s_Muribaculaceae_bacterium_Isolate_080_Janvier	serum_Docosapentaenoic acid (22n-3)	-0.288 (-0.569, -0.047)	0.024	0.028	-0.218 (-0.422, -0.075)	0.004	0.032	75.9
s_Muribaculaceae_bacterium_Isolate_080_Janvier	serum_Anandamide	-0.288 (-0.562, -0.044)	0.028	0.031	-0.249 (-0.483, -0.098)	0.004	0.032	86.6
s_Muribaculaceae_bacterium_Isolate_080_Janvier	serum_1,6-Digalloyl-beta-D-glucopyranose	-0.288 (-0.559, -0.073)	0.004	0.028	-0.195 (-0.416, -0.072)	0.012	0.048	67.8
s_Muribaculaceae_bacterium_Isolate_080_Janvier	serum_1-Methylguanosine	-0.288 (-0.588, -0.064)	0.004	0.028	-0.2 (-0.422, -0.031)	0.024	0.075	69.5
s_Muribaculaceae_bacterium_Isolate_080_Janvier	serum_Retinal	-0.288 (-0.575, -0.057)	0.024	0.028	-0.231 (-0.459, -0.06)	0.004	0.032	80.4
s_Muribaculaceae_bacterium_Isolate_080_Janvier	serum_LysoPC(17:0/0:0)	-0.288 (-0.582, -0.057)	0.028	0.031	-0.158 (-0.37, -0.041)	0.016	0.058	54.8

s_Muribaculaceae_bacterium_Isolate_080_Janvier	serum_Glucosamine 6-phosphate	-0.288 (-0.575, -0.064)	0.020	0.028	-0.193 (-0.458, -0.016)	0.04	0.111	66.9
o_Coriobacteriales	intestinal_Docosahexaenoic acid	-0.203 (-0.292, -0.126)	<0.001	<0.001	-0.077 (-0.176, 0)	0.048	0.390	38.1
o_Coriobacteriales	intestinal_Ubiquinone-2	-0.203 (-0.29, -0.118)	<0.001	<0.001	-0.133 (-0.289, -0.059)	<0.001	<0.001	65.5
o_Coriobacteriales	serum_Phenyllactic acid	-0.203 (-0.3, -0.121)	<0.001	<0.001	-0.083 (-0.246, -0.011)	0.044	0.390	40.9
o_Coriobacteriales	serum_Retinal	-0.203 (-0.305, -0.123)	<0.001	<0.001	-0.086 (-0.223, -0.01)	0.024	0.390	42.3
o_Coriobacteriales	serum_PS(18:1(11Z)/20:0)	-0.203 (-0.294, -0.118)	<0.001	<0.001	-0.121 (-0.244, -0.045)	0.004	0.144	59.8
f_Odoribacteraceae	intestinal_Cer(d18:1/16:0)	0.318 (0.203, 0.442)	<0.001	<0.001	0.184 (0.057, 0.404)	<0.001	<0.001	58
f_Odoribacteraceae	intestinal_Ubiquinone-2	0.318 (0.182, 0.438)	<0.001	<0.001	0.212 (0.084, 0.435)	<0.001	<0.001	66.5
f_Odoribacteraceae	serum_Phenyllactic acid	0.318 (0.194, 0.446)	<0.001	<0.001	0.132 (0.013, 0.318)	0.044	0.220	41.5
f_Odoribacteraceae	serum_Heptadecanoic acid	0.318 (0.186, 0.443)	<0.001	<0.001	0.14 (0.021, 0.343)	0.028	0.220	44
f_Odoribacteraceae	serum_Esterase	0.318 (0.189, 0.42)	<0.001	<0.001	0.108 (0.005, 0.231)	0.044	0.220	34
f_Odoribacteraceae	serum_Palmitoylethanolamide	0.318 (0.209, 0.43)	<0.001	<0.001	0.138 (0.026, 0.361)	0.02	0.220	43.5
f_Odoribacteraceae	serum_Glycocholic acid	0.318 (0.192, 0.43)	<0.001	<0.001	0.132 (0.02, 0.332)	0.024	0.220	41.4
f_Odoribacteraceae	serum_1-Palmitoylphosphatidylcholine	0.318 (0.209, 0.447)	<0.001	<0.001	0.114 (0.004, 0.319)	0.04	0.220	35.8
f_Odoribacteraceae	serum_Sphinganine	0.318 (0.193, 0.416)	<0.001	<0.001	0.108 (0.013, 0.219)	0.028	0.220	34
f_Odoribacteraceae	serum_Anandamide	0.318 (0.194, 0.419)	<0.001	<0.001	0.137 (0.012, 0.369)	0.036	0.220	43.1
f_Odoribacteraceae	serum_Retinal	0.318 (0.193, 0.432)	<0.001	<0.001	0.133 (0.005, 0.325)	0.048	0.220	42
f_Odoribacteraceae	serum_PS(18:1(11Z)/20:0)	0.318 (0.198, 0.438)	<0.001	<0.001	0.183 (0.047, 0.347)	0.012	0.220	57.6
f_Odoribacteraceae	serum_LysoPC(17:0/0:0)	0.318 (0.214, 0.446)	<0.001	<0.001	0.096 (0.005, 0.286)	0.028	0.220	30.3
f_Odoribacteraceae	serum_7-Ketodeoxycholic acid	0.318 (0.185, 0.427)	<0.001	<0.001	0.104 (0.01, 0.275)	0.02	0.220	32.6
f_Odoribacteraceae	serum_Leukotriene C4	0.318 (0.181, 0.441)	<0.001	<0.001	0.113 (0.011, 0.277)	0.04	0.220	35.5
f_Coriobacteriaceae	intestinal_Docosahexaenoic acid	-0.207 (-0.308, -0.132)	<0.001	<0.001	-0.081 (-0.19, -0.014)	0.032	0.329	39.2
f_Coriobacteriaceae	intestinal_Ubiquinone-2	-0.207 (-0.298, -0.123)	<0.001	<0.001	-0.137 (-0.295, -0.063)	0.004	0.288	66.1
f_Coriobacteriaceae	serum_Sphingosine	-0.207 (-0.303, -0.124)	<0.001	<0.001	-0.077 (-0.17, -0.008)	0.044	0.329	37.1

f_Coriobacteriaceae	serum_Docosapentaenoic acid (22n-3)	-0.207 (-0.301, -0.125)	<0.001	<0.001	-0.076 (-0.236, -0.001)	0.048	0.329	36.8
f_Coriobacteriaceae	serum_Anandamide	-0.207 (-0.289, -0.121)	<0.001	<0.001	-0.086 (-0.249, -0.001)	0.048	0.329	41.6
f_Coriobacteriaceae	serum_Retinal	-0.207 (-0.305, -0.124)	<0.001	<0.001	-0.089 (-0.21, -0.004)	0.04	0.329	43
f_Coriobacteriaceae	serum_PS(18:1(11Z)/20:0)	-0.207 (-0.3, -0.126)	<0.001	<0.001	-0.124 (-0.275, -0.041)	0.008	0.288	59.7
s_Bacteroidales_bacterium_55_9	intestinal_Cer(d18:1/16:0)	0.173 (0.112, 0.233)	<0.001	<0.001	0.074 (0.011, 0.147)	0.024	0.864	42.8
s_Bacteroidales_bacterium_55_9	intestinal_Ubiquinone-2	0.173 (0.107, 0.233)	<0.001	<0.001	0.078 (0.029, 0.221)	0.004	0.288	45.3
s_Coriobacteriaceae_bacterium	intestinal_Cer(d18:1/16:0)	-0.104 (-0.144, -0.069)	<0.001	<0.001	-0.059 (-0.145, -0.002)	0.032	0.864	56.7
s_Coriobacteriaceae_bacterium	intestinal_Ubiquinone-2	-0.104 (-0.146, -0.067)	<0.001	<0.001	-0.055 (-0.172, -0.018)	0.008	0.576	52.9
s_Alistipes_senegalensis	intestinal_Cer(d18:1/16:0)	0.281 (0.175, 0.417)	<0.001	<0.001	0.163 (0.061, 0.502)	<0.001	<0.001	58.2
s_Alistipes_senegalensis	intestinal_Ubiquinone-2	0.281 (0.163, 0.43)	<0.001	<0.001	0.249 (0.115, 0.538)	<0.001	<0.001	88.6
s_Alistipes_senegalensis	serum_Dimyristoylphosphatidylcholine, DL	0.281 (0.173, 0.449)	<0.001	<0.001	0.062 (0.008, 0.262)	0.028	0.288	22.2
s_Alistipes_senegalensis	serum_Glucose 6-phosphate	0.281 (0.165, 0.442)	<0.001	<0.001	0.092 (0.016, 0.255)	0.016	0.192	32.8
s_Alistipes_senegalensis	serum_PS(18:1(11Z)/20:0)	0.281 (0.16, 0.423)	<0.001	<0.001	0.178 (0.06, 0.382)	0.008	0.144	63.2
s_Alistipes_senegalensis	serum_7-Ketodeoxycholic acid	0.281 (0.166, 0.447)	<0.001	<0.001	0.087 (0.015, 0.262)	0.004	0.096	30.9
s_Alistipes_senegalensis	serum_3a,6b,7b,12a-Tetrahydroxy-5b-cholanoic acid	0.281 (0.159, 0.437)	<0.001	<0.001	0.08 (0.011, 0.246)	0.012	0.173	28.5
s_Alistipes_senegalensis	serum_Leukotriene C4	0.281 (0.167, 0.431)	<0.001	<0.001	0.09 (0.014, 0.227)	0.036	0.314	32.2
s_Alistipes_finegoldii	intestinal_Cer(d18:1/16:0)	0.256 (0.163, 0.407)	<0.001	<0.001	0.145 (0.063, 0.415)	<0.001	<0.001	56.5
s_Alistipes_finegoldii	intestinal_Ubiquinone-2	0.256 (0.148, 0.392)	<0.001	<0.001	0.227 (0.102, 0.513)	<0.001	<0.001	88.4
s_Alistipes_finegoldii	serum_Dimyristoylphosphatidylcholine, DL	0.256 (0.159, 0.432)	<0.001	<0.001	0.055 (0.009, 0.256)	0.024	0.288	21.6
s_Alistipes_finegoldii	serum_Glucose 6-phosphate	0.256 (0.147, 0.423)	<0.001	<0.001	0.082 (0.017, 0.253)	0.016	0.230	31.9

s_Alistipes_finegoldii	serum_PS(18:1(11Z)/20:0)	0.256 (0.155, 0.4)	<0.001	<0.001	0.162 (0.043, 0.388)	0.004	0.096	63.1
s_Alistipes_finegoldii	serum_7-Ketodeoxycholic acid	0.256 (0.153, 0.402)	<0.001	<0.001	0.077 (0.009, 0.243)	0.016	0.230	30
s_Alistipes_finegoldii	serum_3a,6b,7b,12a-Tetrahydroxy-5b-cholanoic acid	0.256 (0.145, 0.408)	<0.001	<0.001	0.071 (0.004, 0.239)	0.028	0.288	27.7
s_Alistipes_finegoldii	serum_Leukotriene C4	0.256 (0.158, 0.402)	<0.001	<0.001	0.081 (0.008, 0.176)	0.04	0.360	31.7
g_Odoribacter	intestinal_Cer(d18:1/16:0)	0.317 (0.198, 0.441)	<0.001	<0.001	0.18 (0.057, 0.393)	<0.001	<0.001	56.8
g_Odoribacter	intestinal_Ubiquinone-2	0.317 (0.2, 0.453)	<0.001	<0.001	0.216 (0.101, 0.484)	<0.001	<0.001	68
g_Odoribacter	serum_(±)-Tryptophan	0.317 (0.186, 0.463)	0.004	0.004	0.129 (0.005, 0.359)	0.048	0.197	40.7
g_Odoribacter	serum_Arachidoyl Ethanolamide	0.317 (0.173, 0.451)	<0.001	<0.001	0.127 (0.015, 0.298)	0.044	0.197	40.1
g_Odoribacter	serum_Phenyllactic acid	0.317 (0.202, 0.446)	<0.001	<0.001	0.135 (0.013, 0.324)	0.036	0.197	42.4
g_Odoribacter	serum_Heptadecanoic acid	0.317 (0.201, 0.43)	<0.001	<0.001	0.142 (0.02, 0.345)	0.032	0.197	44.8
g_Odoribacter	serum_Esterase	0.317 (0.18, 0.446)	<0.001	<0.001	0.109 (0.008, 0.261)	0.036	0.197	34.5
g_Odoribacter	serum_1-Palmitoylphosphatidylcholine	0.317 (0.184, 0.429)	<0.001	<0.001	0.116 (0.016, 0.317)	0.036	0.197	36.4
g_Odoribacter	serum_Sphinganine	0.317 (0.2, 0.425)	<0.001	<0.001	0.11 (0.015, 0.251)	0.02	0.197	34.6
g_Odoribacter	serum_Docosapentaenoic acid (22n-3)	0.317 (0.177, 0.434)	<0.001	<0.001	0.122 (0.003, 0.344)	0.048	0.197	38.4
g_Odoribacter	serum_Anandamide	0.317 (0.179, 0.44)	<0.001	<0.001	0.14 (0.024, 0.362)	0.024	0.197	44
g_Odoribacter	serum_1,6-Digalloyl-beta-D-glucopyranose	0.317 (0.193, 0.44)	<0.001	<0.001	0.111 (0.023, 0.247)	0.032	0.197	34.9
g_Odoribacter	serum_Glucose 6-phosphate	0.317 (0.183, 0.445)	<0.001	<0.001	0.124 (0.016, 0.3)	0.032	0.197	39
g_Odoribacter	serum_Retinal	0.317 (0.172, 0.431)	<0.001	<0.001	0.137 (0.013, 0.345)	0.044	0.197	43
g_Odoribacter	serum_PS(18:1(11Z)/20:0)	0.317 (0.192, 0.425)	<0.001	<0.001	0.187 (0.06, 0.345)	0.016	0.197	58.8
g_Odoribacter	serum_LysoPC(17:0/0:0)	0.317 (0.186, 0.448)	<0.001	<0.001	0.097 (0.01, 0.301)	0.032	0.197	30.6
g_Odoribacter	serum_Leukotriene C4	0.317 (0.174, 0.422)	<0.001	<0.001	0.113 (0.013, 0.261)	0.032	0.197	35.5
s_Alistipes_sp_58_9_plus	intestinal_Cer(d18:1/16:0)	0.165 (0.103, 0.226)	<0.001	<0.001	0.069 (0.023, 0.126)	0.024	0.864	42.1
s_Alistipes_sp_58_9_plus	intestinal_Ubiquinone-2	0.165 (0.112, 0.226)	<0.001	<0.001	0.074 (0.026, 0.208)	0.012	0.864	45

s_Alistipes_sp_	intestinal_Cer(d18:1/16:0)	0.191 (0.109, 0.283)	<0.001	<0.001	0.116 (0.05, 0.347)	0.004	0.144	60.9
s_Alistipes_sp_	intestinal_Ubiquinone-2	0.191 (0.109, 0.298)	<0.001	<0.001	0.139 (0.055, 0.298)	0.004	0.144	73
s_Bacteroides_caecimuris	intestinal_Cer(d18:1/16:0)	0.389 (0.266, 0.491)	<0.001	<0.001	0.188 (0.04, 0.352)	0.02	0.432	48.5
s_Bacteroides_caecimuris	intestinal_Ubiquinone-2	0.389 (0.27, 0.5)	<0.001	<0.001	0.173 (0.081, 0.371)	0.008	0.288	44.4
s_Bacteroides_caecimuris	serum_PS(18:1(11Z)/20:0)	0.389 (0.266, 0.505)	<0.001	<0.001	0.155 (0.013, 0.316)	0.04	0.576	39.9
s_Bacteroides_caecimuris	serum_Leukotriene C4	0.389 (0.255, 0.491)	<0.001	<0.001	0.089 (0.013, 0.233)	0.024	0.432	23
s_Alistipes_onderdonkii	intestinal_Cer(d18:1/16:0)	0.249 (0.147, 0.39)	<0.001	<0.001	0.14 (0.053, 0.367)	<0.001	<0.001	56.1
s_Alistipes_onderdonkii	intestinal_PC(16:0/18:1(9Z))	0.249 (0.151, 0.385)	<0.001	<0.001	0.083 (0, 0.234)	0.048	0.384	33.5
s_Alistipes_onderdonkii	intestinal_Ubiquinone-2	0.249 (0.138, 0.393)	<0.001	<0.001	0.215 (0.085, 0.452)	0.004	0.144	86.3
s_Alistipes_onderdonkii	serum_Dimyristoylphosphatidylcholine, DL	0.249 (0.15, 0.374)	<0.001	<0.001	0.054 (0.007, 0.23)	0.02	0.240	21.5
s_Alistipes_onderdonkii	serum_Glucose 6-phosphate	0.249 (0.144, 0.402)	<0.001	<0.001	0.079 (0.013, 0.206)	0.036	0.324	31.6
s_Alistipes_onderdonkii	serum_PS(18:1(11Z)/20:0)	0.249 (0.153, 0.378)	<0.001	<0.001	0.154 (0.053, 0.396)	0.008	0.192	62.1
s_Alistipes_onderdonkii	serum_7-Ketodeoxycholic acid	0.249 (0.141, 0.399)	<0.001	<0.001	0.075 (0.01, 0.241)	0.016	0.230	30.1
s_Alistipes_onderdonkii	serum_3a,6b,7b,12a-Tetrahydroxy-5b-cholanoic acid	0.249 (0.159, 0.4)	<0.001	<0.001	0.069 (0.009, 0.237)	0.016	0.230	27.7
s_Alistipes_onderdonkii	serum_Leukotriene C4	0.249 (0.155, 0.382)	<0.001	<0.001	0.078 (0.01, 0.176)	0.036	0.324	31.3
s_Alistipes_shahii	intestinal_Cer(d18:1/16:0)	0.33 (0.194, 0.488)	<0.001	<0.001	0.185 (0.073, 0.518)	<0.001	<0.001	56.2
s_Alistipes_shahii	intestinal_Ubiquinone-2	0.33 (0.188, 0.498)	<0.001	<0.001	0.269 (0.109, 0.555)	0.004	0.144	81.5
s_Alistipes_shahii	serum_Dimyristoylphosphatidylcholine, DL	0.33 (0.196, 0.507)	<0.001	<0.001	0.07 (0.003, 0.282)	0.036	0.370	21.3
s_Alistipes_shahii	serum_Glucose 6-phosphate	0.33 (0.193, 0.5)	<0.001	<0.001	0.103 (0.017, 0.292)	0.016	0.230	31.1
s_Alistipes_shahii	serum_PS(18:1(11Z)/20:0)	0.33 (0.199, 0.498)	<0.001	<0.001	0.199 (0.061, 0.457)	0.012	0.230	60.3
s_Alistipes_shahii	serum_7-Ketodeoxycholic acid	0.33 (0.182, 0.493)	<0.001	<0.001	0.099 (0.009, 0.313)	0.016	0.230	30.1

s_Alistipes_shahii	serum_3a,6b,7b,12a-Tetrahydroxy-5b-cholanoic acid	0.33 (0.199, 0.511)	<0.001	<0.001	0.091 (0.004, 0.312)	0.036	0.370	27.7
s_Alistipes_shahii	serum_Leukotriene C4	0.33 (0.193, 0.505)	<0.001	<0.001	0.099 (0.004, 0.255)	0.044	0.396	30.1
s_Alistipes_timonensis	intestinal_Cer(d18:1/16:0)	0.266 (0.162, 0.416)	<0.001	<0.001	0.153 (0.055, 0.421)	<0.001	<0.001	57.6
s_Alistipes_timonensis	intestinal_PC(16:0/18:1(9Z))	0.266 (0.156, 0.425)	<0.001	<0.001	0.093 (0.004, 0.265)	0.04	0.352	34.9
s_Alistipes_timonensis	intestinal_Ubiquinone-2	0.266 (0.161, 0.417)	<0.001	<0.001	0.24 (0.101, 0.526)	<0.001	<0.001	90.5
s_Alistipes_timonensis	serum_Dimyristoylphosphatidylcholine, DL	0.266 (0.158, 0.405)	<0.001	<0.001	0.058 (0.006, 0.234)	0.024	0.288	21.9
s_Alistipes_timonensis	serum_Glucose 6-phosphate	0.266 (0.157, 0.417)	<0.001	<0.001	0.086 (0.016, 0.244)	0.028	0.288	32.6
s_Alistipes_timonensis	serum_PS(18:1(11Z)/20:0)	0.266 (0.162, 0.41)	<0.001	<0.001	0.168 (0.058, 0.447)	0.008	0.144	63.4
s_Alistipes_timonensis	serum_7-Ketodeoxycholic acid	0.266 (0.162, 0.436)	<0.001	<0.001	0.081 (0.013, 0.269)	0.016	0.230	30.4
s_Alistipes_timonensis	serum_3a,6b,7b,12a-Tetrahydroxy-5b-cholanoic acid	0.266 (0.153, 0.393)	<0.001	<0.001	0.075 (0.01, 0.218)	0.004	0.096	28.1
s_Alistipes_timonensis	serum_Leukotriene C4	0.266 (0.154, 0.415)	<0.001	<0.001	0.086 (0.004, 0.203)	0.044	0.352	32.3
s_Alistipes_sp_An66	intestinal_Cer(d18:1/16:0)	0.237 (0.14, 0.356)	<0.001	<0.001	0.132 (0.054, 0.346)	<0.001	<0.001	55.8
s_Alistipes_sp_An66	intestinal_PC(16:0/18:1(9Z))	0.237 (0.134, 0.373)	<0.001	<0.001	0.079 (0.002, 0.23)	0.04	0.320	33.3
s_Alistipes_sp_An66	intestinal_Ubiquinone-2	0.237 (0.142, 0.382)	<0.001	<0.001	0.197 (0.089, 0.48)	<0.001	<0.001	83.1
s_Alistipes_sp_An66	serum_Dimyristoylphosphatidylcholine, DL	0.237 (0.14, 0.382)	<0.001	<0.001	0.051 (0.008, 0.219)	0.02	0.216	21.6
s_Alistipes_sp_An66	serum_Glucose 6-phosphate	0.237 (0.13, 0.378)	<0.001	<0.001	0.075 (0.015, 0.214)	0.008	0.192	31.5
s_Alistipes_sp_An66	serum_PS(18:1(11Z)/20:0)	0.237 (0.152, 0.369)	<0.001	<0.001	0.141 (0.048, 0.34)	0.012	0.216	59.3
s_Alistipes_sp_An66	serum_7-Ketodeoxycholic acid	0.237 (0.139, 0.402)	<0.001	<0.001	0.071 (0.012, 0.221)	0.02	0.216	30.1

	s_Alistipes_sp_An66	serum_3a,6b,7b,12a-Tetrahydroxy-5b-cholanoic acid	0.237 (0.139, 0.37)	<0.001	<0.001	0.066 (0.008, 0.216)	0.024	0.216	27.8
	s_Alistipes_sp_An66	serum_Leukotriene C4	0.237 (0.139, 0.403)	<0.001	<0.001	0.073 (0.014, 0.169)	0.024	0.216	31
	s_Duncaniella_muris	intestinal_Cortisone	-0.697 (-1.231, -0.193)	0.016	0.020	-0.253 (-1.287, -0.039)	0.032	0.092	36.3
	s_Duncaniella_muris	intestinal_Docosanol	-0.697 (-1.194, -0.127)	0.020	0.023	-0.644 (-1.716, -0.034)	0.044	0.099	92.4
	s_Duncaniella_muris	intestinal_gamma-Linolenic acid	-0.697 (-1.291, -0.204)	0.004	0.018	-0.454 (-1.06, -0.147)	0.004	0.038	65.2
	s_Duncaniella_muris	intestinal_PC(16:0/18:1(9Z))	-0.697 (-1.225, -0.142)	0.024	0.025	-0.53 (-1.091, -0.159)	0.008	0.038	76.1
	s_Duncaniella_muris	serum_(±)-Tryptophan	-0.697 (-1.205, -0.223)	0.016	0.020	-0.559 (-1.044, -0.128)	0.02	0.060	80.2
	s_Duncaniella_muris	serum_Esterase	-0.697 (-1.191, -0.091)	0.032	0.032	-0.694 (-1.409, -0.23)	0.012	0.043	99.6
	s_Duncaniella_muris	serum_Palmitic acid	-0.697 (-1.188, -0.201)	0.016	0.020	-0.647 (-1.42, -0.193)	0.012	0.043	92.8
	s_Duncaniella_muris	serum_PC(16:0/20:4(5Z,8Z,11Z,14Z))	-0.697 (-1.278, -0.193)	0.004	0.018	-0.632 (-1.633, -0.187)	<0.001	<0.001	90.7
	s_Duncaniella_muris	serum_PC(16:0/18:1(9Z))	-0.697 (-1.215, -0.184)	0.016	0.020	-0.485 (-1.448, -0.036)	0.036	0.093	69.5
	s_Duncaniella_muris	serum_Deoxycholic acid	-0.697 (-1.198, -0.213)	0.008	0.018	-0.648 (-1.392, -0.051)	0.044	0.099	92.9
	s_Duncaniella_muris	serum_Dimyristoylphosphatidylcholine, DL	-0.697 (-1.187, -0.158)	0.016	0.020	-0.338 (-1.114, -0.08)	0.012	0.043	48.6
	s_Duncaniella_muris	serum_Glucose 6-phosphate	-0.697 (-1.239, -0.226)	0.008	0.018	-0.359 (-0.983, -0.065)	0.008	0.038	51.4
	s_Duncaniella_muris	serum_1-Methylguanosine	-0.697 (-1.265, -0.209)	0.016	0.020	-0.629 (-1.269, -0.041)	0.036	0.093	90.2
	s_Duncaniella_muris	serum_LysoPC(17:0/0:0)	-0.697 (-1.234, -0.166)	0.008	0.018	-0.591 (-1.229, -0.164)	0.008	0.038	84.8
	s_Duncaniella_muris	serum_Glucosamine 6-phosphate	-0.697 (-1.297, -0.207)	0.012	0.020	-0.557 (-1.166, -0.002)	0.048	0.105	79.9
	s_Duncaniella_muris	serum_Leukotriene C4	-0.697 (-1.213, -0.184)	0.016	0.020	-0.421 (-1.213, -0.082)	0.008	0.038	60.3
brain_7alpha-Hydroxycholesterol	f_Erysipelotrichaceae	serum_Glucosamine 6-phosphate	0.093 (0.019, 0.18)	0.024	0.037	0.042 (0.002, 0.132)	0.028	0.569	44.6
	c_Erysipelotrichia	serum_Glucosamine 6-phosphate	0.098 (0.016, 0.187)	0.028	0.045	0.044 (0.002, 0.129)	0.04	0.515	45.2
	o_Erysipelotrichales	intestinal_PE(P-16:0/20:4(5Z,8Z,11Z,14Z))	0.098 (0.009, 0.197)	0.036	0.042	0.076 (0.011, 0.175)	0.024	0.516	77.4

	s_Erysipelotrichaceae_bacterium	intestinal_PE(P-16:0/20:4(5Z,8Z,11Z,14Z))	0.101 (0.011, 0.199)	0.028	0.062	0.078 (0.003, 0.187)	0.044	0.488	78
	f_Rikenellaceae	intestinal_Docosanol	-0.132 (-0.221, -0.041)	0.004	0.008	-0.092 (-0.201, -0.011)	0.028	0.892	69.6
	g_Duncaniella	intestinal_Docosanol	0.254 (0.111, 0.442)	<0.001	<0.001	0.153 (0.009, 0.374)	0.032	0.657	60.2
	g_Heminiphilus	intestinal_Docosanol	-0.106 (-0.208, -0.004)	0.048	0.061	-0.075 (-0.167, -0.012)	0.004	0.288	70.8
	g_Heminiphilus	intestinal_Staurosporine	-0.106 (-0.216, -0.015)	0.024	0.061	-0.067 (-0.187, 0)	0.048	0.398	63.9
	s_Heminiphilus_faecis	intestinal_Docosanol	-0.106 (-0.214, -0.01)	0.032	0.065	-0.075 (-0.184, -0.016)	0.02	0.413	70.8
	s_Heminiphilus_faecis	intestinal_Estrogen	-0.106 (-0.213, -0.004)	0.040	0.065	-0.069 (-0.207, -0.004)	0.036	0.413	65.3
	s_Duncaniella_dubosii	intestinal_Docosanol	0.17 (0.069, 0.305)	0.004	0.005	0.101 (0.003, 0.259)	0.044	0.630	59.5
	g_Faecalibaculum	intestinal_Docosanol	0.059 (0.01, 0.114)	0.020	0.035	0.049 (0.006, 0.105)	0.032	0.624	83.4
	s_Faecalibaculum_rodentium	intestinal_Docosanol	0.059 (0.009, 0.116)	0.024	0.032	0.049 (0.003, 0.105)	0.028	0.627	83.4
	g_Allobaculum	intestinal_Docosanol	0.031 (0.008, 0.061)	0.008	0.012	0.025 (0.005, 0.062)	0.008	0.576	81.5
	s_Allobaculum_sp_539	intestinal_Docosanol	0.028 (0.008, 0.051)	0.004	0.005	0.02 (0.002, 0.046)	0.036	0.766	73.4
	s_Muribaculaceae_bacterium_Isolate_080_Janvier	intestinal_Docosanol	0.202 (0.074, 0.358)	0.012	0.012	0.124 (0.003, 0.337)	0.048	0.666	61.4
	s_Bacteroidales_bacterium_55_9	intestinal_Docosanol	-0.055 (-0.113, -0.011)	0.020	0.038	-0.054 (-0.119, -0.011)	0.008	0.576	98.8
	s_Alistipes_finegoldii	intestinal_Docosanol	-0.111 (-0.186, -0.036)	0.008	0.012	-0.079 (-0.185, -0.002)	0.044	0.913	71.5
	s_Alistipes_shahii	intestinal_Docosanol	-0.142 (-0.231, -0.036)	0.012	0.013	-0.095 (-0.234, -0.002)	0.048	0.960	67.1
brain_Cholestenone	s_Erysipelotrichaceae_bacterium	serum_Alkergot	0.083 (0.001, 0.183)	0.048	0.128	0.05 (0.004, 0.155)	0.036	0.540	59.6
	s_Allobaculum_sp_539	intestinal_Docosanol	0.022 (0.002, 0.044)	0.024	0.041	0.018 (0.001, 0.046)	0.04	0.777	83.9
	s_Alistipes_onderdonkii	intestinal_Docosanol	-0.08 (-0.147, -0.006)	0.032	0.063	-0.075 (-0.189, -0.002)	0.048	0.883	94
brain_ADP-glucose	f_Erysipelotrichaceae	intestinal_(±)-2-Hydroxy-4-(methylthio)butanoic acid	0.439 (0.22, 0.62)	<0.001	<0.001	0.106 (0.003, 0.23)	0.04	0.568	24.2
	c_Erysipelotrichia	intestinal_alpha-Linolenic acid	0.473 (0.245, 0.677)	<0.001	<0.001	0.077 (0.012, 0.373)	0.036	0.676	16.3
	o_Erysipelotrichales	intestinal_(±)-2-Hydroxy-4-(methylthio)butanoic acid	0.473 (0.246, 0.631)	<0.001	<0.001	0.116 (0.008, 0.247)	0.032	0.668	24.5
	s_Erysipelotrichaceae_bacterium	intestinal_(±)-2-Hydroxy-4-(methylthio)butanoic acid	0.436 (0.182, 0.627)	<0.001	<0.001	0.135 (0.014, 0.276)	0.024	0.907	31

s_Muribaculaceae_bacterium_Isolate_037_Harlan	intestinal_(±)-2-Hydroxy-4-(methylthio)butanoic acid	-1.258 (-1.898, -0.513)	<0.001	<0.001	-0.371 (-0.906, -0.081)	0.012	0.864	29.5
f_Rikenellaceae	intestinal_3a,6a,7b-Trihydroxy-5b-cholanoic acid	-0.443 (-0.808, -0.153)	<0.001	<0.001	-0.108 (-0.319, -0.011)	0.028	0.453	24.5
f_Rikenellaceae	intestinal_alpha-Linolenic acid	-0.443 (-0.836, -0.15)	<0.001	<0.001	-0.116 (-0.554, -0.009)	0.04	0.453	26.1
f_Rikenellaceae	intestinal_Staurosporine	-0.443 (-0.791, -0.16)	<0.001	<0.001	-0.106 (-0.285, -0.003)	0.044	0.453	23.8
f_Rikenellaceae	intestinal_Stearic acid	-0.443 (-0.783, -0.153)	<0.001	<0.001	-0.148 (-0.367, -0.009)	0.032	0.453	33.5
f_Rikenellaceae	intestinal_9-Hpode	-0.443 (-0.834, -0.174)	<0.001	<0.001	-0.158 (-0.457, -0.017)	0.036	0.453	35.7
g_Alistipes	intestinal_alpha-Linolenic acid	-0.377 (-0.651, -0.141)	<0.001	<0.001	-0.099 (-0.407, -0.009)	0.04	0.468	26.3
g_Alistipes	intestinal_N2-gamma-Glutamylglutamine	-0.377 (-0.704, -0.128)	<0.001	<0.001	-0.121 (-0.285, -0.006)	0.024	0.468	32.2
g_Alistipes	intestinal_Stearic acid	-0.377 (-0.68, -0.165)	<0.001	<0.001	-0.128 (-0.348, -0.003)	0.036	0.468	34.1
g_Alistipes	intestinal_9-Hpode	-0.377 (-0.686, -0.167)	<0.001	<0.001	-0.135 (-0.337, -0.002)	0.04	0.468	35.7
s_Prevotella_sp_PMUR	intestinal_(±)-2-Hydroxy-4-(methylthio)butanoic acid	-0.539 (-0.705, -0.268)	<0.001	<0.001	-0.104 (-0.236, -0.019)	0.02	0.869	19.2
g_Duncaniella	intestinal_3a,6a,7b-Trihydroxy-5b-cholanoic acid	0.63 (0.134, 1.328)	0.008	0.024	0.339 (0.057, 1.139)	0.008	0.346	53.8
g_Duncaniella	intestinal_alpha-Linolenic acid	0.63 (0.098, 1.323)	0.012	0.024	0.3 (0.052, 1.288)	0.024	0.346	47.6
g_Duncaniella	intestinal_N2-gamma-Glutamylglutamine	0.63 (0.093, 1.344)	0.004	0.024	0.297 (0.045, 0.693)	0.016	0.346	47.1
g_Duncaniella	intestinal_Staurosporine	0.63 (0.143, 1.273)	0.008	0.024	0.325 (0.038, 0.956)	0.024	0.346	51.6
g_Duncaniella	intestinal_Indoxyl sulfate	0.63 (0.131, 1.316)	0.008	0.024	0.281 (0.01, 0.708)	0.044	0.453	44.5
g_Duncaniella	intestinal_PE(P-18:0/20:4(5Z,8Z,11Z,14Z))	0.63 (0.189, 1.361)	<0.001	<0.001	0.199 (0.005, 0.55)	0.036	0.432	31.6
g_Duncaniella	intestinal_9-Hpode	0.63 (0.09, 1.362)	0.012	0.024	0.292 (0.023, 0.817)	0.02	0.346	46.4
g_Heminiphilus	intestinal_alpha-Linolenic acid	-0.662 (-0.887, -0.391)	<0.001	<0.001	-0.087 (-0.372, -0.006)	0.04	0.960	13.2

g_Heminiphilus	intestinal_N2-gamma-Glutamylglutamine	-0.662 (-0.934, -0.363)	<0.001	<0.001	-0.12 (-0.411, -0.009)	0.04	0.960	18.1
g_Heminiphilus	intestinal_9-Hpode	-0.662 (-0.899, -0.363)	<0.001	<0.001	-0.126 (-0.39, -0.009)	0.024	0.960	19
s_Heminiphilus_faecis	intestinal_alpha-Linolenic acid	-0.662 (-0.879, -0.33)	<0.001	<0.001	-0.087 (-0.45, -0.008)	0.04	0.960	13.2
s_Heminiphilus_faecis	intestinal_N2-gamma-Glutamylglutamine	-0.662 (-0.876, -0.396)	<0.001	<0.001	-0.12 (-0.381, -0.016)	0.012	0.864	18.1
s_Heminiphilus_faecis	intestinal_9-Hpode	-0.662 (-0.913, -0.327)	<0.001	<0.001	-0.126 (-0.396, -0.006)	0.032	0.960	19
s_Duncaniella_dubosii	intestinal_3a,6a,7b-Trihydroxy-5b-cholanoic acid	0.515 (0.162, 1.015)	0.004	0.006	0.216 (0.012, 0.69)	0.036	0.576	41.9
s_Duncaniella_dubosii	intestinal_N2-gamma-Glutamylglutamine	0.515 (0.156, 1.03)	0.004	0.006	0.2 (0.021, 0.47)	0.036	0.576	38.8
s_Duncaniella_dubosii	intestinal_Staurosporine	0.515 (0.157, 1.025)	<0.001	<0.001	0.206 (0.007, 0.599)	0.04	0.576	40
s_Duncaniella_dubosii	intestinal_Stearic acid	0.515 (0.15, 1.018)	0.004	0.006	0.175 (0.003, 0.427)	0.048	0.576	34
s_Duncaniella_dubosii	intestinal_9-Hpode	0.515 (0.112, 0.99)	<0.001	<0.001	0.206 (0.03, 0.549)	0.024	0.576	40
s_Bacteroidales_bacterium	intestinal_(±)-2-Hydroxy-4-(methylthio)butanoic acid	-1.19 (-1.749, -0.469)	<0.001	<0.001	-0.353 (-0.808, -0.089)	0.016	0.936	29.6
s_Muribaculaceae_bacterium_Isolate_080_Janvier	intestinal_3a,6a,7b-Trihydroxy-5b-cholanoic acid	0.569 (0.17, 1.217)	0.004	0.014	0.281 (0.015, 0.857)	0.04	0.524	49.5
s_Muribaculaceae_bacterium_Isolate_080_Janvier	intestinal_alpha-Linolenic acid	0.569 (0.115, 1.176)	0.008	0.014	0.221 (0.015, 0.894)	0.04	0.524	38.8
s_Muribaculaceae_bacterium_Isolate_080_Janvier	intestinal_N2-gamma-Glutamylglutamine	0.569 (0.141, 1.126)	0.008	0.014	0.247 (0.029, 0.597)	0.024	0.524	43.3
s_Muribaculaceae_bacterium_Isolate_080_Janvier	intestinal_Staurosporine	0.569 (0.138, 1.182)	0.008	0.014	0.269 (0.03, 0.781)	0.024	0.524	47.2
s_Muribaculaceae_bacterium_Isolate_080_Janvier	intestinal_Stearic acid	0.569 (0.139, 1.219)	0.020	0.021	0.194 (0.004, 0.517)	0.048	0.524	34.1
s_Muribaculaceae_bacterium_Isolate_080_Janvier	intestinal_9-Hpode	0.569 (0.147, 1.158)	0.004	0.014	0.248 (0.022, 0.722)	0.02	0.524	43.6

s_Muribaculaceae_bacterium_Isolate_002_NCI	intestinal_alpha-Linolenic acid	-0.643 (-1.314, -0.216)	<0.001	<0.001	-0.157 (-0.633, -0.051)	0.016	0.992	24.5
s_Muribaculaceae_bacterium_Isolate_002_NCI	intestinal_(±)-2-Hydroxy-4-(methylthio)butanoic acid	-0.643 (-1.241, -0.26)	<0.001	<0.001	-0.279 (-0.572, -0.018)	0.048	0.992	43.4
o_Coriobacteriales	intestinal_Stearic acid	0.196 (0.008, 0.4)	0.036	0.074	0.1 (0.004, 0.233)	0.032	0.731	50.8
f_Odoribacteraceae	intestinal_alpha-Linolenic acid	-0.49 (-0.86, -0.142)	<0.001	<0.001	-0.122 (-0.389, -0.017)	0.02	0.922	25
f_Odoribacteraceae	intestinal_(±)-2-Hydroxy-4-(methylthio)butanoic acid	-0.49 (-0.882, -0.136)	<0.001	<0.001	-0.151 (-0.321, -0.009)	0.04	0.922	30.7
s_Bacteroidales_bacterium_55_9	intestinal_alpha-Linolenic acid	-0.231 (-0.365, -0.102)	<0.001	<0.001	-0.051 (-0.187, -0.01)	0.024	0.112	22
s_Bacteroidales_bacterium_55_9	intestinal_N2-gamma-Glutamylglutamine	-0.231 (-0.381, -0.094)	<0.001	<0.001	-0.064 (-0.134, -0.001)	0.048	0.150	27.6
s_Bacteroidales_bacterium_55_9	intestinal_Stearic acid	-0.231 (-0.371, -0.11)	0.004	0.004	-0.064 (-0.202, -0.002)	0.036	0.130	27.4
s_Bacteroidales_bacterium_55_9	intestinal_(±)-2-Hydroxy-4-(methylthio)butanoic acid	-0.231 (-0.375, -0.12)	<0.001	<0.001	-0.077 (-0.149, -0.006)	0.024	0.112	33.2
s_Bacteroidales_bacterium_55_9	intestinal_9-Hpode	-0.231 (-0.389, -0.105)	<0.001	<0.001	-0.066 (-0.181, -0.004)	0.028	0.112	28.7
s_Coriobacteriaceae_bacterium	intestinal_Stearic acid	0.124 (0.044, 0.22)	0.004	0.004	0.045 (0.004, 0.121)	0.02	0.346	36.6
s_Coriobacteriaceae_bacterium	intestinal_(±)-2-Hydroxy-4-(methylthio)butanoic acid	0.124 (0.047, 0.221)	<0.001	<0.001	0.067 (0.007, 0.128)	0.024	0.346	54.5
s_Alistipes_senegalensis	intestinal_3a,6a,7b-Trihydroxy-5b-cholanoic acid	-0.382 (-0.7, -0.151)	<0.001	<0.001	-0.103 (-0.255, -0.001)	0.04	0.396	27
s_Alistipes_senegalensis	intestinal_alpha-Linolenic acid	-0.382 (-0.736, -0.14)	0.004	0.004	-0.11 (-0.444, -0.015)	0.032	0.396	28.9
s_Alistipes_senegalensis	intestinal_N2-gamma-Glutamylglutamine	-0.382 (-0.765, -0.137)	<0.001	<0.001	-0.136 (-0.302, -0.013)	0.036	0.396	35.7
s_Alistipes_senegalensis	intestinal_Staurosporine	-0.382 (-0.739, -0.148)	<0.001	<0.001	-0.1 (-0.259, -0.001)	0.044	0.396	26.3
s_Alistipes_senegalensis	intestinal_Stearic acid	-0.382 (-0.768, -0.128)	0.004	0.004	-0.145 (-0.378, -0.007)	0.036	0.396	37.9

s_Alistipes_senegalensis	intestinal_9-Hpode	-0.382 (-0.744, -0.141)	0.008	0.008	-0.154 (-0.411, -0.012)	0.02	0.396	40.4
s_Alistipes_finegoldii	intestinal_3a,6a,7b-Trihydroxy-5b-cholanoic acid	-0.339 (-0.642, -0.105)	<0.001	<0.001	-0.094 (-0.288, -0.002)	0.04	0.416	27.8
s_Alistipes_finegoldii	intestinal_alpha-Linolenic acid	-0.339 (-0.72, -0.114)	<0.001	<0.001	-0.104 (-0.452, -0.006)	0.048	0.416	30.5
s_Alistipes_finegoldii	intestinal_N2-gamma-Glutamylglutamine	-0.339 (-0.688, -0.11)	<0.001	<0.001	-0.124 (-0.273, -0.002)	0.044	0.416	36.7
s_Alistipes_finegoldii	intestinal_Staurosporine	-0.339 (-0.679, -0.122)	<0.001	<0.001	-0.091 (-0.239, -0.002)	0.04	0.416	27
s_Alistipes_finegoldii	intestinal_Indoxyl sulfate	-0.339 (-0.646, -0.121)	<0.001	<0.001	-0.102 (-0.273, -0.007)	0.044	0.416	30.1
s_Alistipes_finegoldii	intestinal_9-Hpode	-0.339 (-0.669, -0.099)	<0.001	<0.001	-0.139 (-0.34, -0.01)	0.036	0.416	41
g_Odoribacter	intestinal_alpha-Linolenic acid	-0.494 (-0.876, -0.165)	<0.001	<0.001	-0.125 (-0.4, -0.022)	0.032	0.830	25.3
s_Alistipes_sp_58_9_plus	intestinal_alpha-Linolenic acid	-0.22 (-0.363, -0.085)	0.004	0.004	-0.049 (-0.186, -0.007)	0.032	0.165	22.3
s_Alistipes_sp_58_9_plus	intestinal_N2-gamma-Glutamylglutamine	-0.22 (-0.37, -0.098)	<0.001	<0.001	-0.061 (-0.144, -0.003)	0.036	0.173	28
s_Alistipes_sp_58_9_plus	intestinal_Stearic acid	-0.22 (-0.346, -0.085)	<0.001	<0.001	-0.062 (-0.186, -0.003)	0.024	0.133	28.1
s_Alistipes_sp_58_9_plus	intestinal_(±)-2-Hydroxy-4-(methylthio)butanoic acid	-0.22 (-0.353, -0.091)	<0.001	<0.001	-0.073 (-0.146, -0.003)	0.044	0.176	33
s_Alistipes_sp_	intestinal_alpha-Linolenic acid	-0.294 (-0.452, -0.135)	<0.001	<0.001	-0.06 (-0.275, -0.004)	0.04	0.120	20.4
s_Alistipes_sp_	intestinal_N2-gamma-Glutamylglutamine	-0.294 (-0.463, -0.145)	<0.001	<0.001	-0.074 (-0.164, -0.001)	0.048	0.133	25.3
s_Alistipes_onderdonkii	intestinal_alpha-Linolenic acid	-0.332 (-0.649, -0.122)	<0.001	<0.001	-0.099 (-0.441, -0.025)	0.028	0.411	29.8
s_Alistipes_onderdonkii	intestinal_N2-gamma-Glutamylglutamine	-0.332 (-0.679, -0.093)	<0.001	<0.001	-0.118 (-0.257, -0.008)	0.028	0.411	35.7
s_Alistipes_onderdonkii	intestinal_Staurosporine	-0.332 (-0.65, -0.113)	<0.001	<0.001	-0.088 (-0.213, -0.001)	0.04	0.411	26.5
s_Alistipes_onderdonkii	intestinal_Stearic acid	-0.332 (-0.662, -0.106)	<0.001	<0.001	-0.123 (-0.353, -0.003)	0.036	0.411	37.2
s_Alistipes_onderdonkii	intestinal_9-Hpode	-0.332 (-0.681, -0.108)	<0.001	<0.001	-0.133 (-0.352, -0.016)	0.016	0.411	40

	s_Alistipes_shahii	intestinal_3a,6a,7b-Trihydroxy-5b-cholanoic acid	-0.461 (-0.81, -0.145)	<0.001	<0.001	-0.113 (-0.284, -0.002)	0.044	0.494	24.5
	s_Alistipes_shahii	intestinal_alpha-Linolenic acid	-0.461 (-0.848, -0.163)	<0.001	<0.001	-0.121 (-0.569, -0.018)	0.024	0.494	26.2
	s_Alistipes_shahii	intestinal_N2-gamma-Glutamylglutamine	-0.461 (-0.837, -0.192)	<0.001	<0.001	-0.149 (-0.315, -0.004)	0.04	0.494	32.3
	s_Alistipes_shahii	intestinal_Stearic acid	-0.461 (-0.817, -0.151)	0.004	0.004	-0.158 (-0.422, -0.001)	0.048	0.494	34.2
	s_Alistipes_shahii	intestinal_9-Hpode	-0.461 (-0.905, -0.199)	<0.001	<0.001	-0.166 (-0.389, -0.018)	0.032	0.494	36.1
	s_Alistipes_timonensis	intestinal_3a,6a,7b-Trihydroxy-5b-cholanoic acid	-0.353 (-0.707, -0.103)	<0.001	<0.001	-0.099 (-0.269, -0.002)	0.04	0.360	27.9
	s_Alistipes_timonensis	intestinal_alpha-Linolenic acid	-0.353 (-0.688, -0.118)	<0.001	<0.001	-0.106 (-0.524, -0.019)	0.028	0.360	30
	s_Alistipes_timonensis	intestinal_N2-gamma-Glutamylglutamine	-0.353 (-0.69, -0.108)	<0.001	<0.001	-0.129 (-0.271, -0.005)	0.036	0.360	36.5
	s_Alistipes_timonensis	intestinal_Staurosporine	-0.353 (-0.731, -0.126)	<0.001	<0.001	-0.096 (-0.251, -0.004)	0.032	0.360	27.2
	s_Alistipes_timonensis	intestinal_Stearic acid	-0.353 (-0.727, -0.112)	<0.001	<0.001	-0.135 (-0.373, -0.007)	0.04	0.360	38.2
	s_Alistipes_timonensis	intestinal_9-Hpode	-0.353 (-0.696, -0.132)	<0.001	<0.001	-0.146 (-0.398, -0.013)	0.016	0.360	41.3
	s_Alistipes_sp_An66	intestinal_3a,6a,7b-Trihydroxy-5b-cholanoic acid	-0.329 (-0.664, -0.127)	0.004	0.004	-0.087 (-0.261, -0.005)	0.036	0.432	26.3
	s_Alistipes_sp_An66	intestinal_alpha-Linolenic acid	-0.329 (-0.606, -0.128)	<0.001	<0.001	-0.09 (-0.382, -0.013)	0.024	0.432	27.4
	s_Alistipes_sp_An66	intestinal_N2-gamma-Glutamylglutamine	-0.329 (-0.627, -0.126)	<0.001	<0.001	-0.109 (-0.245, -0.016)	0.02	0.432	33.3
	s_Alistipes_sp_An66	intestinal_Staurosporine	-0.329 (-0.679, -0.094)	0.012	0.012	-0.084 (-0.209, -0.004)	0.032	0.432	25.5
brain_De	f_Erysipelotrichaceae	intestinal_Cortisone	0.075 (0.002, 0.145)	0.040	0.060	0.06 (0.018, 0.116)	<0.001	<0.001	79.9
oxyadeno sine	f_Erysipelotrichaceae	intestinal_Lauroyl diethanolamide	0.075 (0.003, 0.141)	0.048	0.060	0.059 (0, 0.137)	0.044	0.996	79.1
monophos	c_Erysipelotrichia	intestinal_Cortisone	0.078 (0.007, 0.145)	0.036	0.064	0.063 (0.021, 0.123)	0.004	0.288	80.6
phate	o_Erysipelotrichales	intestinal_Cortisone	0.078 (0.003, 0.159)	0.040	0.064	0.063 (0.015, 0.13)	0.008	0.576	80.6

o_Erysipelotrichales	intestinal_Lauroyl diethanolamide	0.078 (0.002, 0.147)	0.048	0.064	0.063 (0.004, 0.146)	0.024	0.864	81.1
s_Erysipelotrichaceae_bacterium	intestinal_Cortisone	0.084 (0.011, 0.15)	0.024	0.037	0.069 (0.018, 0.138)	0.004	0.288	82.1
s_Erysipelotrichaceae_bacterium	intestinal_Tolmetin	0.084 (0.018, 0.14)	0.020	0.037	0.046 (0.009, 0.098)	0.016	0.576	55
g_Alistipes	intestinal_Cortisone	-0.079 (-0.151, -0.01)	0.024	0.059	-0.042 (-0.104, -0.017)	0.02	0.648	52.9
g_Faecalibaculum	intestinal_Cortisone	0.047 (0.002, 0.091)	0.040	0.056	0.033 (0.011, 0.065)	0.004	0.288	68.5
g_Faecalibaculum	intestinal_Lauroyl diethanolamide	0.047 (0.005, 0.084)	0.036	0.056	0.032 (0.004, 0.068)	0.028	0.864	67
g_Faecalibaculum	intestinal_Tolmetin	0.047 (0.002, 0.09)	0.040	0.056	0.021 (0, 0.053)	0.048	0.864	43.5
s_Faecalibaculum_rodentium	intestinal_Cortisone	0.047 (0.008, 0.091)	0.012	0.052	0.033 (0.014, 0.065)	0.004	0.288	68.5
s_Muribaculaceae_bacterium_Isolate_080_Janvier	intestinal_Cortisone	0.163 (0.004, 0.284)	0.044	0.080	0.071 (0.019, 0.166)	0.008	0.576	43.8
s_Muribaculaceae_bacterium_Isolate_002_NCI	intestinal_Cortisone	-0.152 (-0.317, -0.018)	0.032	0.044	-0.087 (-0.205, -0.009)	0.036	0.964	57.1
s_Muribaculaceae_bacterium_Isolate_002_NCI	intestinal_Lauroyl diethanolamide	-0.152 (-0.359, -0.026)	0.016	0.044	-0.1 (-0.248, -0.012)	0.012	0.864	66.1
o_Coriobacteriales	intestinal_Cortisone	0.05 (0.011, 0.104)	0.020	0.035	0.035 (0.013, 0.087)	0.028	0.461	70.4
o_Coriobacteriales	intestinal_Lauroyl diethanolamide	0.05 (0.007, 0.097)	0.016	0.035	0.029 (0.003, 0.068)	0.032	0.461	58.9
f_Odoribacteraceae	intestinal_Cortisone	-0.116 (-0.203, -0.033)	<0.001	<0.001	-0.059 (-0.135, -0.013)	0.024	0.982	50.7
f_Odoribacteraceae	intestinal_Lauroyl diethanolamide	-0.116 (-0.204, -0.026)	0.020	0.024	-0.064 (-0.157, -0.005)	0.044	0.982	55
f_Coriobacteriaceae	intestinal_Cortisone	0.051 (0.01, 0.097)	0.016	0.034	0.035 (0.016, 0.078)	0.008	0.144	69.1
f_Coriobacteriaceae	intestinal_Lauroyl diethanolamide	0.051 (0.009, 0.098)	0.020	0.034	0.031 (0.006, 0.071)	0.028	0.403	60
s_Coriobacteriaceae_bacterium	intestinal_Cortisone	0.027 (0.006, 0.051)	0.020	0.021	0.017 (0.008, 0.05)	0.012	0.173	65.2
s_Coriobacteriaceae_bacterium	intestinal_Lauroyl diethanolamide	0.027 (0.006, 0.049)	0.012	0.015	0.015 (0.003, 0.034)	0.012	0.173	55.9

s_Coriobacteriaceae_bacterium	intestinal_Tolmetin	0.027 (0.009, 0.047)	0.004	0.014	0.021 (0.006, 0.041)	<0.001	<0.001	77.6
s_Alistipes_senegalensis	intestinal_Cortisone	-0.09 (-0.159, -0.005)	0.032	0.043	-0.045 (-0.104, -0.015)	0.028	0.672	49.4
s_Alistipes_finegoldii	intestinal_Cortisone	-0.08 (-0.138, -0.007)	0.040	0.054	-0.041 (-0.106, -0.019)	0.012	0.720	50.7
g_Odoribacter	intestinal_Cortisone	-0.118 (-0.208, -0.026)	0.032	0.033	-0.058 (-0.135, -0.014)	0.016	0.965	49.5
g_Odoribacter	intestinal_Lauroyl diethanolamide	-0.118 (-0.202, -0.032)	0.016	0.024	-0.063 (-0.148, -0.008)	0.032	0.965	53.1
s_Alistipes_sp_58_9_plus	intestinal_Cortisone	-0.037 (-0.076, -0.002)	0.036	0.092	-0.025 (-0.078, -0.01)	0.004	0.288	67.3
s_Alistipes_sp_58_9_plus	intestinal_Lauroyl diethanolamide	-0.037 (-0.083, -0.002)	0.036	0.092	-0.022 (-0.051, -0.001)	0.044	0.634	61.3
s_Alistipes_sp_	intestinal_Cortisone	-0.059 (-0.103, -0.012)	0.028	0.029	-0.031 (-0.083, -0.01)	0.028	0.403	51.4
s_Alistipes_sp_	intestinal_Lauroyl diethanolamide	-0.059 (-0.102, -0.015)	0.008	0.023	-0.033 (-0.066, -0.007)	0.012	0.384	55.5
s_Alistipes_onderdonkii	intestinal_Cortisone	-0.077 (-0.13, -0.017)	0.020	0.054	-0.039 (-0.088, -0.02)	<0.001	<0.001	50.6
s_Alistipes_shahii	intestinal_Cortisone	-0.1 (-0.177, -0.017)	0.020	0.052	-0.051 (-0.12, -0.022)	0.004	0.288	51
s_Alistipes_timonensis	intestinal_Cortisone	-0.086 (-0.153, -0.011)	0.020	0.043	-0.042 (-0.094, -0.015)	<0.001	<0.001	49.1
s_Alistipes_timonensis	intestinal_Lauroyl diethanolamide	-0.086 (-0.145, -0.014)	0.028	0.043	-0.036 (-0.087, -0.002)	0.048	0.883	41.6
s_Alistipes_sp_An66	intestinal_Cortisone	-0.074 (-0.132, -0.007)	0.012	0.050	-0.037 (-0.095, -0.018)	0.008	0.576	49.8
s_Alistipes_sp_An66	intestinal_Lauroyl diethanolamide	-0.074 (-0.133, -0.01)	0.028	0.050	-0.033 (-0.079, -0.002)	0.032	0.853	44.3
